# Ipecac alkaloid biosynthesis in two evolutionarily distant plants

**DOI:** 10.1038/s41589-025-01926-z

**Published:** 2025-06-03

**Authors:** Maite Colinas, Clara Morweiser, Olivia Dittberner, Bianca Chioca, Ryan Alam, Helena Leucke, Yoko Nakamura, Delia Ayled Serna Guerrero, Sarah Heinicke, Maritta Kunert, Jens Wurlitzer, Kerstin Ploss, Benke Hong, Veit Grabe, Adriana A. Lopes, Sarah E. O’Connor

**Affiliations:** 1 Department of Natural Product Biosynthesis, Max Planck Institute for Chemical Ecology, Jena, Germany; 2 Universidade de Ribeirão Preto, Departamento de Biotecnologia, Ribeirão Preto, Brazil; 3 Research Group NMR and Department of Natural Product Biosynthesis, Max Planck Institute for Chemical Ecology, Jena, Germany; 4 Microscopy Imaging Service, Max Planck Institute for Chemical Ecology, Jena, Germany

**Keywords:** Biochemistry, Plant sciences, Natural products, Biosynthesis, Enzymes

## Abstract

Ipecac alkaloids are medicinal monoterpenoid-derived tetrahydroisoquinoline alkaloids found in two distantly related plants: *Carapichea ipecacuanha* (Gentianales) and *Alangium salviifolium* (Cornales). Here we provide evidence suggesting that both plants initiate ipecac alkaloid biosynthesis through a nonenzymatic Pictet–Spengler reaction and we elucidate the biosynthetic fate of both the 1*R* and 1*S* stereoisomers that are produced in this nonstereoselective reaction. Although the biosynthesis of the 1*S*-derived protoemetine proceeds according to the same chemical logic in both species, each plant uses a distinct monoterpene precursor. Phylogenetic analyses show examples of independent pathway evolution through parallel and convergently evolved enzymes. This work provides insight into how nature can capitalize on highly reactive starting substrates and the manner in which multistep pathways can arise and lays the foundation for metabolic engineering of these important medicinal compounds.

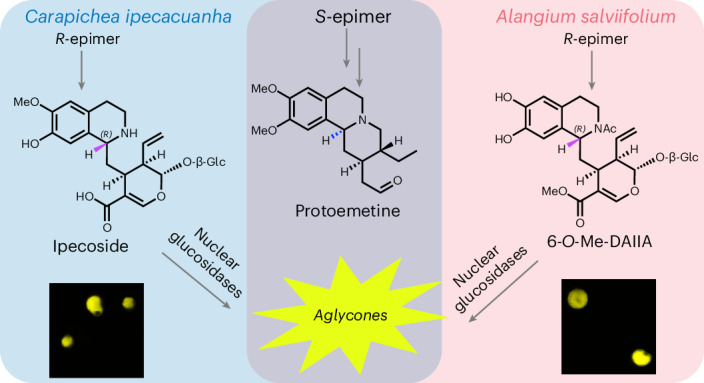

## Abstract (web summary)

Ipecac alkaloids are plant-derived compounds historically used to induce vomiting. Here the authors elucidate how two evolutionary distant plant species have evolved to produce the same ipecac alkaloid.

## Main

**Fig. 1:**
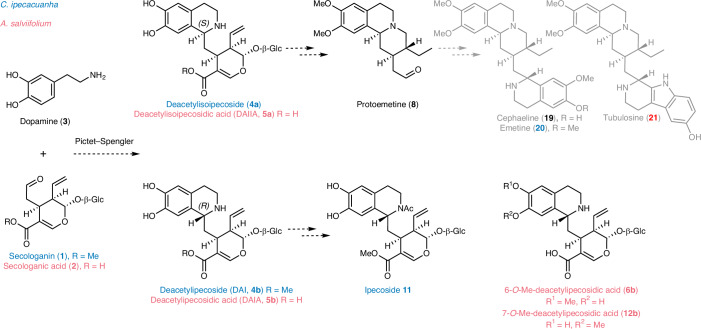
Ipecac alkaloid biosynthesis in *C.* *ipecacuanha* and *A.* *salviifolium* based on the literature. Either secologanin **1** or secologanic acid **2** is coupled with dopamine **3** in a Pictet–Spengler reaction to yield deacetylisoipecoside DAII (*S*-epimer) **4a** and deacetylipecoside DAI (*R*-epimer) **4b** or the respective acids deacetylisoipecosidic acid DAIIA (*S*-epimer) **5a** and deacetylipecosidic acid DAIA **5b**. Derivatized forms of the *R*-epimer are the *N*-acetylated ipecoside found in *C.* *ipecacuanha* and 6-*O*-Me-deacetylipecosidic acid or 7-*O*-Me-deacetylipecosidic acid found in *A.* *salviifolium*. The *S*-epimer undergoes a series of reactions including methylations, deglycosylation, reduction and, in the case of deacetylisoipecoside, deesterification to form protoemetine **8**, which is then derivatized to form downstream alkaloids in both plants as shown. Compounds specific to *C.* *ipecacuanha* are shown in blue; compounds specific to *A.* *salviifolium* are shown in magenta.

Plants produce an enormous diversity of natural products or specialized metabolites. Although natural product pathways are typically lineage specific, in some cases, evolutionarily distant plants independently evolved pathways to synthesize the same molecule^1,2^. Examples include glucosinolates^3^, benzoxazinoids^4^, caffeine^5–7^, cannabinoids^8^ and cardenolides^9–11^. Ipecac alkaloids are monoterpenoid-derived tetrahydroisoquinoline alkaloids that occur in *Carapichea ipecacuanha* (Gentianales) and *Alangium salviifolium* (Cornales). Both species are known medicinal plants: ipecac syrup made from *C.* *ipecacuanha* rhizomes has been used as a vomit-inducing medicine in cases of intoxication, while *A.* *salviifolium*, also known as Ankol(a), is used as an emetic and to treat a variety of diseases in traditional ayurvedic medicine^12,13^. The active emetic ingredients are the tetrahydroisoquinoline alkaloid pathway products, cephaeline and emetine, both derived from protoemetine (Fig. 1). Additionally, anticancer and antimalarial activities have been described for other protoemetine-derived alkaloids such as tubulosine^14,15^. Because Cornales and Gentianales are estimated to have diverged approximately 150 million years ago^16^, these plants serve as excellent models to study the evolution of a complex medicinal alkaloid biosynthesis pathway.

In *C.* *ipecacuanha*, ipecac alkaloid biosynthesis begins with a Pictet–Spengler reaction that couples the monoterpenoid secologanin **1** with dopamine to generate the initial tetrahydroisoquinoline scaffold. Conflicting studies suggest that either secologanin **1** or secologanic acid **2** can be similarly conjugated with dopamine **3** in *A.* *salviifolium*^17–20^, although secologanic acid has been shown to be the monoterpene precursor in indole alkaloid biosynthesis in *Camptotheca acuminata*, also in the Cornales lineage^21,22^. Although previously identified enzymes that catalyze the Pictet–Spengler reaction are stereoselective, unusually, both *S* and *R* stereoisomers of the initial tetrahydroisoquinoline Pictet–Spengler product are observed in *C.* *ipecacuanha* (deacetylisoipecoside DAII **4a** (*S-*epimer) and deacetylipecoside DAI **4b** (*R-*epimer))^23^ (Fig. 1). Similarly, methylated forms of both *S* and *R* stereoisomers of the corresponding acids (deacetylisoipecosidic acid DAIIA **5a** (*S-*epimer) and deacetylipecosidic acid DAIA **5b** (*R-*epimer)) are observed in *A.* *salviifolium*^17,24^. Early work suggested that a Pictet–Spenglerase enzyme is present in *A.* *salviifolium* but a gene encoding this enzyme has never been identified^18,25^. In both *C.* *ipecacuanha* and *A.* *salviifolium*, the *S*-epimers are converted by *O*-methylation, deglycosylation, reduction and decarboxylation to protoemetine **8**, an ipecac alkaloid common to both species^26–28^. In *C.* *ipecacuanha*, protoemetine is converted into cephaeline and emetine^26^, whereas, in *A.* *salviifolium*, proteoemetine is converted to cephaeline, alangimarckine and tubulosine^29,30^. In *C.* *ipecacuanha*, the *R*-epimer is *N*-acetylated to ipecoside **11** (ref. ^31^), whereas, in *A.* *salviifolium*, the *R-*epimer is converted to 6-*O*-Me-DAIA **6b** and 7-*O*-Me-DAIA **12b** (ref. ^24^) (Fig. 1).

A few ipecac alkaloid biosynthetic genes from *C.* *ipecacuanha* encoding glucosidases and *O*-methyltransferases (OMTs) have been reported^32–34^ but the biosynthesis of ipecac alkaloids remains largely unknown. Here, we report the complete discovery of protoemetine biosynthesis in both *C.* *ipecacuanha* and *A.* *salviifolium*, which we show proceeds through an unexpected order of enzymatic reactions. We provide evidence that the Pictet–Spengler reaction initiating the pathway can occur spontaneously in the vacuole, which would explain the presence of both 1*R* and 1*S* stereoisomers in these plants. While protoemetine is derived from the 1*S-*epimer, we also identify biosynthetic genes that derivatize the 1*R-*epimer in *C.* *ipecacuanha* and *A.* *salviifolium*. Phylogenetic analyses suggest that the enzymes that convert DAII(A) to protoemetine evolved independently in *C.* *ipecacuanha* and *A.* *salviifolium* through means of parallel and convergent evolution. This collection of metabolic pathways provides a striking example of evolution of complex, medicinally important compounds in phylogenetically distant plants.

## Results

### Metabolite profiling of *C.ipecacuanha* and *A.salviifolium*

**Fig. 2:**
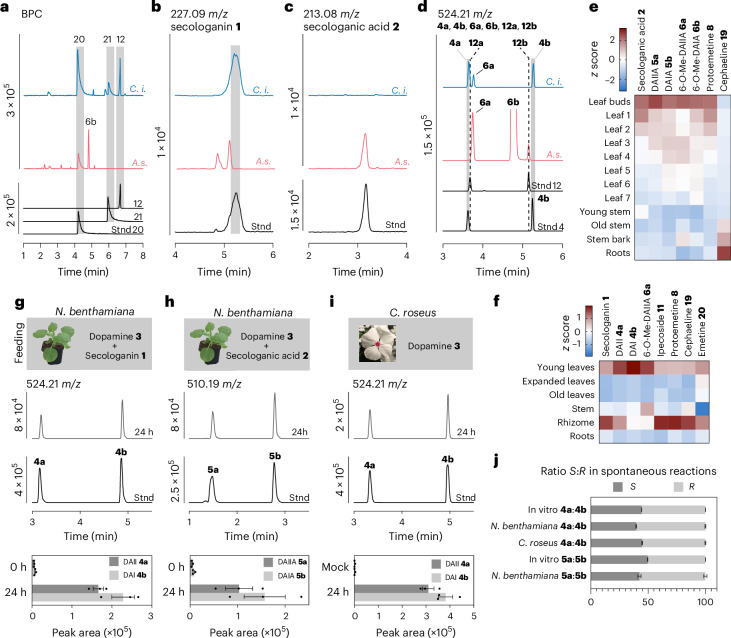
Metabolite profiling and evidence for a nonenzymatic Pictet–Spengler reaction in *C.* *ipecacuanha* and *A.* *salviifolium.* **a**–**d**, LC–MS analysis of *C.* *ipecacuanha* rhizome extracts (blue) and *A.* *salviifolium* root extracts (magenta). **a**, Base peak chromatograms (BPC) of extracts at 2 mg FW ml^−1^ (Supplementary Fig. 3). **b**–**d**, EICs of extracts at 10 mg FW ml^−1^. **b**, The EIC of secologanin ([M − glucose + H]^+^ = 227.09 *m/z*) indicates that it is only found in *C.* *ipeacuanha*. **c**, The EIC of secologanic acid ([M − glucose + H]^+^ = 213.08 *m/z*) indicates that it accumulates only in *A.* *salviifolium* (Supplementary Fig. 4). **d**, DAI/I (**4a**/**4b**) and *O*-Me-DAI/IA have identical *m/z* [M + H]^+^ = 524.21 but are distinguished by different retention times and MS^2^ fragmentation (Supplementary Fig. 5). DAI/I (**4a**/**4b**) is exclusively found in *C.* *ipecacuanha* and not in *A.* *salviifolium*, where 6-*O*-Me-DAIA and 7-*O*-Me-DAIA (1*R*) (**6b** and **12b**) accumulate in large amounts. A putative intermediate en route to protoemetine biosynthesis, 6-*O*-Me-DAIIA **6a** (1*S*), is found in both species (Supplementary Fig. 5). Assignment of **4a** as 1*S* and **4b** as 1*R* was confirmed by NMR spectroscopy (Supplementary Figs. 24 and 25 and Supplementary Tables 2 and 3). Stnd, standard. **e**,**f**, Tissue-specific relative distribution of selected ipecac alkaloids in *C.* *ipecacuanha* (**e**) and *A.* *salviifolium* (**f**). Heat maps depict *z* scores of average peak areas of three biological replicates for each metabolite (Supplementary Figs. 1 and 2). **g**–**i**, LC–MS peak areas are shown as bars of the mean of three biological replicates; error bars denote the s.e.m. and dots are single data points. **g**,**h**, Reaction products DAI/I or DAI/IA from coupling reaction of dopamine with secologanin (**g**) or secologanic acid (**h**) are observed after within 24 h after infiltration into *N.* *benthamiana* leaves. **i**, Infiltration of dopamine to flower petals of the natural secologanin producer *C.* *roseus* also leads to the appearance of DAI/I within 24 h. **j**, Ratio of *S*-epimers to *R-*epimers upon reaction in vitro or in planta. LC–MS peak areas of epimers are shown as bars of the mean as relative parts of their sum; error bars denote the s.e.m. Source data

We isolated *C.* *ipecacuanha* and *A.* *salviifolium* tissues for RNA sequencing and for metabolomic profiling. Protoemetine (**8**) (1*S* stereoisomer)-derived alkaloids were found in C*.* *ipecacuanha* rhizomes (cephaeline and emetine) and in *A.* *salviifolium* root (cephaeline) (Fig. 2a). The 1*R-*derived products were also found in *C.* *ipecacuanha* rhizomes (ipecoside, **11**) and *A.* *salviifolium* root (6-*O*-Me-DAIA, **6b**) (Fig. 2a,d). Tissue-specific metabolite profiling (Fig. 2e,f and Supplementary Figs. 1 and 2; comparisons to standards in Supplementary Figs. 3–7) revealed that *C.* *ipecacuanha* accumulates similar amounts of ipecac alkaloids in both young leaves and rhizomes (Fig. 2f). In contrast, *A.* *salviifolium* pathway intermediates up to protoemetine **8** are detected in high levels in leaf buds but cephaeline only accumulates in roots and barks of older stems (Fig. 2e). We used these metabolite profiles to guide the search for protoemetine gene candidates in the corresponding RNA-seq datasets.

### A Pictet–Spengler reaction in ipecac alkaloid biosynthesis

**Extended Data Fig. 1:**
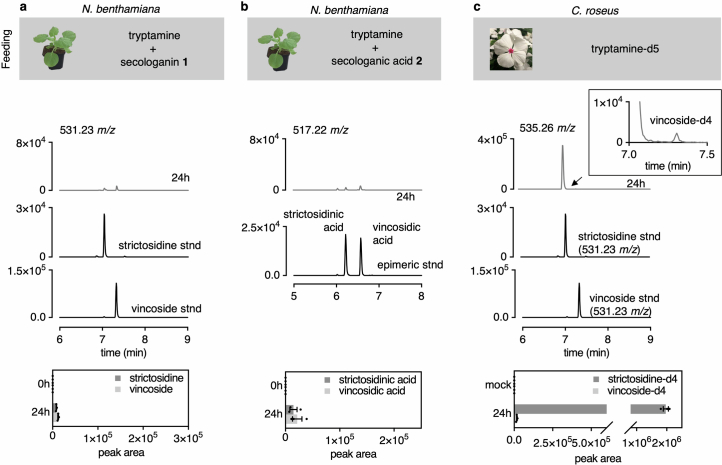
Assessing in planta coupling of tryptamine with secologanin or secologanic acid. Small amounts of reaction products strictosidine (*3S*)/vincoside (*3* *R*) or strictosidinic acid (*3S*)/vincosidic acid (*3* *R*) of coupling reaction of tryptamine with secologanin (**a**) or secologanic acid (**b**) are observed within 24 hours after infiltration into *N. benthamiana* leaves. For comparison to the reaction with dopamine, the y axes showing signal intensity or peak areas were scaled identically to the axes of Fig. 2**g-h** (main text). **c**, Infiltration of tryptamine-d5 to flower petals of the natural secologanin producer *C. roseus* leads to large amounts of strictosidine-d4 but only traces of vincoside-d4. The endogenous *C. roseus* Strictosidine Synthase (STR) exclusively produces strictosidine, therefore the trace amount of vincoside can be attributed to non-enzymatic coupling. Taken together, the non-enzymatic reaction with tryptamine appears to occur much less readily than with dopamine. Stnd, standard. LC-MS peak areas are shown as bars of the mean of three biological replicates, error bars are standard error of the mean.

In the metabolite profiling experiments described above, we also noted that secologanin (**1)** is only observed in *C.* *ipecacuanha* while secologanic acid (**2**) is observed only in *A.* *salviifolium* (Fig. 2b,c). This suggests that *C.* *ipecacuanha* uses secologanin in ipecac alkaloid biosynthesis while *A.* *salviifolium* uses the corresponding acid. Consistent with this, DAII **4a** and DAI **4b** are detected in *C.* *ipecacuanha* but not *A.* *salviifolium* (Fig. 2d). Unexpectedly, we noticed that when we infiltrated the starting substrates (secologanin or secologanic acid together with dopamine) into *Nicotiana benthamiana* leaves, the corresponding Pictet–Spengler products DA(I)I or DAI(I)A (both 1*S* and 1*R* stereoisomers) were formed within 24 h (Fig. 2g,h). Moreover, when *Catharanthus roseus* flower petals, which contain endogenous secologanin **1** but do not produce ipecac alkaloids, were infiltrated with dopamine, accumulation of DAI **4b** (1*R*) and DAII **4a** (1*S*) was also observed after 24 h (Fig. 2i), clearly demonstrating that this nonenzymatic reaction can take place in planta. We were unable to detect enzymatic activity in *C.* *ipecacuanha* or *A.* *salviifolium* that impacted the rate or stereoselectivity of this nonenzymatic reaction (Supplementary Fig. 8). Dopamine is a highly activated substrate for the Pictet–Spengler reaction, reacting rapidly in a nonstereoselective manner under mild acidic conditions^35^. In contrast, we showed that Pictet–Spengler reactions with tryptamine, either in *N.* *benthamiana* leaves or in *C.* *roseus* flower petals, resulted in much lower levels of nonenzymatically formed coupled product, suggesting that dopamine is chemically activated compared to tryptamine (Extended Data Fig. 1). Although enzymes that catalyze the Pictet–Spengler reaction with dopamine are known, these enzymes are stereoselective^36^. Because *C.* *ipecacuanha* and *A.* *salviifolium* produce products derived from both stereoisomers and because we demonstrated that this reaction can occur in two different plants lacking a dedicated enzyme, we hypothesize that this Pictet–Spengler coupling may occur nonenzymatically and nonstereoselectively in these plants.

### Ipecac alkaloid biosynthetic gene candidates

**Extended Data Fig. 2:**
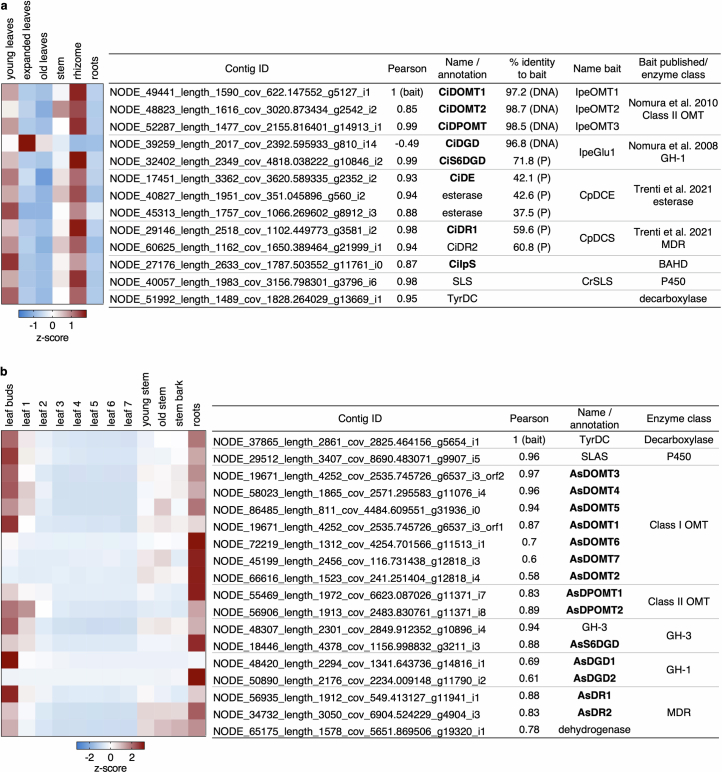
Selection of pathway gene candidates. **a**, selection of *C. ipecacuanha* candidates by searching for genes similar to previously published active pathway enzymes^12,13^. For each of the published sequences the contig with the highest sequencing homology ( > 96%) at cDNA level was considered to be identical to the published enzyme. *CiDOMT1* was used as a bait for Pearson correlation. Candidates for deesterification and reduction were found by homology-based search and coexpression with *CiDOMT1* (described in in the methods). A highly coexpressed gene predicted to encode for an acetyltransferase was picked as a candidate for ipecoside synthase. **b**, selection of *A. salviifolium* candidates was performed by coexpression analysis using orthologous and likely conserved sequences of enzymes supplying the direct precursor genes *TyrDC* and *SLS*, as baits, combined with filtering for predicted functional annotations for *O*-methyltransferases, glucosidases and dehydrogenases. Additionally, we considered candidates that showed either root or leaf bud specific expression (*AsDGDs*). Additional root specific *AsDOMT5-7* were also identified. For both species gene expression patterns are consistent with metabolite abundance (see Fig. 2e-f). Heatmaps depict z-scores of TMM normalized CPM values for each gene.

After the Pictet–Spengler reaction, protoemetine is generated from DAII (**4a**) or DAIIA (**5a**) (1*S-*epimers) by *O*-methylation, deglycosylation, reduction and, in the case of *C.* *ipecacuanha*, deesterification (Fig. 1). The 1*R*-epimers (DAIIA **5a** or DAIA **5b**) are either acetylated to form ipecoside (**11**) in *C.* *ipecacuanha* or *O*-methylated to form 6-*O*-Me-DAIA **6b** and 7-*O*-Me-DAIA **12b** in *A.* *salviifolium*. We searched for genes in our generated transcriptomes that encode enzymes that could catalyze these reactions. First, we identified the previously published *C.* *ipecacuanha OMT* genes (renamed *CiDOMT1*, *CiDOMT2* and *CiDPOMT*) and glucosidase (*CiDGD*)^32–34^. Expression levels of the three *OMT* genes in our *C.* *ipecacuanha* transcriptome were highest in young leaves and rhizome (Extended Data Fig. 2a), consistent with the metabolite profile of ipecac alkaloid accumulation (Fig. 2f). Using the previously discovered *CiDOMT1* gene as bait for coexpression analysis, we identified two reductase and three esterase transcripts. Surprisingly, the previously reported glucosidase gene, *CiDGD*, was not coexpressed with the *CiOMT* genes (−0.49 Pearson correlation with *CiDOMT1*); however, among the highly coexpressed contigs, a new glucosidase, named *CiS6DGD* (71.4% amino acid identity to CiDGD), was found. A gene annotated as an acetyl transferase was also identified. Additionally, we noticed that orthologs of known precursor-generating enzymes secologanin synthase (*SLS*; which catalyzes the final step of secologanin biosynthesis) and tyrosine decarboxylase (*TyrDC*; predicted to be involved in dopamine biosynthesis) were also tightly coexpressed with *CiDOMT1*.

No pathway genes from *A.* *salviifolium* have been reported and the *A.* *salviifolium* transcriptome did not contain orthologs of the previously published *CiOMT* genes. Therefore, we mined the *A.* *salviifolium* transcriptome for orthologs of putative precursor genes *TyrDC* and *SLAS* (secologanic acid synthase), which catalyzes the last step of secologanic acid biosynthesis^37^. The expression profile of these two identified orthologs was highest in leaf buds and roots (Extended Data Fig. 2b), consistent with metabolite data (Fig. 2e). Using *TyrDC* as bait for coexpression analysis, we identified coexpressed genes that had functional annotations consistent with OMT, dehydrogenase and glycosyl hydrolase activity. Because metabolite data indicated that *A.* *salviifolium* uses secologanic acid to form the initial Pictet–Spengler product DAIIA, we predicted that an esterase would not be required for protoemetine biosynthesis in this plant.

### Comparative discovery of protoemetine biosynthesis

**Fig. 3:**
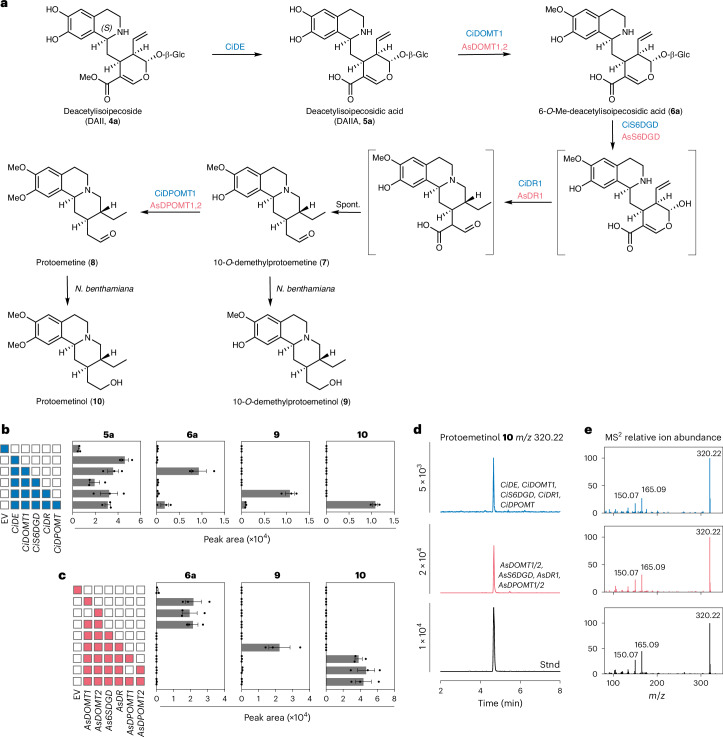
Discovery of *C.* *ipecacuanha* and *A.* *salviifolium* protoemetine biosynthetic genes. **a**, The complete pathway leading to protoemetine (**8**). Molecules in parentheses are hypothesized unstable intermediates that were not detected by LC–MS. Aldehydes **7** and **8** were only detected in traces by LC–MS; instead, the corresponding alcohols **9** and **10** were detected. Peak identities were confirmed by comparing to standards (full MS characterization in Supplementary Figs. 5 and 7; NMR characterization of protoemetine standard in Supplementary Fig. 26), except 10-*O*-demethylprotoemetinol, which was identified on the basis of MS^2^ fragmentation (Supplementary Fig. 11). Spont., spontaenous. **b**, LC–MS peak areas of products in *N.* *benthamiana* upon expression of indicated *C.* *ipecacuanha* pathway genes and infiltration of a synthetically generated mixture of DAII (**4a**) and DAI (**4b**). Data are the mean ± s.e.m. of *n* = 3 biological replicates; dots are single data points. **c**, LC–MS peak areas of products in *N.* *benthamiana* upon expression of indicated *A.* *salviifolium* pathway genes and infiltration of synthetically generated mixture of DAIIA (**5a**) and DAIA (**5b**). Data are the mean ± s.e.m. of *n* = 3 biological replicates; dots are single data points. **d**, The EIC for protoemetinol upon expression of indicated *C.* *ipecacuanha* pathway genes (blue) or *A.* *salviifolium* pathway genes (magenta) and authentic standard (black). **e**, MS^2^ fragmentation for the corresponding peaks shown in **d**, confirming the peak as protoemetinol. Synthesis of the protoemetinol standard is described in the Supplementary Methods. Results of additional gene combinations are shown in Extended Data Figs. 4 and 6. Source data

**Extended Data Fig. 3:**
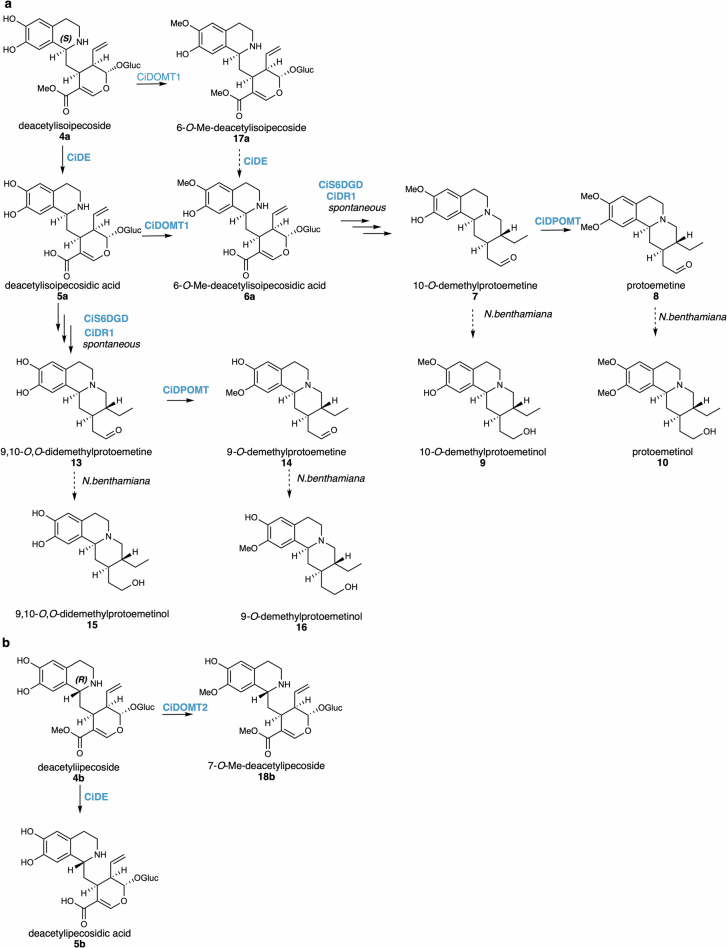
*C. ipecauanha* protoemetine biosynthesis network including possible side branch products. Activities were detected through expression of pathway genes shown in Supplementary Fig. 10. **a**, *S*-epimer derived pathways. **b**, *R*-epimer derived pathways.

**Extended Data Fig. 4:**
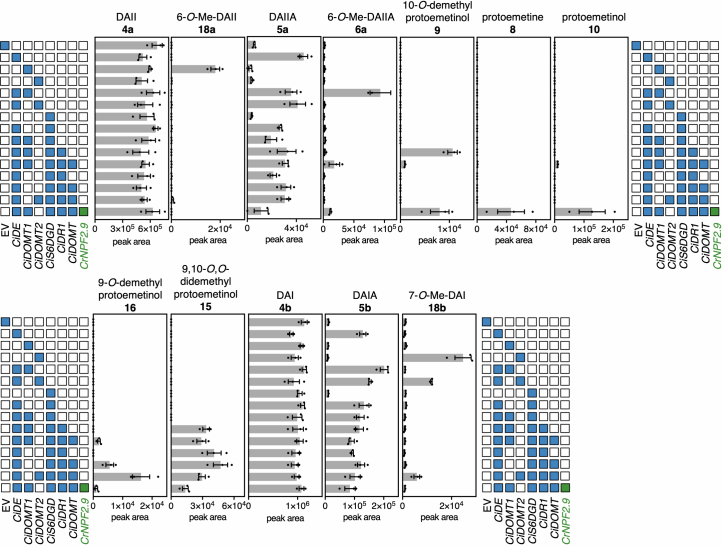
Reconstitution of protoemetine biosynthesis network by expression of *C. ipecacuanha* pathway genes. Data is from the same experiment shown in Fig. 3. This figure includes the data for Fig. 3 and additional gene combinations. Additionally, expression was combined with *Catharanthus roseus* strictosidine exporter CrNPF2.9. LC-MS peak areas are shown as bars of the mean of three biological replicates, error bars are standard error of the mean.

**Extended Data Fig. 5:**
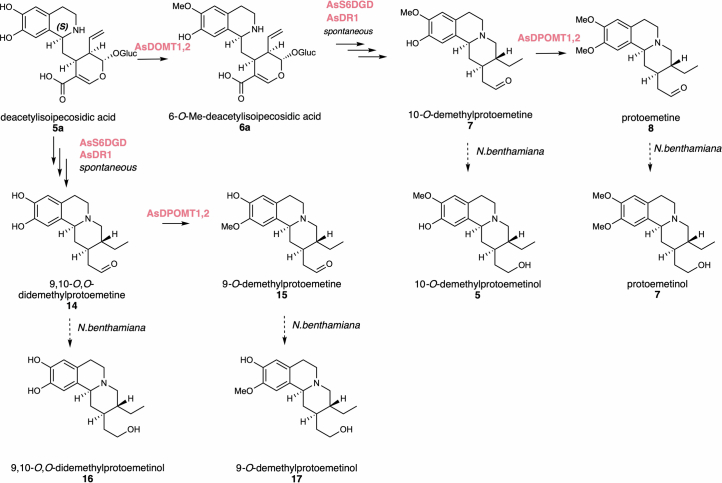
*A. salviifolium* protoemetine biosynthesis network. Activities were detected through expression of pathway genes shown in Extended Data Fig. 6.

**Extended Data Fig. 6:**
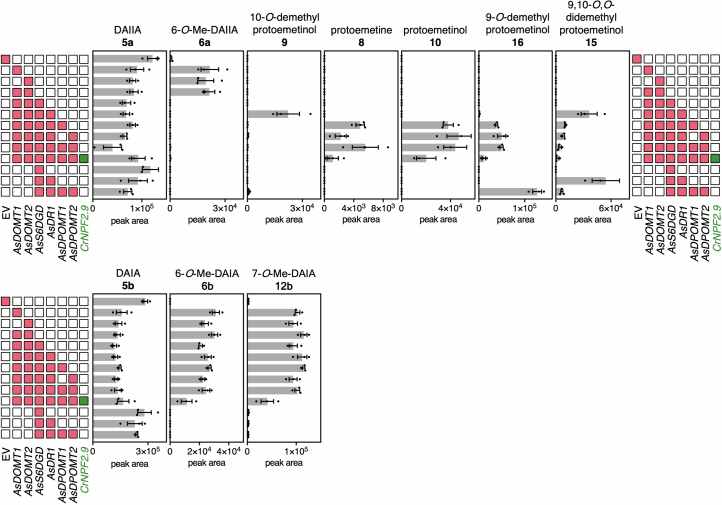
Reconstitution of protoemetine biosynthesis network by expression of *A. salviifolium* pathway genes. Data is from the same experiment shown in Fig. 3. This figure includes the data for Fig. 3 and additional gene combinations. Additionally, expression was combined with *Catharanthus roseus* strictosidine exporter CrNPF2.9. LC-MS peak areas are shown as bars of the mean of three biological replicates, error bars are standard error of the mean.

Transient expression of pathway gene candidates along with infiltration of the starting substrate in *N.* *benthamiana* enabled us to successfully deconvolute the complete protoemetine (**8**) biosynthetic pathway from both *C.* *ipecacuanha* and *A.* *salviifolium* (Fig. 3 and Extended Data Figs. 3–6). Combinatorial expression of the identified gene candidates was followed by assay of individual genes. On the basis of previous proposals and the chemical logic established for other secologanin-derived natural products such as corynantheal (*Cinchona pubescens*)^32,33,38^, we initially hypothesized that, in *C.* *ipecacuanha*, DAII (**4a**) would be deglycosylated, reduced and deesterified, which would in turn lead to spontaneous decarboxylation to form protoemetine. However, because we observed DAIIA (**5a**) and 6-*O*-Me-DAIIA (**6a**) in *C.* *ipecacuanha* (Fig. 2f and Supplementary Fig. 2), we speculated that deglycosylation may happen after deesterification. Indeed, the identified esterase (CiDE) deesterifies the glucosylated intermediate DAII (**4a**) to yield DAIIA (**5a**) (Fig. 3 and Extended Data Fig. 4). In *A.* *salviifolium*, the pathway directly starts with deesterified DAIIA, derived from the Pictet–Spengler condensation of secologanic acid (**2**) with dopamine. Thus, both *C.* *ipecacuanha* and *A.* *salviifolium* use DAIIA (**5a**) as a protoemetine (**8**) intermediate.

DAIIA (**5a**) is then 6-*O*-methylated by CiDOMT1 (previously identified by Nomura) or by AsDOMT1 and AsDOMT2 (Fig. 3). Although an authentic standard is not available, the resulting product has an *m/z* value and tandem mass spectroemetry (MS^2^) consistent with 6-*O*-Me-DAIIA (**6b**) and this product was also detected in extracts of both species (Fig. 2d and Supplementary Fig. 5). We observed that CiS6DGD/AsS6DGD deglycosylated 6-*O*-Me-DAIIA, whereas the pathway could not be reconstituted with the previously reported *C.* *ipecacuanha* glucosidase, *CiDGD* (Fig. 3 and Supplementary Fig. 9). The aglycone generated by CiS6DGD/AsS6DGD is subjected to a two-step reduction, catalyzed in both species by a medium-chain reductase, CiDR1 or AsDR1 (Fig. 3 and Supplementary Fig. 10). Spontaneous decarboxylation is then triggered, yielding 10-*O*-demethylprotoemetine, which is further methylated by CiDPOMT or AsDPOMT1 and AsDPOMT2. Notably, we could only detect trace amounts of the aldehydes 10-*O*-demethylprotoemetine and protoemetine in *N.* *benthamiana* (Extended Data Figs. 4 and 6); instead, we detected the reduced forms 10-*O*-demethylprotoemetinol (identified by *m/z* MS^2^ fragmentation; Supplementary Fig. 11) and protoemetinol (confirmed by comparison with an authentic standard), which is expected because of endogenous *N.* *benthamiana* aldehyde reductases^39,40^. Paralogs of DR (CiDR2 and AsDR2) were also active but resulted in the formation of lower amounts of protoemetinol (Supplementary Fig. 12). Although CiDOMT2 can methylate the 7-hydoxy group of DAI, it did so with low efficiency (Extended Data Fig. 4).

### Discovery of species-specific *R*-epimer pathways

**Fig. 4:**
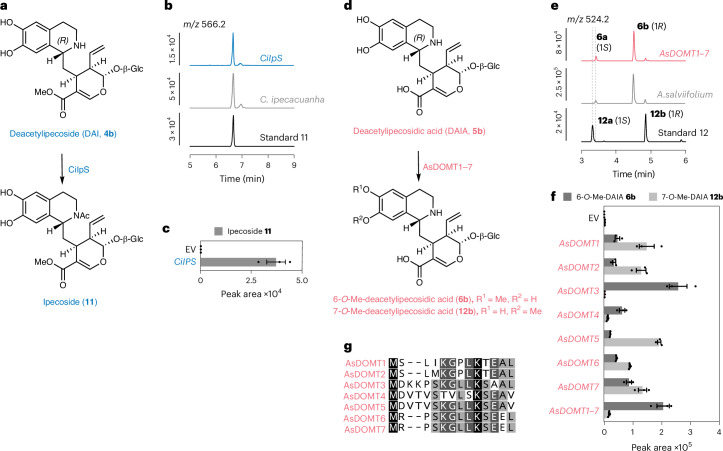
Reconstitution of *R*-epimer pathways specific to *C.* *ipecacuanha* and *A.* *salviifolium* in *N.* *benthamiana.* **a**, *C.* *ipecacuanha* CiIPS *N*-acetylates DAI (**4b**) to yield ipecoside (**11**). **b**, EICs of ipecoside (**11**) (*m/z* 566.22) obtained from *N.* *benthamaiana* expressing *CiIpS* and infiltrated with a synthetic mixture of DAI (**4b**) and DAII (**4a**) (blue, top), native *C.* *ipecacuanha* extract (gray, middle) and authentic standard (black, bottom). **c**, LC–MS peak areas of *N.* *benthamiana* upon expression of CiIpS and infiltration of DAI (**4b**) compared to empty vector (EV) control. Bars show the means of three biological replicates; error bars are the s.e.m. and dots are single data points. **d**, In *A.* *salviifolium* DAIA (**5b**) is *O*-methylated at the 6 or 7 position leading to 6-*O*-Me-DAIA (**6b**) or 7-*O*-Me-DAIA (**12b**) by the closely related enzymes AsDOMT1–AsDOMT7. **e**, EICs from *N.* *benthamaiana* expressing *AsDOMT1–AsDOMT7* and infiltrated with a synthetic mixture of DAIA (**5b**) and DAIIA (**5a**) (magenta, top), native *A.* *salviifolium* extract (gray) and a mixture of synthetic 7-*O*-Me-DAI/IA **12a** and **12b** standards (black, bottom). **f**, Peak areas of products obtained from *N.* *benthamiana* transformed with EV (control) compared to *AsDOMT* expression constructs plus infiltration with a synthetic mixture of DAIA (**5b**) and DAIIA (**5a**). Bars show the means of three biological replicates; error bars are the s.e.m. and dots are single data points. AsDOMT enzymes exhibit different product specificities. **g**, Alignment of the N termini of AsDOMT1–AsDOMT7. Product profiles shown in **f** appear to be consistent with the level of sequence identity at the N terminus. Full alignment is shown in Supplementary Fig. 15. Source data

Our metabolite profiling suggested that the *R*-epimer DAI (**4b**) is acetylated to form ipecoside (**11**) in *C.* *ipecacuanha*, while DAIA (**5b**) is methylated on the 6-hydroxy group (and, to a lesser extent, on the 7-hydroxy group) in *A.* *salviifolium* to form 6-*O*-Me-DAIA or 7-*O*-Me-DAIA (**6b** and **12b**) (Fig. 2d,e and Supplementary Fig. 2b). A gene annotated as a BAHD-type acetyltransferase in the *C.* *ipecacuanha* transcriptome that was highly coexpressed with *CiDOMT1* (Extended Data Fig. 2a) led to formation of ipecoside (as evidenced by comparison with an authentic standard) when expressed in *N.* *benthamiana* along with DAI (**4b**) (Fig. 4a–c). This gene was, thus, named ipecoside synthase (*CiIpS*).

In the *A.* *salviifolium* transcriptome, seven closely related class I *OMT* genes that showed promising expression profiles were tested (Extended Data Fig. 2b). AsDOMT1 and AsDOMT2 methylate DAIIA (**5a**) on the 6-hydroxy group on route to protoemetine as described above but also catalyze 6-*O*-methylation and 7-*O*-methylation of the *R*-epimer DAIA (**5b**) (Fig. 4d–f). An authentic standard of 7-*O*-Me-DAIA was generated through an in vitro Pictet–Spengler reaction of secologanic acid with 4-*O*-Me-dopamine; the identity of 6-*O*-Me-DAIA was established through comparison with this authentic standard and also appears to be identical to a highly accumulating compound in *A.* *salviifolium* (Supplementary Fig. 5). AsDOMT3–7 are selective for the *R*-epimer, DAIA (**5b**), but produce different *O*-methylated product profiles (Fig. 4f). A sequence alignment of these seven OMT enzymes revealed a high level of overall sequence identity (83.7%) (Supplementary Fig. 15) but only 55% identity at the N terminus (Fig. 4g), a region known to confer substrate and product specificity^41^. Indeed, the similarities in substrate and product profiles correlated with the level of identity at the N terminus (Fig. 4f,g). Surprisingly, when all *AsDOMT* genes were expressed together in *N.* *benthamiana*, their product profile differed from the sum profile expected from the expression of single genes; specifically, much higher levels of 6-*O*-Me-DAIA compared to 7-*O*-Me-DAIA were observed, a profile similar to that detected in the native plants (Fig. 4e). It is possible that, upon expression in combination, enzymatic activities influence each other, leading to changed product profiles as previously described^42^.

### A vacuolar exporter enhances protoemetine biosynthesis

**Extended Data Fig. 7:**
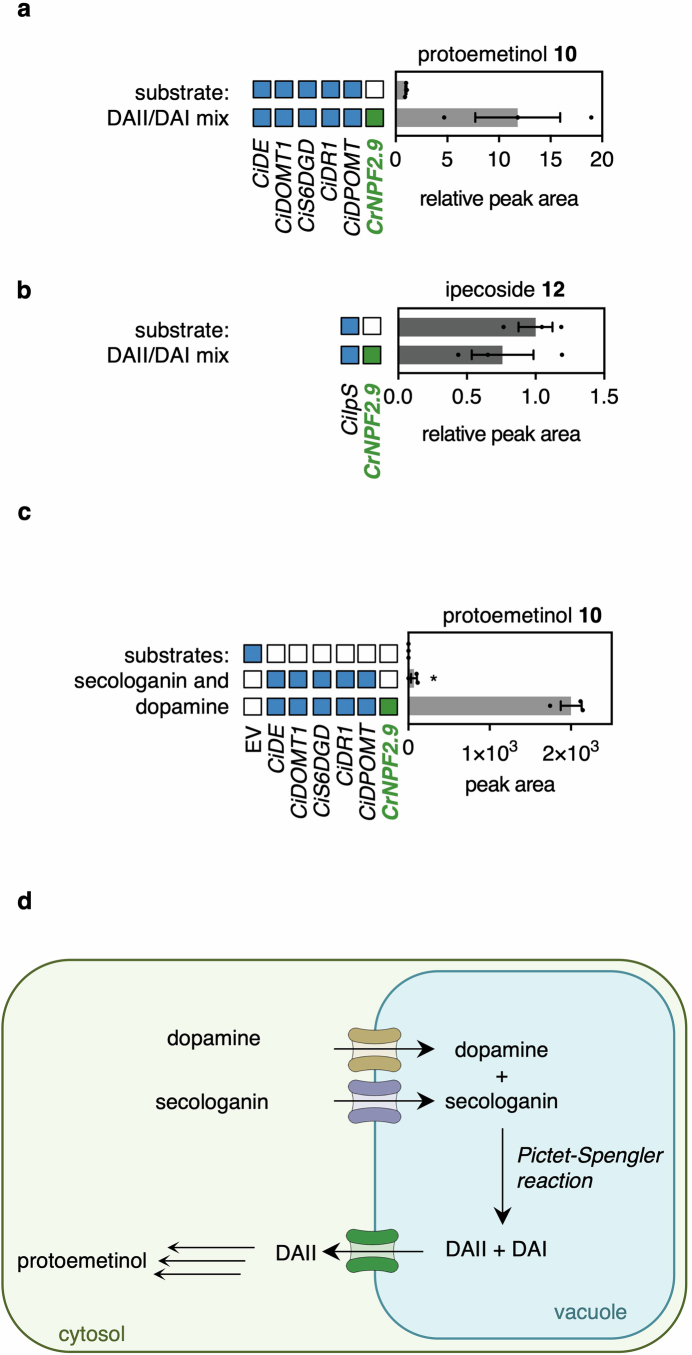
Expression of the *C. roseus* strictosidine exporter gene *CrNPF2.9* boosts *C. ipecacuanha* protoemetine biosynthesis and enables biosynthesis from dopamine and secologanin directly. **a**, comparison of protoemetinol accumulation upon expression of indicated *C. ipecacuanha* biosynthesis genes in *N. benthamiana* with or without co-expression of transporter *CrNPF2.9*. Exogenous DAI/DAII are infiltrated as substrate and allowed to react for 24 hours before the leaf was harvested. Data was extracted from experiment shown in Supplementary Fig. 10. Peak areas are shown here relative to areas from samples where *CrNPF2.9* was not expressed as bars of the mean of three replicates. **b**, expression of *CiIps* with or without co-overexpression of *CrNPF2.9* did not lead to enhanced accumulation of the *R*-epimer derivative ipecoside. **c**, when *C. ipecacuanha* protoemetine pathway genes are expressed and uncoupled secologanin and dopamine are fed for 48 hours, only traces of protoemetinol can be detected (indicated with an asterisk). When *CrNPF2.9* is co-overexpressed protoemetinol is formed to detectable levels. **d**, it is hypothesized that spontaneous *in planta* coupling occurs in the vacuole and the resulting formed DAII is only accessible to the cytosolic pathway enzymes if it is exported by a vacuolar exporter. CrNPF2.9 appears to export DAII. These results suggest the presence of a yet to be identified vacuolar exporter in *C. ipecacuanha* and *A. salviifolium*. LC-MS peak areas are shown as bars of the mean of three biological replicates, error bars are standard error of the mean.

In reconstitution experiments, the majority of infiltrated DAII (**4a**) starting substrate was not converted into downstream products (Extended Data Fig. 4). In the related monoterpene indole alkaloid pathway, the Pictet–Spengler product produced from secologanin and tryptamine is produced enzymatically by a vacuolar enzyme and then exported into the cytosol by a transporter (CrNPF2.9)^43^. We hypothesized that exogenously supplied DAI/I **4b/4a** may be imported into the vacuole by *N.* *benthamiana* transporters^44,45^, rendering these starting materials inaccessible to the downstream cytosolic pathway enzymes. To test whether CrNPF2.9 could export the protoemetine precursor DAII (**4a**), we expressed *CrNPF2.9* with *C.* *ipecacuanha* protoemetine biosynthetic genes in *N.* *benthamiana*. We observed an almost 12-fold relative increase in protoemetinol levels, suggesting that this vacuolar exporter could transport DAII (**4a**) out of the vacuole (Extended Data Figs. 4 and 7a). No increase was observed when *CrNPF2.9* was expressed in combination with *A.* *salviifolium* protoemetine pathway genes (Extended Data Fig. 6), suggesting that this transporter does not recognize DAIIA (**5a**). Expression of *CrNPF2.9* with *CiIpS* did not lead to an increase of the *R*-stereoisomer ipecoside, suggesting that this transporter is specific for the *S*-stereoisomer (Extended Data Fig. 7b). Furthermore, expression of the protoemetine biosynthetic genes with uncoupled secologanin and dopamine (as opposed to DAII **4a**) only yielded protoemetinol when CrNPF2.9 was included (Extended Data Fig. 7c). We hypothesize that the glycosylated secologanin would get imported into the vacuole, where the mildly acidic conditions would facilitate the nonenzymatic formation of DAI/I (Extended Data Fig. 7d). Although we tested numerous candidates, the native vacuolar exporters of *C.* *ipecacuanha* and *A.* *salviifolium* remain to be discovered.

### Nuclear-localized glucosidases deglycosylate *R*-epimers

**Fig. 5:**
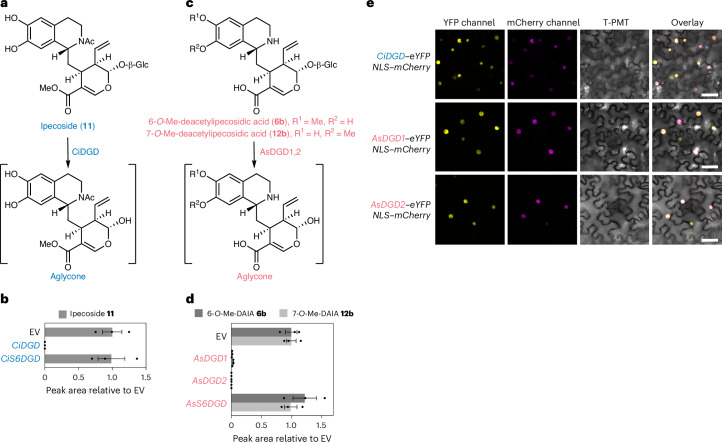
Deglycosylation of *R-*epimer-derived products by nuclear-localized glucosidases. **a**, CiDGD deglycosylates ipecoside. **b**, *N.* *benthamiana* leaf disks expressing *CiDGD* or *CiS6DGD* incubated with ipecoside (**11**) for 24 h. Ipecoside is consumed when *CiDGD* is expressed but not with *CiS6DGD*. Results of incubation with additional substrates are shown in Extended Data Fig. 8a. Bars show the mean peak areas normalized to peak areas in the EV control of three biological replicates; error bars are the s.e.m. and dots are single data points. **c**, AsDGD1 and AsDGD2 deglycosylate *O*-methylated DAIA *R*-epimers. **d**, Incubation of 6-*O*-Me-DAIA (**6b**, light gray, produced in vitro by recombinant AsDOMT3; Methods) or 7-*O*-Me-DAIA (**12b**) (in mix with 7-*O*-Me-DAIIA, **12a**) in *N.* *benthamiana* leaf disks expressing *AsDGD1*, *AsDGD2* or *As6SDGD*. AsDGD1 and AsDGD2 deglycosylate these substrates but AsS6DGD does not. Bars show the mean peak areas normalized to peak areas in the EV control of three biological replicates; error bars are the s.e.m. and dots are single data points. Results of incubation with additional DAIA derivatives are shown in Extended Data Fig. 8b. **e**, Confocal laser scanning microscopy of *N.* *benthamiana* leaves coexpressing C-terminal eYFP fusions of CiDGD, AsDGD1 or AsDGD2 along with mCherry fused to NLS. The data clearly show nuclear localization of these enzymes. Scale bars, 50 µm. Subcellular localization data from two additional biological replicates are shown in Supplementary Fig. 20. Source data

**Extended Data Fig. 8:**
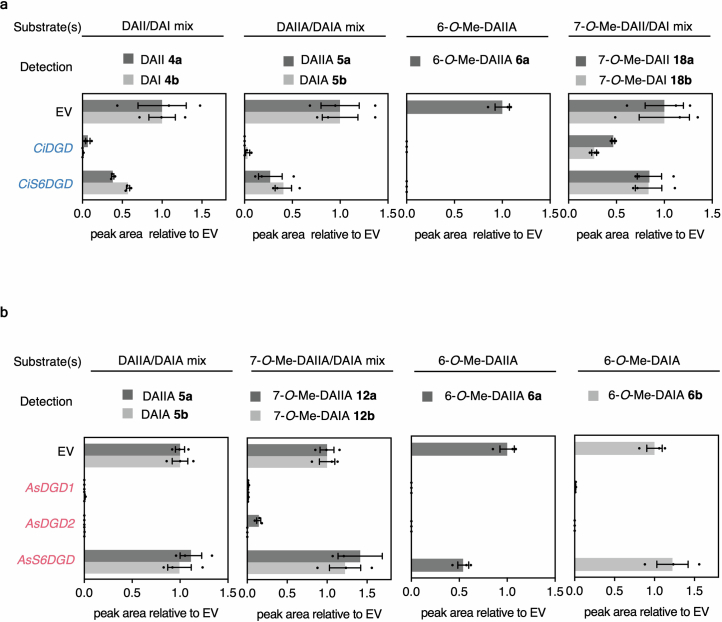
Feeding of ipecac alkaloid glucosides to leaf disks expressing *DGD*s or *S6DGD*s. Consumption of glucosides was measured because the unstable aglycone products cannot be reliably detected. **a**, agroinfiltration of *N. benthamiana* with strains harbouring constructs for overexpression of *CiDGD* or *Ci6SDGD* or empty vector (EV) respectively. Leaf disks were cut and incubated separately with indicated substrates. Note that 7-*O*-Me-DAIA and 7-*O*-Me-DAIIA are not detected in native *C. ipecacuanha* plants. **b**, agroinfiltration of *N. benthamiana* with strains harbouring constructs for overexpression of *AsDGD1* or *2* or *As6S6DGD* or EV respectively. Leaf disks were cut out and incubated each with indicated substrates. AsDGDs deglycosylate all derivatives whereas AsS6DGD only shows activity towards 6-*O*-Me-DAIIA. 6-*O*-Me-DAIIA and 6-*O*-Me-DAIA cannot be obtained readily by Pictet-Spengler chemical reaction and were thus produced *in vitro* by recombinant CiDOMT1 and AsDOMT3, respectively (see methods). Bars are mean peak areas of three biological replicates relative to peak areas measured in EV, error bars are standard error of the mean SEM.

Glucosidase CiDGD deglycosylates the 1*R*-derived stereoisomer ipecoside (**11**)^33^ (Fig. 5a,b). In contrast, CiS6DGD, which we showed to be involved in protoemetine biosynthesis, did not turn over ipecoside (Fig. 5b). Leaf disk assays of CiDGD and Ci6SDGD with substrates DAI/I (**4b**/**4a**), 7-*O*-Me-DAI/I (**18b**/**18a**), DAI/IA (**5b**/**5a**) and 6-*O*-Me-DAIIA (**6a**) suggest that CiDGD has broad substrate specificity, whereas the more selective Ci6SDGD most efficiently deglycosylates 6-*O*-Me-DAIIA **6a**, which is on pathway to protoemetine **8** (Extended Data Fig. 8a). Analogously, the *A.* *salviifolium* glucosidases AsDGD1 and AsDGD2 or AsS6DGD were similarly assayed with the *A.* *salviifolium* substrates DAI/IA **5b**/**5a**, 7-*O*-Me-DAI/IA **12b**/**12a**, 6-*O*-Me-DAIIA **6a** and 6-*O*-Me-DAIA **6b**. AsDGD1 and AsDGD2 consumed 7-*O*-Me-DAIA and 6-*O*-Me-DAIA entirely (Fig. 5d and Extended Data Fig. 8b), while AsS6DGD only consumed the protoemetine pathway intermediate 6-*O*-Me-DAIIA (Extended Data Fig. 8b). In vitro competition assays using recombinant glucosidases were consistent with the conclusion that CS6DGD and AsS6DGD were more selective for the protoemetine pathway intermediate, whereas CiDGD and AsDGD2 had broader substrate specificity (Supplementary Figs. 16–18). Thus, both *C.* *ipecacuanha* and *A.* *salviifolium* seem to have a protoemetine pathway-specific glucosidase along with a glucosidase with broader substrate specificity. The substrate specificity of CiS6DGD and AsS6DGD requires that DAIIA is subjected to 6-*O*-methylation before deglycosylation. In contrast, CiDGD, AsDGD1 and AsDGD2 deglycosylate DAII(A) directly, which prevents formation of protoemetine (Supplementary Fig. 9).

CiDGD, AsDGD1 and AsDGD2 each contain a C-terminal bipartite nuclear localization sequence (NLS) (as predicted with DeepLoc2.0)^46^ (Supplementary Table 1). When these genes were fused to eYFP, each colocalized with an mCherry–NLS marker when expressed in *N.* *benthamiana* leaves^47^ (Fig. 5e and Supplementary Figs. 19 and 20). C-terminal tagged AsDGD1 and AsDGD2 showed localization across the entire nucleus, whereas the N-terminal tagged fusions were localized to a smaller compartment within the nucleus, which could be aggregates, as previously proposed for the glucosidase involved in monoterpene indole alkaloid biosynthesis^48^ (Supplementary Figs. 19 and 20). Taken together, these results indicate that both species derivatize *R*-epimers in a species-dependent manner and contain highly active nuclear-localized glucosidases with relatively broad substrate specificity. The specificity of CiS6DGD and AsS6DGD ensures that 6-*O*-Me-DAIIA rather than DAI/I(A) is deglycosylated by these dedicated protoemetine pathway-specific glucosidases. The protoemetine-specific glucosidase CiS6DGD, which is highly similar to CiDGD, also showed nuclear localization (Supplementary Figs. 19 and 20). The protoemetine-specific glucosidase from *A.* *salviifolium* lacked the NLS and was localized to both the nucleus and cytosol (Supplementary Figs. 19 and 20). In comparison, CiIpS, CiDE and CiDR1 each appeared to be localized to the cytosol (Supplementary Fig. 21).

### Some biosynthetic genes may have evolved independently

**Fig. 6:**
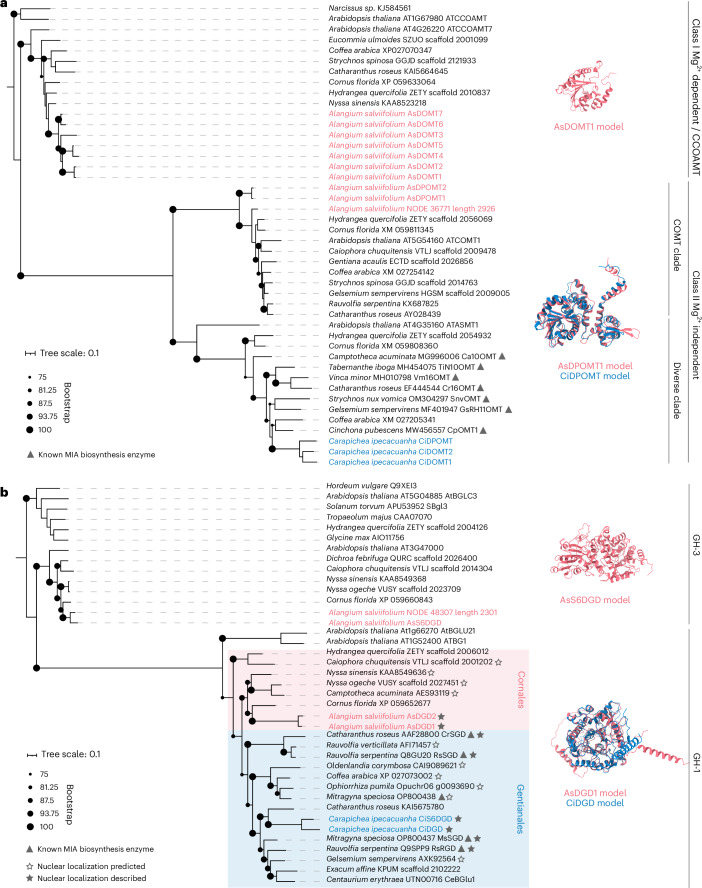
Some ipecac alkaloid biosynthetic enzymes appear to have arisen independently in *C.* *ipecacuanha* and *A.* *salviifolium.* Maximum-likelihood phylogenetic trees of amino acid sequences of pathway enzymes and homologs from other Cornales and Gentianales species. AlphaFold3 models of representative pathway enzymes are shown alongside the different enzyme classes. **a**, OMTs. Analyzed sequences cluster with different known classes and subclades of OMTs. AsDOMTs form part of the clade of caffeoyl CoA 3-OMTs, which are Mg^2+^-dependent class I OMTs well known to be involved in lignin biosynthesis. All other OMTs of this study are class II Mg^2+^-independent class II OMTs. Both classes share a common domain with the same fold but class II OMTs contain an additional domain. Class II OMT sequences form two clades: a clade containing OMTs with high sequence similarity and predicted to have caffeic acid *O*-methylation (COMT) activity and a clade containing OMTs with less sequence similarity and diverse functions (separation of clades has been previously observed^75^). AsDPOMTs are found in the COMT clade, while CiDOMTs and CiDPOMT are part of the diverse clade. A tree containing all bootstrap values is shown in Supplementary Fig. 22. MIA, monoterpendoid indole alkaloids. **b**, Glucosidases. The protoemetine pathway-specific AsS6DGD is a member of the GH-3 family and, thus, has a different protein fold than the other glucosidases characterized in this study, which are of the GH-1 type. The GH-1 *A.* *salviifolium* and *C.* *ipecacuanha* GH-1 sequences cluster with sequences from the Cornales or the Gentianales order, respectively; this suggests parallel evolution. A tree containing all bootstrap values is shown in Supplementary Fig. 23. Enzyme names are shown if available. ATASAMT, *Arabidopsis thaliana* *N*-acetylserotonin OMT; Ca10OMT, *C.* *acuminata* 10-hydroxycamptothecin OMT; TiN10OMT, *Tabernanthe iboga* noribogaine 10-OMT; Vm16OMT, *Vinca minor* 16-hydroxyvincadifformine 16-OMT; Cr16OMT, *C.* *roseus* 16-hydroxytabersonine OMT; SnvOMT, *Strychnos nux-vomica* strychnine OMT; GsRH11OMT, *Gelsemium sempervirens* rankinidine/humatenine-11-OMT; CpOMT1, *C.* *pubescens* quinine OMT; AtBGCL3, *A.* *thaliana* β-glucosidase 3; SBgl3, *Solanum torvum* furastonol glycoside glucosidase; AtBGLU21, *A.* *thaliana* β-glucosidase 21; AtBG1, *A.* *thaliana* β-glucosidase 1; SGD, strictosidine glucosidase; RsRGD, *Rauvolfia serpentina* raucaffricine glucosidase; CeBGlu1, *Centaurium erythraea* β-glucosidase.

**Extended Data Fig. 9:**
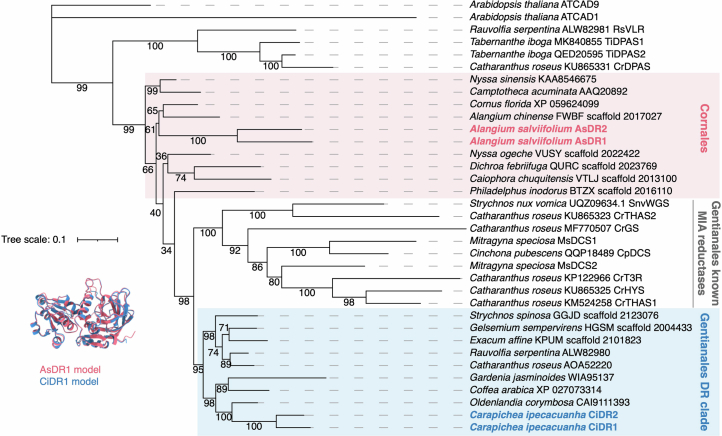
Parallel evolution of *A. salviifolium* versus *C. ipecacuanha* DRs. Maximum likelihood phylogenetic tree of DR amino acid sequences and homologs from other Cornales and Gentianales species. Alphafold3 models indicate same folds for all DRs.

Having elucidated ipecac alkaloid biosynthesis in these two distantly related plants, we performed phylogenetic comparisons of the identified enzymes (Fig. 6, Extended Data Fig. 9 and Supplementary Figs. 22 and 23). These analyses clearly suggest that *A.* *salviifolium* (Cornales) and *C.* *ipecacuanha* (Gentianales) enzymes evolved independently. AsDOMT enzymes belong to the class I Mg^2+^-dependent caffeoyl CoA 3-OMT family^49^ (Fig. 6a). AsDOMT1–AsDOMT7 form a separate subclade, suggesting that the different substrate and product profiles observed among these enzymes (Fig. 4) likely arose through tandem gene duplication and subfunctionalization. All other identified OMT enzymes in ipecac biosynthesis are class II Mg^2+^ independent. These proteins form two well-separated clades; one clade contains AsDPOMTs and the other contains CiDOMTs and CiDPOMT. Parallel, independent evolution is inferred if phylogenetic analysis reveals that two enzymes, despite the same fold and the same enzymatic activity, are found in separate clades of the tree (that is, each protein clusters more closely together with orthologs from their respective phylogenetic groups rather than with the other enzyme in question)^50^. The positions of the *A.* *salviifolium* and *C.* *ipecacuanha* proteins on the phylogenetic tree suggest independent (parallel) evolution. All three *C.* *ipecacuanha* OMTs are closely related, suggesting that these genes arose through tandem duplications and subsequent neofunctionalization to catalyze *O*-methylation of either DAIIA or 10-*O*-demethylprotoemetine.

The protoemetine-specific CiS6DGD belongs to the GH-1 family of glucosidases (Fig. 6b), whereas AsS6DGD is a member of the GH-3 family (Fig. 6b). Thus, CiS6DGD and AsS6DGD, which have different protein folds, represent a case of convergent, independent evolution^2,50^. CiDGD and CiS6DGD are closely related, suggesting that these enzymes evolved through tandem gene duplications and subfunctionalization. The glucosidases that have broad specificity (CiDGD, AsDGD1 and AsDGD2) belong to the same family of glucosidases (GH-1). However, the phylogenetic analysis, which revealed these *A.* *salviifolium* and *C.* *ipecacuanha* proteins to be in separate clades, also suggests parallel, independent evolution. Lastly, the medium-chain alcohol dehydrogenases DRs share the same protein fold but, for the same reasons as above, phylogenetic analysis also suggests that these enzymes evolved independently through means of parallel evolution (Extended Data Fig. 9).

## Discussion

Here, we report the discovery of the protoemetine pathway in two distantly related plants, *C.* *ipecacuanha* and *A.* *salviifolium*. We show that *C.* *ipecacuanha* uses the monoterpenoid precursor secologanin (**1**) while *A.* *salviifolium* uses secologanic acid (**2**). This is consistent with monoterpenoid indole alkaloid biosynthesis in the Gentianales and Cornales clades, where tryptamine is condensed with secologanin or secologanic acid, respectively, to generate strictosidine or strictosidinic acid^21,51^.

Strictosidine (3*S*) is stereoselectively synthesized from secologanin and tryptamine by a well-characterized vacuolar localized Pictet–Spenglerase enzyme (strictosidine synthase)^48^. The 3*R* isomer of strictosidine (vincoside) does not appear to be present in these plants. In contrast, all ipecac alkaloid producing plants contain products derived from both 1*S* and 1*R* stereoisomers of the initial Pictet–Spengler product (DAI **4b** (1*R*) and DAII **4a** (1*S*) in *C.* *ipecacuanha* and DAIA **5b** (1*R*) and DAIIA **5a** (1*S*) in *A.* *salviifolium*). Therefore, these plants must generate both 1*R* and 1*S* Pictet–Spengler products. Nonenzymatic Pictet–Spengler reactions using dopamine have been well established to occur under physiologically relevant conditions; for example, the presence of phosphate can facilitate efficient coupling of dopamine with aldehydes^52^. We observed that nonstereoselective formation of DAI/I/(A) occurs when (1) dopamine and secologanin or secologanic acid are incubated in aqueous buffer at a pH value consistent with the environment of the vacuole; (2) dopamine and secologanin or secologanic acid infiltrate into *N.* *benthamiana* leaves; and (3) dopamine infiltrates into *C.* *roseus* flowers. Analogous experiments using tryptamine instead of dopamine resulted in far lower levels of product, suggesting that the dopamine substrate is highly activated. The known plant Pictet–Spenglerases, strictosidine synthase and norcoclaurine synthase, are localized in the vacuole^36,53^; however, because these enzymes have optimal catalytic efficiency at neutral pH, the acidic environment of the vacuole is not required for the enzymatic reaction^54,55^. However, for a nonenzymatic reaction, the slightly acidic environment of the vacuole could be crucial, even for the highly activated, electron-rich dopamine substrate. We further showed that coexpression of the *C.* *roseus* vacuolar strictosidine exporter CrNPF2.9 with *C.* *ipecacuanha* protoemetine biosynthetic genes greatly enhanced the levels of the protoemetine product and facilitated formation of the protoemetine-derived product from starting substrates secologanin and dopamine (Extended Data Fig. 7). Lastly, we could not detect the presence of enzymatic Pictet–Spengler activity in crude *C.* *ipecacuanha and A.* *salviifolium* extracts (Supplementary Fig. 8). Collectively these observations support the notion that DAI/I(A) formation occurs nonenzymatically and within the plant vacuole in these pathways. However, the involvement of enzymes in these condensation reactions cannot be definitively ruled out.

Both *C.* *ipecacuanha* and *A.* *salviifolium* evolved nearly identical chemistry to generate the ipecac alkaloid protoemetine. Secologanic acid is an iridoid, a class of natural product that is ancestral to all Asterids^56^. Iridoid pathway genes from *C.* *acuminata* and *C.* *roseus* belonging to the phylogenetically distant asterid lineages Cornales and Gentianales, respectively, have been shown to be orthologs with high levels of sequence identities^21,22,57^. Thus, the biosynthesis of the secologanic acid precursor is likely also conserved between *A.* *salviifolium* (Cornales) and *C.* *ipecacuanha* (Gentianales). The addition of a methyl ester to secologanic acid is a chemical innovation that is not present in *A.* *salviifolium*, consistent with other members of Cornales^21,51^. Analogously, the esterase (CiDE) that eventually removes this methyl ester is present only in *C.* *ipecacuanha*.

After the Pictet–Spengler reaction that condenses secologanin or secologanic acid with dopamine, CiDOMT (*C.* *ipecacuanha*, class II OMT) and AsDOMT (*A.* *salviifolium*, class I OMT) methylate DAII(A). CiDOMT and AsDOMT both methylate DAIIA but are phylogenetically distant and share only partially the same structural fold (Fig. 6a). Thus, these proteins most likely represent an example of parallel evolution, using the terminology established by Weng and Noel^50^. Parallel evolution refers to an event in which ancestor enzymes with a shared structural lineage have independently evolved to have the same biochemical activity. In the next pathway step, CiS6DGD and AsS6DGD, both proposed to deglucosylate **6a** in protoemetine biosynthesis, are members of different structural classes of glucosidase (GH-1 versus GH-3) (Fig. 6b). Using the terminology established by Weng and Noel^50^, two enzymes with distinct folds catalyzing the same reactions would be an example of convergent evolution. Then, both *C.* *ipecacuanha* and *A.* *salviifolium* use a medium-chain alcohol dehydrogenase to catalyze reduction of this deglycosylated product. However, their distant positions on the corresponding phylogenetic tree (Extended Data Fig. 9) suggest that these reductases have evolved independently through parallel evolution. The reduced product is finally methylated by CiDPOMT (*C.* *ipecacuanha*) and AsDPOMT (*A.* *salviifolium*), which are both class II OMTs. Phylogenetic analysis also suggests that these CiDPOMT and AsDPOMT evolved through parallel evolution because these proteins are found in separate clades in the phylogenetic tree (Fig. 6a). Collectively, phylogenetic analysis suggests that the four pathway steps from DAII(A) to protoemetine appear to be examples of either convergent or parallel evolution. Our phylogenetic analyses that support a model of independent evolution are consistent with analyses from other examples of independent evolution in plant specialized metabolism, such as glucosinolates^3^, benzoxazinoids^4^, caffeine^5–7^, cannabinoids^8^ and cardenolides^9–11^.

Although protoemetine biosynthesis indicates the same chemical outcome, there appear to be striking species-specific evolutionary strategies by which enzymes were recruited to these two protoemetine pathways. In *C.* *ipecacuanha*, all involved OMTs (CiDOMT1, CiDOMT2 and CiDPOMT) are closely related and likely arose through gene duplication and subsequent neofunctionalization. This would exemplify the ‘forward hypothesis’ of evolution, in which a pathway evolves from the first biosynthetic enzyme onward through means of tandem gene duplication and neofunctionalization^58,59^. Conversely, in *A.* *salviifolium*, enzymes from different OMT classes appear to have been independently recruited to ipecac alkaloid biosynthesis (AsDOMT is a class I OMT; AsDPOMT is a class II OMT). This would exemplify the ‘patchwork hypothesis’ of evolution, in which distinct ancestral enzymes are independently recruited to a pathway.

Analogously, *C.* *ipecacuanha* and *A.* *salviifolium* each evolved glucosidases with broad substrate specificity (DGDs) and glucosidases that appear to be specific to the intermediate on pathway to protoemetine biosynthesis (S6DGDs). While CiDGD and AsDGD are GH-1 glucosidases, CiS6DGD and AsS6DGD are GH-1 and GH-3 glucosidases, respectively. Thus, in *C.* *ipecacuanha*, CiDGD and CiS6DGD likely evolved through tandem gene duplication and subfunctionalization (forward hypothesis), whereas, in *A.* *salviifolium*, AsS6DGD convergently evolved this function upon recruitment from the GH-3 family, a group of glucosidases with a different fold not commonly associated with specialized metabolism^60,61^ but rather with cell wall biosynthesis (patchwork hypothesis)^62–65^.

Within Gentianales, the downstream steps of the ipecac alkaloid pathway appear to have evolved independently from the related monoterpene indole alkaloid pathway enzymes. Although some of the biosynthetic steps of these two pathways have similar chemistry (for example, the reductase DCS from *Cinchona* spp.^38^ and the OMT from vinblastine biosynthesis^66^) the enzymes that catalyze the respective steps (DR and OMT) do not cluster together with known monoterpene indole alkaloid biosynthesis enzymes but instead form sister clades (Fig. 6a and Extended Data Fig. 9).

While both *C.* *ipecacuanha* and *A.* *salviifolium* convert the *S-*epimer to protoemetine, the *R-*epimers are derivatized in a chemically different and species-dependent manner through a simple and shorter ‘shunt’ pathway. It is likely that the *R*-epimers ipecoside (*C.* *ipecacuanha*), 6-*O*-Me-DAIA and 7-*O*-Me-DAIA (*A.* *salviifolium*) accumulate in the vacuole because this is the typical storage location in plants for glycosylated specialized metabolites^67^. These products would, therefore, be separated from the nuclear-localized glucosidases (DGDs) that act upon these substrates. Deglycosylation of ipecoside and 6/7-*O*-Me-DAIA leads to a dialdehyde moiety that is highly reactive^48,68,69^. The *R*-epimer-derived ipecoside, 6-*O*-Me-DAIA and 7-*O*-Me-DAIA could be released from the vacuole upon tissue damage, only then coming into contact with the nuclear-localized glucosidases to generate a reactive and, therefore, toxic defense molecule, as previously hypothesized for ipecoside^33^. Despite the species-specific chemical derivatization (*O*-methylation versus acetylation) the *R*-epimers may serve similar ecological functions as defensive agents. Mechanisms in which the enzyme is spatially separated from its substrates to avoid the constant accumulation of potentially toxic compounds were first described for glucosinolates^70,71^. Instances in which a glucosidase is located in the nucleus while the substrate is stored in the vacuole have also been described for monoterpenoid indole alkaloids, saponins and secoiridoids^48,72,73^. Specialized metabolites stored in inactive form that are activated upon tissue damage have recently been referred to as phytoavengins^74^. Overall, this comparative pathway elucidation of ipecac alkaloid biosynthesis highlights the diversity in evolutionary strategies to evolve chemically complex molecules and provides a foundation for metabolic engineering of these biologically active molecules.

## Methods

### Plant material and sampling

*C.* *ipecacuanha* plantlets were grown in vitro on propagation medium (1× Murashige and Skoog (MS) medium including vitamins, 3% sucrose, 3 mg L^−1^ 6-benzylaminopurine, 10 µg L^−1^ 1-naphthaleneacetic acid and 8 g L^−1^ agar, pH 5.7). Upon arrival, plantlets were transferred to root induction medium (0.75× MS medium including vitamins, 3% sucrose, 0.5 mg L^−1^ 1-naphthaleneacetic acid and 8 g L^−1^ agar, pH 5.7). After approximately 6 weeks, roots formed and the regenerated plants were transferred to soil. For tissue-specific metabolite profiling and RNA-seq, plants were harvested 4 months after transfer to soil. *A.* *salviifolium* plants were received as cuttings from the Botanical Garden of Ghent University and rooted on rockwool. After 6 weeks, rooted plants were transferred to soil. For tissue-specific profiling, plants were harvested 14 months after transfer to soil. Both species were grown in a temperature-controlled and light-controlled greenhouse with the following conditions: 12-h light–dark cycle at 28–30 °C and 24–26 °C, respectively, and 70–80% humidity. For tissue-specific analyses, three plants from each species were macrodissected as shown in Supplementary Fig. 1. Tissues were immediately flash-frozen and ground in liquid nitrogen using an IKA A11 basic analytical mill or mortar and pestle. The frozen finely ground powder was kept at −80 °C until further processing.

### Metabolite extraction for metabolite profiling

Finely ground material was extracted with 100% methanol supplemented with 10 mg L^−1^ caffeine as internal standard. The volume of methanol was normalized to fresh tissue weight (1:10 mg:µl). Samples were vortexed for 1 min and sonicated for 5 min at room temperature. After 1 h of incubation at room temperature, samples were centrifuged for 15 min at 18,000*g* and supernatants were filtered through Fisher PTFE syringe filters (0.22 μm). Filtered extracts were diluted in 80% methanol containing 0.1% formic acid and 1:10 and 1:50 dilutions were analyzed by untargeted ultrahigh-performance liquid chromatography–mass spectrometry (UPLC–MS). *C.* *ipecacuanha* samples were analyzed using UPLC–MS method 1 and *A.* *salviifolium* samples were analyzed using UPLC–MS method 2. For comparable traces shown in Fig. 2, representative samples of each plant were analyzed in parallel with UPLC–MS method 3.

### UPLC–MS/MS methods

Method 1 was used for *C.* *ipecacuanha* tissue-specific metabolite profiling. An Elute LC system (Bruker Daltonics) was coupled to an Impact II high-resolution quadrupole time-of-flight MS instrument (Bruker Daltonics). An Acquity UPLC BEH C18 (2.1 × 50 mm, 1.7 μm; 130 Å) column (Waters) was set at 40 °C and 0.6 ml min^−1^ flow rate and 2 µl of samples were injected. The mobile phase was A:B where A was water with 0.1% formic acid and B acetonitrile with 0.1% formic acid. The gradient was as follows: 5% B at 1 min to 8% B at 3 min, to 13% B at 5 min and to 30% at 8 min. Then the column was flushed at 100% B until 9.8 min and re-equilibrated to 5% B until 12 min. Method 2 was used for *A.* *salviifolium* tissue-specific profiling and was identical to method 1 with the exception of the column temperature, which was set to 35 °C. Method 3, used for all other experiments, was identical to method 2 but used an UltiMate 3000 UPLC system (Thermo Fisher Scientific). For all methods, ionization was performed using pneumatic-assisted electrospray ionization (ESI^+^) with 4,500 V (methods 1 and 2) or 3,500 V (method 3) of capillary voltage, a 500-V end-plate offset and a nebulizer pressure of 2.5 bar, with nitrogen at 250 °C and a flow of 11 L min^−1^ as the drying gas. Acquisition was performed at 12 Hz following a mass range from 80 to 1,000 *m/z* with data-dependent MS/MS, an active exclusion window of 0.2 min and a reconsideration threshold of 1.8-fold. Fragmentation was triggered on an absolute threshold of 400 and limited to a total cycle time range of 0.5 s. For collision energy, the stepping option model (from 20 to 50 eV) was used. At the start of each run, the *m/z* values were recalibrated using the expected cluster ion *m/z* values of a direct source infusion of sodium formate–isopropanol solution. To prevent the contamination of the MS by injection peaks and salt, the first minute of each run was run isocratically at 5% B and redirected to waste.

### MS data analysis

Data was converted to mzml or mzxml format and imported to MZmine (versions 3.6.0 and 4.1.0)^76^. Extracted ion chromatogram (EIC) traces and MS^2^ data of compounds of interest were exported from MZmine. Peak areas were calculated using the MZmine processing wizard and exported. Peak areas of compounds of interest were normalized to the internal standard caffeine and converted to intensity per second. Further data analysis and construction of graphs were performed in GraphPad Prism version 10.2.3 and 10.3.0 for Mac OS X. For tentative identification of molecular structures and analysis of MS^2^ fragmentation pattern, SIRIUS version 4 was used^77–79^. Chemical structures were drawn in ChemDraw Professional 20.1.0.112.

### Nonenzymatic Pictet–Spengler reaction

For coupling in *N.* *benthamiana*, a solution of 1 mM dopamine hydrochloride **3** or 1 mM tryptamine hydrochloride and 1 mM secologanin **1** or secologanic acid **2**, respectively, was infiltrated into leaves of 4-week-old plants (grown as described below). A leaf sample was taken immediately after infiltration for a control and snap-frozen. For coupling in *C.* *roseus*, petals of freshly opened flowers of 4-month-old plants (grown in a controlled growth chamber at 16 and 8 h of light and dark, 23 °C, 40–50% humidity) were infiltrated with 1 mM dopamine hydrochloride **3** or 1 mM tryptamine-*d*_5_ hydrochloride solution. Samples were harvested after 24 h. Control samples were taken after 24 h from flower petals infiltrated with water. Metabolite extraction and analysis were performed as described below for *N.* *benthamiana*.

### Enzyme activity tests on native plant protein extracts

Protein extraction and activity assays were performed on the basis of previously described methods^18,25^. Young leaves of *A.* *salviifolium* or *C.* *ipecacuanha* were freshly harvested, snap-frozen, ground in liquid nitrogen with a mortar and pestle and immediately extracted in 2 ml of ice-cold protein extraction buffer (100 mM Tricine-HCl pH 7.5, 10 mM β-mercaptoethanol and 3 mM EDTA) per 1 g fresh weight (FW). Extraction took place by gentle shaking at 4 °C for 30 min. Extracts were then centrifuged at 18,000*g* and 4 °C for 15 min and the supernatants were desalted into 100 mM Tricine-HCl pH 7.5 using Zeba spin desalting columns with a 7-kDa molecular weight cutoff (Thermo Scientific) according to the manufacturer’s instructions. Desalted extracts were then immediately used in activity assays. A part of each protein extract was heated for 10 min at 98 °C and centrifuged at 18,000*g* for 25 min; the supernatant served as the boiled enzyme control. Activity assays were performed in triplicates from the same desalted protein extracts. Assays (200 µl of total volume per replicate) contained 1 mM δ-gluconolactone, 1 mM dopamine hydrochloride **3**, 1 mM secologanin **1** (*C.* *ipecacuanha* assay) or secologanic acid **2** (*A.* *salviifolium* assay) and desalted protein extracts or boiled extracts as negative controls. Assays to detect OMT activity in *A.* *salviifolium* contained 1 mM δ-gluconolactone, 1 mM mix of DAIIA **5a** and DAIA **5b** (corresponding to ~500 µM per epimer), 1 mM MgCl_2_ and 1 mM *S*-(5′-adenosyl)-l-methionine (SAM) chloride dihydrochloride. Assays to detect esterase activity in *C.* *ipecacuanha* extracts contained 1 mM δ-gluconolactone and 1 mM mix of DAII **4a** and DAI **4b** mix (corresponding to ~500 µM per epimer). Assays were pipetted on ice and then incubated with gentle shaking at 30 °C. At indicated time points, aliquots (10 µl) were removed and immediately flash-frozen in liquid nitrogen (time point zero was taken immediately upon pipetting and before transfer to the incubator). For metabolite analysis, 90 µl of 60% methanol containing 0.1% formic acid and 1 mg L^−1^ caffeine as the internal standard was added to each aliquot and precipitants were pelleted by centrifugation at 18,000*g* at room temperature for 30 min. Supernatants were analyzed with UPLC–MS method 3.

### RNA extraction and RNA-seq

Total RNA was extracted using the RNeasy Plant Mini Kit (Qiagen) from replicate 1 of the same tissue material that was used for metabolite profiling according to the manufacturer’s instructions, including on-column DNAse digest. RNA concentrations and purity were determined with a Nanophotometer N60 (Implen). Samples of sufficient concentration and purity were shipped to Novogene where mRNA library preparation and sequencing were performed according to the company’s standard protocol for mRNA-seq. RNA integrity and quantitation were assessed using the RNA Nano 6000 assay kit of the Bioanalyzer 2100 system (Agilent Technologies). All samples were above the required minimum RNA integrity number. Sequencing was performed on an Illumina NovaSeq 6000 PE150 platform with a data output target of 9 GB of raw data. Adaptor cleaved raw data were provided as FASTQ files.

### Transcriptome analysis and candidate selection

All data processing was performed in-house. Reads were quality-checked with FastQC and trimmed using Trimmomatic^80^. Transcriptomes were assembled by rnaSPAdes using the trimmed reads of all tissues combined as an input^81^ with default settings, except *k*-mer size was adjusted to 55, 77 and 99 to discriminate isoforms with high sequence identity. The resulting assemblies were assessed with Busco for the Eudicots lineage and were 95.6% complete for *C.* *ipecacuanha* and 97.2% complete for *A.* *salviifolium*^82^. FastQC (Galaxy version 0.73), Trimmomatic (Galaxy version 0.38.1) and rnaSPAdes (Galaxy version 3.15.4) were all run on an in-house Galaxy server^83^. Functional annotation of transcripts was performed on OmicsBox (Biobam) using the SwissProt 2021 database (basic local alignment search tool (BLAST) parameters: *E* value, 1.0 × 10^−3^; number of hits, 10; word size, 6; low-complexity filter, on; number of threads, 40; high-scoring pair length cutoff, 33) and eggNOG-mapper^84,85^. Reads were mapped to transcriptomes using CLC Genomics workbench 21.0.4 (Qiagen) with the following parameters: mismatch cost, 2; insertion cost, 3; deletion cost, 3; length fraction, 0.8; similarity fraction 0.85; autodetect paired distances, on; maximum number of hits for a read, 10. Counts per million (CPM) normalized by the trimmed mean of M values were used for downstream analyses.

To identify known *C.* *ipecacuanha* pathway genes, the published sequences (National Center for Biotechnology Information (NCBI) AB455576, AB527082, AB527083, AB527084 and AB576187) were BLASTed (BLASTn) against our transcriptome^32–34^. The level of pairwise sequence identity at the nucleotide level between the published sequences and the amplified sequences was 96.8% for *CiDGD* (previously called *IpeGlu1*), 97.2% for *CiDOMT1* (previously called *IpeOMT1*), 98.7% for *CiDOMT2* (previously *IpeOMT2*) and 98.5% for *CiDPOMT* (previously *IpeOMT3)*. The closest homologous sequence to another published OMT^34^ was also *CiDOMT2* (98.8% pairwise identity). These observed small differences in sequence identity between published and newly cloned sequences can be attributed to single-nucleotide polymorphisms of different plant sources. The expression profile of *CiDOMT1* was used as bait for Pearson correlation (Extended Data Fig. 2).

To identify candidates for the missing esterase and reductase, *C.* *pubescens CpDCE* (MW456556.1) and *CpDCS* (MW456554.1)^38^ were BLASTed (tBLASTn) against the transcriptome, which resulted in 9 contigs with 38–44% identity and 15 contigs with 55.4–62% identity, respectively. The BLAST hits were then filtered for high correlation with the *CiDOMT1* tissue-specific expression profile (Pearson correlation > 0.85) and high absolute expression level in young leaf and rhizome (>50 CPM). Further mining the list of coexpressed and highly expressed contigs for relevant functional annotations revealed *Ci6SDGD* and *CiIpS*. For *A.* *salviifolium*, no pathway genes were previously published and BLASTing *CiDOMTs* against the transcriptome did not yield any orthologs with high identity. We, therefore, identified a *TyrDC* ortholog whose expression pattern matched the tissue-specific metabolite profiling and used it as bait for coexpression analysis. Among the highly coexpressed candidates (Pearson correlation > 0.75, CPM in leaf buds and/or roots > 50), we selected those with functional annotations consistent with OMTs, dehydrogenases and glycosyl hydrolases for screening. After positive screening results for *AsDOMT*s, three additional highly homologous sequences with higher root-specific expression (*AsDOMT2*, *AsDOMT6* and *AsDOMT7*) were included in pathway reconstitution experiments.

### Gene cloning

Complementary DNA (cDNA) was prepared from total RNA of *A.* *salviifolium* leaf buds and roots and *C.* *ipecacuanha* young leaves and rhizome (extracted as described above) using the RevertAid first-strand cDNA synthesis kit (Thermo Scientific) according to manufacturer’s instructions. *AsDOMT5* and *AsDPOMT2* could not be amplified and were, therefore, obtained as synthetic sequences from Twist Biosciences. Coding sequences were amplified with the Q5 high-fidelity 2× master mix (New England Biolabs) using cDNA or synthetic genes as templates and gene-specific primers containing overhangs for In-Phusion cloning (Supplementary Table 5). Amplified sequences were gel-purified using the Zymoclean gel DNA recovery kit (Zymo Research) and cloned using the 5× In-Fusion snap assembly master mix (TaKaRa Bio). For expression in *N.* *benthamiana*, coding regions were inserted into a modified 3Ω1 vector (containing *UBQ10* promoter and terminator from *Solanum lycopersicum*^86^) previously digested with BsaI-HF v2 (New England Biolabs). For expression in *Escherichia coli*, coding sequences were cloned into pOPINF^87^ previously digested with KpnI-HF and HindIII-HF (New England Biolabs). Heat-shock-competent *E.* *coli* TOP10 cells were transformed and grown overnight in a 37 °C incubator on Luria–Bertani agar plates containing the respective antibiotics. Plasmids were isolated from overnight cultures of single colonies using the Wizard Plus SV Miniprep DNA purification system kit (Promega). Inserted sequences were confirmed by Sanger sequencing.

Sequences for subcellular localization were amplified and purified as described above but using the previously cloned 3Ω1 constructs as PCR templates (described above) and gene-specific primers with overhangs compatible with Golden Gate cloning for N-terminal or C-terminal eYFP or mCerulean3 fusion proteins (Supplementary Table 5). Constructs (level 1) were assembled using BsaI (New England Biolabs), T4 DNA ligase (New England Biolabs), the pDGB3_ɑ1 vector^88^, pUPD_pSIUbq10 and pUPD_TeSlUbq10^86,89^ and the gel-purified PCR products*. As6SDGD* and *CiIpS* contained a BsaI restriction site that was removed by introducing a silent mutation in an overlapping PCR before assembly. Assembly consisted of 50 cycles of 5 min at 37 °C followed by 5 min at 16 °C and was stopped by incubation at 65 °C for 10 min. Additionally, *CrNPF2.9* was cloned through the same Golden Gate cloning procedure described above into pDGB3_ɑ1 (without eYFP tag), whereas the silencing repressor gene *p19* was obtained as pUPD_p19 (Addgene, GB0038) and then cloned as above into pDGB3_ɑ1.

### Transient expression in *N.benthamiana*

In all cases, the *Agrobacterium tumefaciens* GV3101 strain was used and cultured at 28 °C in YEB medium containing rifampicin and gentamycin and the appropriate antibiotic for plasmid selection. *N.* *benthamiana* plants used for infiltration were 3–4 weeks old (grown in a greenhouse with 16 and 8 h of light and dark at 23–26 °C and 16–22 °C, respectively, and 40–70% humidity). Cells were transformed through electroporation, recovered in YEB without antibiotics and incubated for 2 days on YEB plates containing antibiotics. Single colonies were confirmed by colony PCR and grown in liquid YEB for 24 h. From these cultures, glycerol stocks were prepared and stored at −80 °C. For agroinfiltration, cells were grown on YEB plates similar to a previously described method with modifications^90^. Cells from glycerol stocks were spread on YEB plates containing antibiotics and 100 µM acetosyringone and grown for 24 h until a visible layer of bacteria appeared. The bacteria were transferred to 1–2 ml of infiltration medium (10 mM MES, 10 mM MgCl_2_ and 100 μM acetosyringone, pH 5.7) and gently resuspended. The optical density at 600 nm (OD_600_) was measured in 1:10 dilutions using an Implen OD600 DiluPhotometer.

For all pathway reconstitution experiments, strains were mixed and diluted in infiltration buffer to OD_600_ = 0.1 per strain. A strain harboring a construct with the *p19* gene was coinfiltrated in all cases. The culture mixtures were infiltrated into *N.* *benthamiana* leaves and plants were kept for 16 h in the dark and subsequently grown under grow light (16 and 8 h of light and dark). Replicates were from three individual plants. Indicated substrates were infiltrated as 500 µM aqueous solutions of epimeric mixtures (described below) 3 days after infiltration. Leaf material was harvested 24 h after substrate infiltration by flash-freezing in tubes containing metal beads. In the case of infiltration of uncoupled secologanin and dopamine substrates, leaf material was harvested 48 h after substrate infiltration to allow time for coupling.

For analysis of *N.* *benthamiana* leaves by confocal laser scanning microscopy, *A.* *tumefaciens* culturing and infiltration was performed as above but at OD_600_ = 0.1–0.3 per strain. To confirm subcellular localization, each strain harboring an eYFP or mCerulean3 fusion construct was coinfiltrated with a strain harboring a construct with free mCherry or NLS–mCherry as fluorescent markers for cytosol or nuclear localization, respectively^47^, along with a strain for expression of *p19*. Leaf tissue was analyzed 2–3 days after infiltration.

### Leaf disk assays

To test glucosidase activity toward different substrates, a leaf disk assay was used as previously described with modifications^91–93^. Growth and infiltration of *A.* *tumefaciens* were performed as described above and replicates were from three individual plants. Then, 3 days after agroinfiltration, 1-cm leaf disks were cut using a leaf puncher and incubated with 200 µl of substrate solution in 50 mM HEPES buffer pH 7.5 in 48-well plates. Substrate solutions were prepared as master mixes so that concentrations were identical in each well. Substrate concentrations were 400 µM total, corresponding to 200 µM per epimer for chemically produced epimeric substrate mixtures DA(I)I, DAI(I)A, 7-*O*-Me-DAI(I) and 7-*O*-Me-DAI(I)A. Commercially available ipecoside was used at 200 µM. The reactions of enzymatically produced substrates (see below) were monitored by UPLC–MS and concentrations were estimated at 250 µM. Plates were sealed with parafilm to avoid evaporation and incubated for 24 h under growth lights (16 and 8 h of light and dark).

### Metabolite extraction from *N.benthamiana*

Leaf material was ground using two 4-mm metal beads and a TissueLyser (Qiagen) with precooled adaptors and extracted with 100% methanol containing 0.1% formic acid and 2 mg L^−1^ caffeine as the internal standard. For substrate infiltrated leaves, 150 µl per 100 mg leaf material was used. In the case of leaf disk assays, 50 µl was used per single leaf disk. Samples were sonicated for 10 min, incubated on a rotator for 20 min and centrifuged at 18,000*g* for 15 min. The supernatants were mixed 1:2 with MQ H_2_O to improve shape of early eluting peaks and filtered through a 0.45-µm low-binding hydrophilic PTFE filter plate (MultiScreen Solvinert 96, Merck-Millipore) into a 96-well microtiter plate (SureSTART WebSeal, Thermo Scientific) according to the manufacturer’s instructions. Plates were sealed with Rapid Slit Seal (BioChromato) and immediately analyzed with UPLC–MS method 3.

### Confocal laser scanning microscopy

For confocal laser scanning microscopy, the leaf disks were put on a glass slide, mounted with water and covered with a coverslip. Fluorescence was observed and imaged with a W Plan-Apochromat ×40 1.0 differential interference contrast M27 water objective on a cLSM 880 (both Zeiss) equipped with two lasers for excitation of the two different fluorophores. mCherry was excited at 543 nm with a helium–neon laser and emission was filtered between 600 and 651 nm. eYFP was excited at 514 nm with an argon laser and emission was filtered between 525 and 561 nm. mCerulean3 was excited at 458 nm with an argon laser and emission was filtered between 466 and 490 nm. The micrographs with eYFP were taken sequentially in two tracks for each image. In the first track, mCherry emissions were captured along with the transmitted light image. In the second track, eYFP emission was captured. ZEN black 2.1 V.14.0.18.201 was used as software (Zeiss). Images were adjusted and processed with ImageJ software^94^.

### Recombinant protein production and purification

Expression and purification of AsDOMT3, CiDOMT1 and CiDE were performed as previously described with modifications^95^. Briefly, *E.* *coli* SoluBL2(DE3) cells were transformed by heat shock with pOPINF constructs. Precultures were inoculated from single colonies, grown overnight at 37 °C and used to inoculate 100 ml of 2× YT medium. Cultures were grown at 37 °C until an OD_600_ of 0.5–0.6, cooled to room temperature and induced with 0.2 mM IPTG. After induction, the cultures were grown at 18 °C overnight and harvested by centrifugation the next day. Cell pellets were lysed using B-PER complete bacterial protein extraction reagent (Thermo Scientific) supplemented with EDTA and subsequently centrifuged according to the manufacturer’s instructions. Supernatants were incubated with gentle shaking in Falcon tubes with 250 µl of Ni-NTA agarose (Qiagen) for 1 h at 4 °C to allow binding of His-tagged proteins. Slurry was pelleted gently by centrifugation at 1,000*g* for 30 s. The supernatant was removed and the slurry was washed three times with ice-cold wash buffer (50 mM Tris-HCl pH 8, 50 mM glycine, 5% glycerol, 500 mM NaCl and 20 mM imidazole) by inversion, centrifugation and removal of supernatant. Proteins were eluted using elution buffer (as wash buffer but containing 500 mM imidazole). Elution fractions were concentrated and buffer was exchanged to storage buffer (20 mM HEPES and 150 mM NaCl, pH 7.5) using Amicon ultracentrifugal filters (Millipore) with the appropriate exclusion size according to the manufacturer’s instructions. Purity was assessed through SDS–PAGE and concentration was determined by using the extinction coefficient and measuring the absorbance at 280 nm. Proteins were flash-frozen in small aliquots in liquid nitrogen and stored at −70 °C for storage. Expression and purification of AsDGD2, AsS6DGD, CiDGD and CiS6DGD were performed as above with some modifications. To increase yield, expression took place in larger cultures (up to 1 L). Cell pellets were lysed on ice for 30 min with approximately 6 ml of lysis buffer per 1 g of cell pellet (50 mM Tris-HCl pH 8, 50 mM glycine, 5% glycerol, 500 mM NaCl, 20 mM imidazole, 0.2 mg ml^−1^ lysozyme and one tablet (per 50 ml buffer) of complete EDTA free protease inhibitor (Roche)) and sonicated for 2.5 min (2 s on, 3 s off) on ice (Bandelin UW 2070). Subsequent purification and concentration steps were performed as described above.

### Phylogenetic analyses

Sequences were obtained through BLAST searches against the publicly available NCBI and 1KP^96^ databases or from *C.* *ipecacuanha* and *A.* *salviifolium* transcriptomes generated in the frame of this study. Sequences and accession numbers are provided in Supplementary Dataset 1. Full-length amino acid sequences were aligned with webPRANK (https://www.ebi.ac.uk/goldman-srv/webprank/)^97^ and maximum-likelihood phylogenetic trees were built using the IQ-TREE web server (http://iqtree.cibiv.univie.ac.at/)^98^ (automatic substitution model; bootstrap value = 1,000). iTOL was used to visualize and graphically edit trees (https://itol.embl.de/)^99^.

### Protein models

Protein models were predicted with AlphaFold3 through the AlphaFold Server (https://alphafoldserver.com/)^100^. Models were visualized using ChimeraX version 1.8 for Mac^101^.

### Commercially available chemicals and standards

Secologanin (50741), dopamine hydrochloride (H3502), tryptamine hydrochloride (246557), 4-*O*-Me-dopamine hydrochloride (H3132), cephaeline dihydrochloride (PHL85887, phyproof reference substance), emetine dihydrochloride (PHL89489, phyproof reference substance) and ipecoside (TA9H93CFC242, TargetMol) were purchased from Sigma.

### Chemically produced standards and substrates

Secologanic acid was produced through alkaline hydrolysis of secologanin by incubating secologanin with 0.1 M NaOH (40 µl per 1 mg secologanin) for 5 h, similarly to a previously described protocol^102^. The solution was neutralized and completeness of the reaction was confirmed through LC–MS analysis. Secologanic acid was stored in solution at −25 °C.

Isotopically labeled tryptamine-*d*_5_ was obtained as previously described^103^.

Epimeric mixtures of DAI(I), DAI(I)A, 7-*O*-Me-DA(I)I and 7-*O*-Me-DAI(I)A were obtained using a previously described protocol with modifications^33^. Briefly, 10 mM secologanin or secologanic acid was incubated together with 10 mM dopamine hydrochloride or 4-*O*-Me-dopamine hydrochloride, respectively, in 0.1 M citrate and 0.2 M phosphate buffer pH 5.5 for 24 h at room temperature. This was sufficient for coupling as confirmed by UPLC–MS analysis. 6-*O*-Me-DAI(I)A cannot be produced using this protocol and was produced enzymatically (described below). For in vitro competition assays of glucosidases (described below) DAI(I)A and 7-*O*-Me-DAI(I)A were produced using 20 mM of each substrate and DAI(I) and 7-*O*-Me-DAI(I) were produced using 50 mM of each substrate.

Protoemetinol was produced from protoemetine (described below) by reduction with NaBH_4_. An aliquot of 50 µg of protoemetine was incubated in methanol with 0.5 g L^−1^ NaBH_4_ for 30 min at room temperature. The reaction was quenched by adding an equal volume of acetone followed by a 15-min incubation at room temperature. The reaction was centrifuged at 18,000*g* for 15 min, diluted with methanol and analyzed with UPLC–MS method 3, which indicated full conversion to a compound consistent with *m/z* MS^2^ of protoemetinol (Supplementary Fig. 7).

### Purification of epimer-pure DAII and DAI

An epimeric mixture of DAII and DAI was produced as described above but with some modifications. A 1.2-fold excess of dopamine hydrochloride was used, while the reaction was bubbled with argon and then incubated for 3 days. Preparative isolation was performed using an Xbridge BEH C18 OBD prep column 130 Å, 5 µm (Waters) on an Agilent 1260 HPLC system (with G7161A prep bin pump, G9328A column organizer and G7165A detector) and an Agilent 1290 G7159B fraction collector. The mobile phase was A:B where A was water with 0.1% formic acid and B was acetonitrile with 0.1% formic acid. The separation method was as follows: 5% B from 1 min to 25% B at 24 min, then the column was flushed at 100% B until 29 min and re-equilibrated to 5% B until 35 min. The flow rate was 8 ml min^−1^ and detection was performed at *λ* = 254 nm. Aliquots of fractions of each of the two main peaks were analyzed by UPLC–MS method 3, confirming that *m/z* and retention times corresponded to either of the two peaks typically observed in epimeric mixtures (Supplementary Fig. 5). Collected fractions of each peak were combined, dried using a freeze-dryer (Labconco) and subjected to nuclear magnetic resonance (NMR).

Deacetylisoipecoside (DAII, **4a**) was unstable during 2D NMR measurements in CH_3_OH-*d*_*3*_ and was, therefore, measured in acidic CH_3_OH-*d*_*3*_ (CH_3_OH-*d*_*3*_ + 0.1% formic acid), to prevent decomposition during long-term measurements. Deacetylipecoside (DAI **4b**) was lactamized even in acidic CH_3_OH-*d*_*3*_. Thus, the structure of **4b** was confirmed as demethylalangiside after complete lactamization. This observation was consistent with previously reported fact that the lactamization was much faster in the 1*R*-epimer than the 1*S-*epimer^104^.

### NMR analysis

NMR measurements were carried out on a 500-MHz Bruker Avance III HD spectrometer (Bruker Biospin) equipped with a TCI cryoprobe using standard pulse sequences as implemented in Bruker Topspin version 3.6.1. (Bruker Biospin) or a 400-MHz Bruker Avance III HD spectrometer (Bruker Biospin). Chemical shifts were referenced to the residual solvent signals of CDCl_3_ (δ_H_ 7.26/δ_C_ 77.16) or CH_3_OH-*d*_*3*_ (δ_H_ 3.31/δ_C_ 49.0) and coupling constants (*J*, Hz) are expressed in the following format: chemical shift value (multiplicity, coupling constant, integration). ^1^H-NMR spectral data are described using the following abbreviations: brs, broad singlet; s, singlet; d, doublet; t, triplet; q, quartet; dd, doublet of doublets; ddd, doublet of doublets of doublets; dt, doublet of triplets; td, triplet of doublets; appd, apparent doublet; m, multiplet.

On the basis of the structure determined from NMR analysis, a molecular model was created in GaussView version 6 (Semichem) and optimized using the semiempirical method PM6 in Gaussian version 16 (Gaussian). The optimized conformation from PM6 was used for rotating frame Overhauser enhancement spectroscopy (ROESY) analysis of protoemetine. For deacetylisoipecoside and demethylalangiside, the resulting structures were used for conformer variation with the GMMX processor of the Gaussian program package. Resulting structures were optimized through density functional theory (DFT) with Gaussian version 16 (B3LYP/6-31G(d), gas phase). The lowest-energy conformer from the DFT calculations was used for the ROESY analysis.

### Enzymatically produced standards and substrates

6-*O*-Me-DAIIA and 6-*O*-Me-DAIA were produced using recombinant CiDOMT1 and AsDOMT3, respectively. The reaction mix contained 50 mM HEPES pH 7.5, 1 mM total epimeric DAI(I)A (~500 µM per epimer) as substrate, 1 mM of potential metal cofactors MgCl_2_ and MnCl_2_, 1 mM SAM chloride dihydrochloride and 5 µM CiDOMT1 or 10 µM AsDOMT3. Reactions were incubated for 24 h at 30 °C and stopped by incubation at 98 °C for 10 min to inactivate enzymes. Precipitates were pelleted by centrifugation at 18,000*g* for 30 min. Supernatants were diluted 1:2 in H_2_O and used as substrates in leaf disk assays. Aliquots of the reactions were analyzed by UPLC–MS method 3 to observe expected products (Supplementary Fig. 5).

Confirmation of DAIIA and DAIA peak identity was achieved through deesterification of purified DAII and DAI, respectively, using recombinant CiDE. Reaction mixes contained 50 mM HEPES pH 7.5, 3 µM recombinant CiDE and 200 µM purified DAII or DAI. Reactions were incubated overnight at 30 °C and stopped by the addition of 50 µl of methanol supplemented with 0.1% FA. Eventual precipitates were pelleted by centrifugation at 18,000*g* for 30 min and supernatants were analyzed by UPLC–MS method 3 (Supplementary Fig. 5).

For in vitro competition assays of glucosidases (described below), 6-*O*-Me-DAIIA **6a** and 6-*O*-Me-DAIA **6b** were produced separately through coupling reactions. For 6-*O*-Me-DAIIA **6a**, the reaction mixture contained 50 mM HEPES pH 7.5, 1 mM purified DAII **4a** (described above), 1 mM MgCl_2_, 1 mM SAM, 40 µM CiDOMT1 and 50 µM CiDE. For 6-*O*-Me-DAIA **6b**, the reaction mixture contained 50 mM HEPES pH 7.5, 1 mM purified DAI (described above), 1 mM MgCl_2_, 1 mM SAM, 10 µM AsDOMT3 and 20 µM CiDE. Reactions were incubated for 24 h at 30 °C and stopped by incubation at 98 °C for 10 min. Precipitates were pelleted by centrifugation at 18,000*g* for 30 min. Aliquots of the supernatants were analyzed by UPLC–MS method 3, confirming that reactions were complete, and then used as substrates in assays (described below).

### In vitro competition activity assays of glucosidases

Compounds were mixed as 2× substrate master mixes (corresponding to calculated 200 µM per compound) and approximate equimolarity was checked by UPLC–MS analysis method 3. The substrate master mix for AsDGD2 and AsS6DGD assays contained all *A.* *salviifolium*-specific ipecac alkaloid glucosides: DAIIA **5a** and DAIA **5b** and 7-*O*-Me-DAIIA **12a** and 7-*O*-Me-DAIA **12b** (chemically synthesized as epimeric mixtures) and 6-*O*-Me-DAIIA **6a** and 6-*O*-Me-DAIA **6b** (enzymatically produced in epimer-pure form). The substrate mix to assay CiDGD and CiS6DGD contained *C.* *ipecacuanha-*specific ipecac alkaloid glucosides: ipecoside (commercially available), DAII **4a** and DAI **4b**, DAIIA **5a** and DAIA **5b**, 7-*O*-Me-DAII **18a** and 7-*O*-Me-DAI **18b** (chemically synthesized as epimeric mixtures) and 6-*O*-Me-DAIIA **6a** (enzymatically produced in epimer-pure form). Note that 7-*O*-Me-DAII **18a** and 7-*O*-Me-DAI **18b** were not detected in *C.* *ipecacuanha* extracts but were included in the assay because 7-*O*-Me-DAI **18b** can be produced by CiDOMT2 (Extended Data Fig. 4). Final assay mixes were prepared in triplicate at a final concentration of 50 mM HEPES pH 7.5, ~100 µM of each substrate and 0.5 µM of enzyme or boiled mixed enzyme as control. Boiled mixed enzymes were obtained by mixing the respective enzymes (AsDGD2 + AsS6DGD and CiDGD + CiS6DGD) at equimolar amounts, heating for 10 min at 98 °C and removing the precipitate through centrifugation at 18 000*g* for 20 min. Each replicate was pipetted separately on ice and immediately aliquoted into 25-µl samples on ice and then transferred to 30 °C and gentle shaking. At indicated time points, samples were snap-frozen. For the earliest time point (‘assay start’) samples were snap-frozen immediately after aliquoting on ice and before transfer to 30 °C. For UPLC–MS analysis, 75 µl of 70% methanol containing 0.1% formic acid and 2 mg L^−1^ caffeine as internal standard was added to each 25-µl sample. Samples were centrifuged for 30 min at 18,000*g* and room temperature to remove precipitates. Supernatants were analyzed using UPLC–MS method 3.

### Production of protoemetinol standard

Protoemetine was isolated in several batches from a total of 6 g of freshly harvested leaf buds and young leaves. The material was flash-frozen and ground with liquid nitrogen; then, 100% methanol was added at a ratio of 200 µl per 100 mg. Samples were sonicated in an ultrasonic bath for 10 min, incubated on a rotator for 30 min and centrifuged at 18,000*g* for 15 min. The supernatants were filtered through 0.22-µm syringe filters, diluted 1:10 in 100% methanol and subjected to semipreparative HPLC. Semipreparative isolation was performed using an XBridge BEH C18 column of 100 mm × 4.6 mm, 2.5 μm (Waters) on an Agilent 1260 Infinity HPLC system (with G1311B Quat pumps, G1315C diode array detectors, G1316A oven, G1329B autosampler and G1364F fraction collector). The mobile phase was A:B, where A was 0.1% formic acid in water and B was acetonitrile. The separation method was as follows: 8% B at 1 min to 13% B at 4 min, to 30% at 12 min. Then, the column was flushed at 100% B until 15 min and re-equilibrated to 8% B until 18 min. The flow rate was 2.4 ml min^−1^ and detection was performed at *λ* = 250 nm. Aliquots of collected fractions were analyzed using UPLC–MS method 3 and fractions containing the putative protoemetine peak, according to *m/z* and typical aldehyde peak shape, were combined and freeze-dried using a freeze-dryer (Labconco). Large freeze-dried fractions were solubilized in small volumes of 100% methanol, combined (around 350 µg) and dried under a nitrogen line. NMR was conducted on a Bruker Avance III HD 700-MHz spectrometer (Bruker Biospin), equipped with a TCI cryoprobe using standard pulse sequences implemented in Bruker Topspin version 3.6.1 (Bruker Biospin). Chemical shifts were referenced to CDCl_3_ residual solvent signals (*J*_H_ 7.26/*J*_C_ 77.16). NMR spectra of the sample were as follows: ^1^H-NMR (700 MHz,CDCl_3_): *δ* ppm: 0.93 (t, *J* = 7.5 Hz, 3H), 1.13 (m, 1H), 1.29 (m, 1H), 1.48 (m, 1H), 1.58 (m, 1H), 1.95 (m, 1H), 2.08 (dd, *J* = 11.3 Hz, 1H), 2.33 (m, 2H), 2.50 (ddd, *J* = 11.9, 11.4, 4.5 Hz, 1H), 2.63 (m, 1H), 2.72 (dd, *J* = 16.4, 3.3 Hz, 1H), 2.98 (m, 1H), 3.10 (m, 2H), 3.13 (m, 1H), 3.83 (s, 3H), 3.83 (s, 3H), 6.57 (s, 1H), 6.63 (s, 1H) and 9.88 (bs, 1H); ^13^C-NMR (175 MHz, CDCl_3_): *δ* ppm: 10.8, 23.6, 29.1, 35.7, 38.1, 41.3, 48.1, 52.3, 55.8, 55.8, 61.2, 62.6, 108.0, 111.4, 126.2, 129.4, 147.0, 147.3 and 202.4. This spectrum is in agreement with a previously published NMR spectrum of protoemetine^105^ and with that of chemically synthesized protoemetine (Supplementary Methods) in this study (Supplementary Fig. 26 and Supplementary Table 4). Isolated and synthesized protoemetine were, thus, identical.

### Reporting summary

Further information on research design is available in the Nature Portfolio Reporting Summary linked to this article.

## Online content

Any methods, additional references, Nature Portfolio reporting summaries, source data, extended data, supplementary information, acknowledgements, peer review information; details of author contributions and competing interests; and statements of data and code availability are available at 10.1038/s41589-025-01926-z.

## Supplementary information

Supplementary Information Supplementary Methods, Figs. 1–26, Tables 1–6 and References.

Reporting Summary

Supplementary Data 1 Accession numbers and amino acid sequences of genes used to construct phylogenetic trees.

## Source data

Source Data Fig. 2 Normalized peak areas.

Source Data Fig. 3 Normalized peak areas.

Source Data Fig. 4 Normalized peak areas.

Source Data Fig. 5 Normalized peak areas.

## Data Availability

There are no restrictions on the availability of the data. Illumina RNA-seq raw reads were uploaded in FASTQ format to the NCBI Sequence Read Archive under BioProject PRJNA1169657. All reported gene sequences were deposited to NCBI Genbank under the following accession numbers (Supplementary Table 6): AsDGD1 (PQ363556), AsDGD2 (PQ363557), AsDOMT1 (PQ363558), AsDOMT2 (PQ363559), AsDOMT3 (PQ363560), AsDOMT4 (PQ363561), AsDOMT5 (PQ363562), AsDOMT6 (PQ363563), AsDOMT7 (PQ363564), AsDPOMT1 (PQ363565), AsDPOMT2 (PQ363566), AsDR1 (PQ363567), AsDR2 (PQ363568), AsS6DGD (PQ363569), CiDE (PQ363570), CiDGD (PQ363571), CiDOMT1 (PQ363572), CiDOMT2 (PQ363573), CiDPOMT (PQ363574), CiDR1 (PQ363575), CiDR2 (PQ363576), CiIpS (PQ363577) and CiS6DGD (PQ363578). Sequences to construct phylogenetic trees were retrieved from public databases (https://blast.ncbi.nlm.nih.gov/Blast.cgi and https://db.cngb.org/onekp/) or assemblies made in this study; accession numbers and amino acid sequence are listed in Supplementary Dataset 1. Source data are provided with this paper.
