# Supplementary material for: Ipecac alkaloid biosynthesis in two evolutionarily distant plants
Source: Nat Chem Biol. 2025 Jun 3;21(11):1794–805. doi: 10.1038/s41589-025-01926-z (PMC12568652; doi:10.1038/s41589-025-01926-z)
Supplement: Supplementary file 1 — Supplementary Methods, Figs. 1–26, Tables 1–6 and References. [file 41589_2025_1926_MOESM1_ESM.pdf]

nature chemical biology

Supplementary information https://doi.org/10.1038/s41589-025-01926-z

# Ipecac alkaloid biosynthesis in two evolutionarily distant plants

In the format provided by the authors and unedited

**Table of contents**

**Supplementary Methods**……………………………………………………………………3
**Supplementary Figures**…………………………………………………………………..8
Supplementary Fig. 1. Photos of plants sampled for metabolite analysis and RNA-seq……………8
Supplementary Fig. 2. Extended metabolite content of *C. ipecacuanha* and *A. salviifolium* tissues ..9
Supplementary Fig. 3. Extracted ion chromatograms (EIC) and MS2 data of cephaeline, emetine and ipecoside of *A. salviifolium* roots and *C. ipecacuanha* rhizome compared to purchased authentic standards……………………………………………………………………………11
Supplementary Fig. 4. Extracted ion chromatograms and MS2 data of secologanin and secologanic acid of *A. salviifolium* roots and C. ipecacuanha rhizome compared to standards…………………13
Supplementary Fig. 5. Extracted ion chromatograms and MS2 data of compounds with same m/z DA(I)I epimers and 6-, or 7-O-methylated DA(I)A epimers of *A. salviifolium* roots and *C. ipecacuanha* rhizome……………………………………………………………………..15
Supplementary Fig. 6. Extracted ion chromatograms and MS2 data of deacetylisoipecosidic acid (DAIIA, 5a) and deacetylipecosidic acid (DAIA, 5b) of *A. salviifolium* roots and *C. ipecacuanha* rhizome compared to standards……………………………………………………..16
Supplementary Fig. 7. Extracted ion chromatograms and MS2 data of protoemetine and protoemetinol of *A. salviifolium* roots and *C. ipecacuanha* rhizome compared to standards………17
Supplementary Fig. 8. Enzymatic coupling of dopamine with secologanin or secologanic acid in endogenous plant protein extracts could not be detected…………………………………...19
Supplementary Fig. 9. Pathway reconstitution with *CiDGD* and *AsDGD1,2* does not lead to protoemetine formation…………………………………………………………………..21
Supplementary Fig. 10. Putative reduction mechanism of DRs and spontaneous decarboxylation..22
Supplementary Fig. 11. Assignment of 10-*O*-demethylprotoemetinol and 9-*O*-demethylprotoemetinol…………………………………………………………………...23
Supplementary Fig. 12. Additional CiDR and AsDR paralogs have partial redundancy with DR1s………………………………………………………………………………...25
Supplementary Fig. 13. Assignment of 9,10-*O*,*O*-didemethylprotoemetinol……………………26
Supplementary Fig. 14. Extracted ion chromatograms and MS2 data of 7-*O*-Me-DAI/I and putative 6-*O*-Me-DAI/I from *N. benthamiana* agroinfiltrations and standards…………………..………27
Supplementary Fig. 15. AsDOMT amino acid alignment…………………………………..28
Supplementary Fig. 16. *In vitro* substrate competition assays of recombinant *A. salviifolium* glucosidases……………………………………………………………………………29
Supplementary Fig. 17. Non-enzymatic background reactions of *C. ipecacuanha* specific ipecac alkaloid glucosides……………………………………………………………………30
Supplementary Fig. 18. In vitro substrate competition assays of recombinant *C. ipecacuanha* glucosidases……………………………………………………………………………32
Supplementary Fig. 19. Confocal laser scanning microscopy of glucosidases fused to eYFP……..34
Supplementary Fig. 20. Confocal laser scanning microscopy showing additional replicates of glucosidases fused to eYFP………………………………………………………………36
Supplementary Fig. 21. Confocal laser scanning microscopy of CiIpS, CiDE and CiDR1 fused to eYFP or mCerulean3……………………………………………………………………..37
Supplementary Fig. 22. Maximum likelihood tree of *A. salviifolium* versus *C. ipecacuanha* OMTs……………………………………………………………………………………..38
Supplementary Fig. 23. Maximum likelihood tree of *A. salviifolium* versus *C. ipecacuanha* glucosidases……………………………………………………………………………39
Supplementary Fig. 24. NMR data for deacetylisoipecoside ……………………..…………40
Supplementary Fig. 25. NMR data for demethylalangiside ……………………………………45
Supplementary Fig. 26. NMR data for protoemetine …………………………….……..….50
**Supplementary Tables**……………………………………………………………..……55
Supplementary Table 1. Prediction of nuclear localization signal peptide by DeepLOC2……..…..55

Supplementary Table 2. NMR chemical shifts for deacetylisoipecoside (500 MHz NMR in MeOH-*d*$_3$ + 0.1% formic acid)……………………………………………………………………..…..56
Supplementary Table 3. NMR chemical shifts for demethylalangiside (500 MHz NMR in MeOH-*d*$_3$)……………………………………………………………………………………...57
Supplementary Table 4. NMR chemical shifts for protoemetine (500 MHz NMR in $CDCl_3$)……..58
Supplementary Table 5. List of primers used in this study…………………………………………59
Supplementary Table 6. Accession numbers of genes described in this study……………………..62
**Supplementary references**…………………………………….….….….….….….…..…..63

# Supplementary methods

## Chemical synthesis of (–)-protoemetine

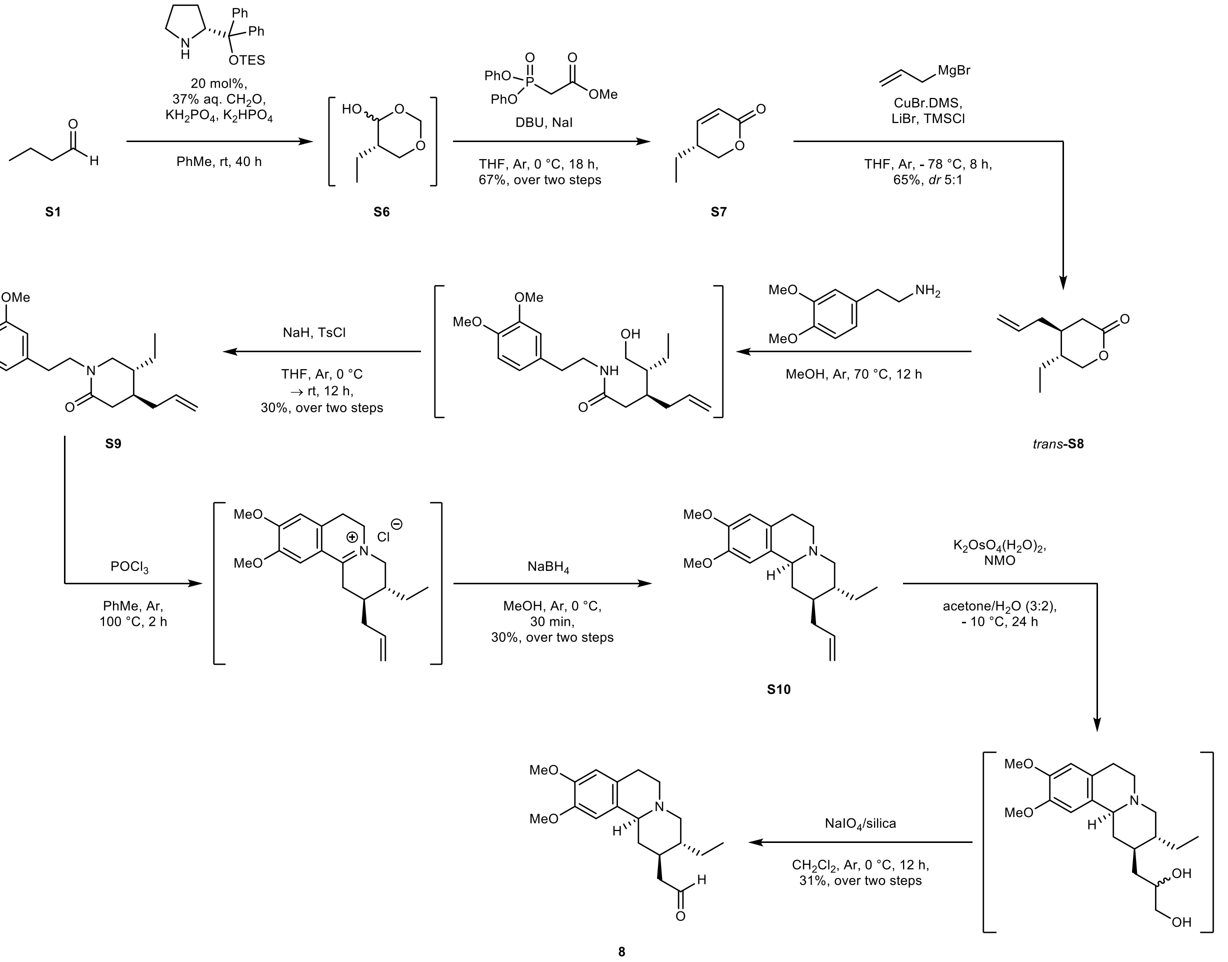


**Scheme 1** Total synthesis of (−)-protoemetine (**8**) [1]

### General experimental note

All reagents were obtained from commercial sources and used without any further purification. Thin layer chromatography (TLC) was carried out on aluminum-backed silica gel 60 $F_{254}$ plates (Merck) that were visualized with Hanessian's stain or using $UV_{254\ nm}$ light detection. Wet flash column chromatography was performed using Merck Kieselgel 60 (particle size 0.040–0.063 mm, density 0.8 g/cm$^3$).

### (*R*)-2-{Diphenyl[(triethylsilyl)oxy]methyl }pyrrolidine (S2)

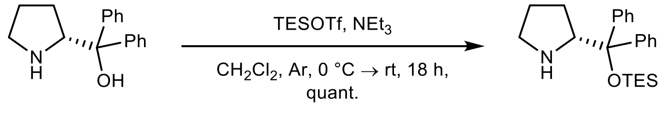


**S1** **S2**

Briefly, following a modified procedure reported previously [2], to a dry 50 mL round bottom flask under an Ar atmosphere, containing a mixture of (*R*)-diphenyl(pyrrolidin-2-yl)methanol (**S1**,1 g, 3.95 mmol) and $NEt_3$ (0.72 mL, 5.14 mmol) in anhydrous $CH_2Cl_2$ (26 mL) at 0 °C, was slowly added

TESOTf (1.16 mL, 5.14 mmol). The reaction mass was allowed to warm to room temperature and stir for 18 h. To the resulting colorless solution was added $H_2O$ (30 mL). The mixture was extracted with $CH_2Cl_2$ (10 mL x 3) and the combined organic layers were dried over anhydrous $Na_2SO_4$. Following concentration under reduced pressure, the colorless residue obtained was purified by gradient flash column chromatography (0–10% MeOH in $CH_2Cl_2$) to afford title compound **S2** as a colorless oil (1.450 g, quant.): $^1$H NMR ($CDCl_3$, 400 MHz) $\delta$ 7.48–7.45 (m, 2H), 7.37–7.34 (m, 2H), 7.30–7.22 (m, 6H, overlapping with residual $CHCl_3$), 4.05 (t, $J$ = 7.4 Hz, 1H), 2.87–2.81 (m, 1H), 2.71–2.65 (m, 1H), 1.95 (brs, 1H), 1.64–1.52 (m, 3H), 1.29–1.22 (m, 1H), 0.85 (t, $J$ = 7.9 Hz, 9H), 0.35 (q, $J$ = 7.9 Hz, 6H). Spectral and physical data agreed with those reported previously [3].

**Methyl 2-(diphenoxyphosphoryl)acetate (S4)**

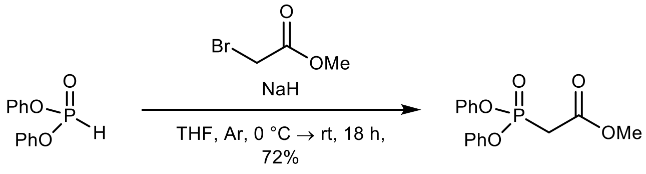


**S3** **S4**

Following a previously reported procedure [4], to a dry 250 mL three-neck round bottom flask equipped with a dropping funnel under an Ar atmosphere was added anhydrous THF (31 mL) and NaH (60% dispersion in mineral oil, 3.2 g, 80 mmol). Upon cooling to 0 °C, a solution of diphenyl phosphite (**S3**, 15.36 mL, 80 mmol) in anhydrous THF (10.3 mL) was slowly added to the mixture. After stirring at 0 °C for 1 h, a solution of methyl bromoacetate (7.55 mL, 80 mmol) in anhydrous THF (20 mL) was then added dropwise over 2 h to the reaction mass that was then subsequently allowed to warm to room temperature and stir for a further 18 h. The resulting colorless suspension was cooled to 0 °C and quenched with sat. aq. $NH_4Cl$ (40 mL) and $H_2O$ (50 mL). The mixture was extracted with $Et_2O$ (100 mL x 3) and the combined organic layers dried over $Na_2SO_4$. Concentration under reduced pressure afforded an opaque residue that was further purified by gradient flash column chromatography (0–50% EtOAc in hexane) to yield title compound **S4** as a colorless oil (17.736 g, 72%): $^1$H NMR ($CDCl_3$, 400 MHz) $\delta$ 7.36–7.30 (m, 4H), 7.24–7.16 (m, 6H), 3.77 (s, 3H), 3.28 (d, $J_{PH}$ = 21.6 Hz, 2H). Spectral and physical data agreed with those reported previously [5].

**(*R*)-5-Ethyl-5,6-dihydro-2*H*-pyran-2-one (S7)**

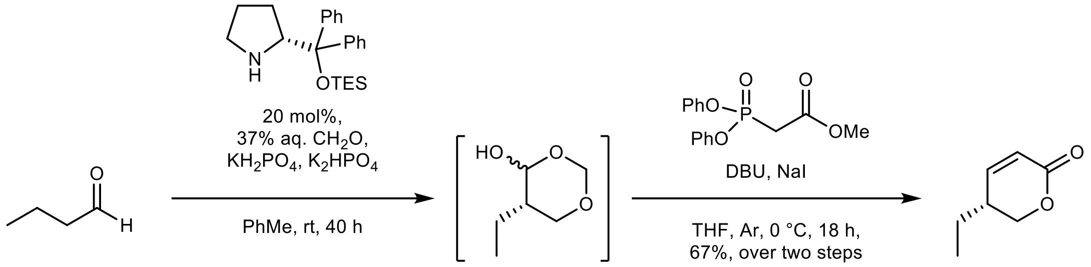


**S5** **S6** **S7**

Following a previously reported procedure [1], to a dry 100 mL round bottom flask under an Ar atmosphere containing the silyl ether **S2** shown above (1.452 g, 3.95 mmol), $KH_2PO_4$ (618 mg, 4.54 mmol), $K_2HPO_4$ (1.099 g, 6.31 mmol), in toluene (9.9 mL), was added 37% aq. $CH_2O$ (5.89 mL, 72.57 mmol). After stirring at room temperature for 10 min, the mixture was cooled to 0 °C and charged with butyraldehyde (**S5**, 1.78 mL, 19.72 mmol). The reaction mixture was allowed to warm

to room temperature and then rapidly stir for a further 40 h. The resulting biphasic mixture was extracted with toluene (5 mL x 6) and the combined organic layers were washed with brine (10 mL) and dried over anhydrous $Na_2SO_4$. The organic phase was carefully dried *in vacuo* (40 mbar at 30 °C) to afford the hemiacetal **S6** which was then immediately carried forward without any further purification to the next reaction step. To a dry three-neck round bottom flask fitted with a dropping funnel under an Ar atmosphere, containing anhydrous THF (143 mL), was added diphenyl phosphonate **S4** (17.514 g, 57.19 mmol) and NaI (9.458 g, 63.10 mmol). The resulting solution was cooled to 0 °C and then charged with DBU (7.03 mL, 47.13 mmol). After stirring at 0 °C for 30 min, to the reaction mass was then slowly added a solution of crude hemiacetal **S6** in anhydrous THF (33 mL). After stirring at 0 °C for 24 h, the colorless suspension was charged with 1 M aq. HCl (50 mL) and then further diluted with $H_2O$ (100 mL). The mixture was then extracted with EtOAc (100 mL x 3) and the combined organic layers were washed with brine (100 mL) and dried over anhydrous $Na_2SO_4$. Concentration under reduced pressure gave an amber residue that was further purified by gradient flash column chromatography (0–6.25% EtOAc in petroleum ether [b.p. 40–60 °C]) to afford pentenolide **S7** as a light yellow oil (1.673 g, 67% [over two steps]): $^1H$ NMR ($CDCl_3$, 400 MHz) $\delta$ 6.86 (dd, $J$ = 9.8, 3.8 Hz, 1H), 5.99 (dd, $J$ = 9.8, 1.8 Hz, 1H), 4.42 (dd, $J$ = 11.1, 5.0 Hz, 1H), 4.15 (dd, $J$ = 11.1, 7.4 Hz, 1H), 2.47–2.39 (m, 1H), 1.59–1.47 (m, 2H), 1.02 (t, $J$ = 7.5 Hz, 3H). Spectral and physical data agreed with those reported previously [1].

### (4*S*,5*R*)-4-Allyl-5-ethyltetrahydro-2*H*-pyran-2-one (*trans*-S8)

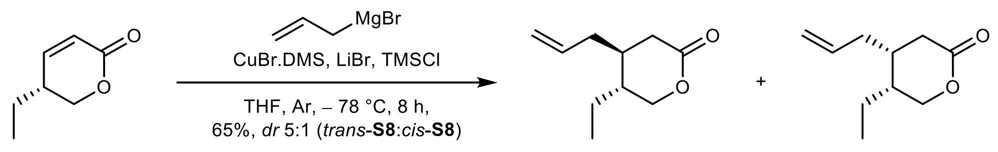


**S7** *trans*-**S8** *cis*-**S8**

Following a previously reported procedure [1], to a dry 250 mL three-neck round bottom flask fitted with a dropping funnel under an Ar atmosphere, was added CuBr.DMS (5.452 g, 26.52 mmol), LiBr (2.303 g, 26.52 mmol), and anhydrous THF (88 mL). Upon cooling to – 78 °C, the resulting suspension was slowly charged with a 0.7 M solution of allylmagnesium bromide in THF (39.79 mL, 27.85 mmol) over 30 min and then stirred at – 78 °C for a further 10 min, followed by the addition of trimethylsilyl chloride (6.33 mL, 49.86 mL). After stirring for 10 min at – 78 °C, a solution of pentenolide **S7** (1.673 g, 13.26 mmol) in anhydrous THF (10 mL) was added dropwise to the reaction mixture. Upon stirring for 8 h at – 78 °C, the dark brown reaction mass was partitioned between a mixture of $CH_2Cl_2$ (100 mL) and $H_2O$ (100 mL) and then filtered through a short pad of Celite®. The solids were washed with $CH_2Cl_2$ (10 mL x 3). The combined filtrate was then separated and the resulting aqueous layer extracted with $CH_2Cl_2$ (100 mL x 3). The combined organic layers were washed with brine (200 mL) and dried over anhydrous $Na_2SO_4$. Concentration under reduced pressure afforded an amber oil that was subsequently purified by gradient flash column chromatography (0–6.25% EtOAc in petroleum ether [b.p. 40–60 °C]) to yield a mixture of *trans*-**S8** and *cis*-**S8** (*dr* 5:1, respectively) as a colorless oil (1.457 g, 65% [over two steps]): $^1H$ NMR ($CDCl_3$, 400 MHz) *trans*-**S8*** $\delta$ 5.77–5.65 (m, 1H), 5.11–5.06 (m, 2H), 4.29 (dd, $J$ = 11.5, 4.4 Hz, 1H), 4.00 (dd, $J$ = 11.4, 7.5 Hz, 1H), 2.58 (dd, $J$ = 16.3, 6.8 Hz, 1H), 2.28 (overlapping dd, $J$ = 16.3, 7.7 Hz, 1H), 2.27–2.20 (overlapping m, 1H), 1.84–1.75 (m, 1H), 1.60–1.48 (m, 2H), 1.42–1.29 (m, 1H), 0.94 (t, $J$ = 7.4 Hz, 3H). Spectral and physical data agreed with those reported previously [1].

*Isolation of *trans* was achieved by iterative gradient flash column chromatography (0–6.25% EtOAc in petroleum ether [b.p. 40–60 °C]).

**(4*S*,5*R*)-4-Allyl-1-(3,4-dimethoxyphenethyl)-5-ethylpiperidin-2-one (S9)**

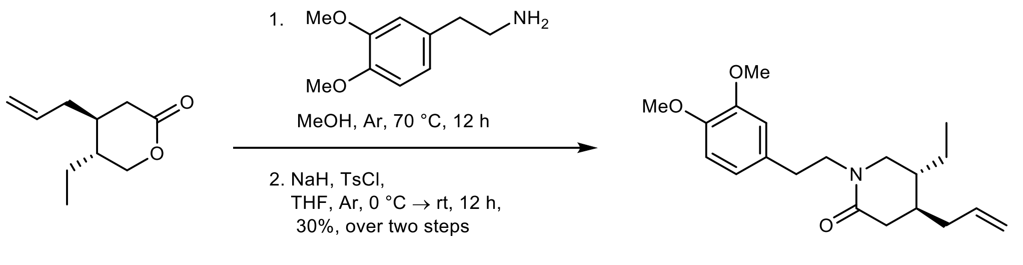


*trans*-**S8** **S9**

To a dry 15 mL pressure tube under an Ar atmosphere was added *trans*-**S8** (500 mg, 2.97 mmol) anhydrous MeOH (1.43 mL), and homoveratrylamine (0.75 mL, 4.46 mmol). The mixture was heated to 70 °C and allowed to stir for 12 h after which thin-layer chromatography (33% EtOAc in in petroleum ether [b.p. 40–60 °C]) indicated the complete consumption of starting material *trans*-**S8**. Concentration of the reaction mass under reduced pressure gave a residue that was then transferred to a dry 100 mL round bottom flask under an Ar atmosphere and dissolved in anhydrous THF (20 mL). Upon cooling to 0 °C, the solution was charged with 60% (w/w) NaH in mineral oil (549 mg, 13.72 mmol). After stirring for 30 min at 0 °C, a solution of TsCl (1.416 g, 7.43 mmol) in anhydrous THF (7.4 mL) was then slowly added to the resulting suspension and the reaction mass allowed to warm to room temperature. After stirring for 12 h, $H_2O$ (10 mL) was added. The mixture was extracted with EtOAc (10 mL x 5) and the combined organic layers were washed with brine (20 mL) and dried over anhydrous $Na_2SO_4$. Concentration *in vacuo* gave a residue that was further purified by gradient flash column chromatography (50% EtOAc in petroleum ether [b.p. 40–60 °C]) to yield lactam **S9** as a yellow oil (293 mg, 30% [over two steps from *trans*-**S8**]): $^1$H NMR ($CDCl_3$, 400 MHz) $\delta$ 6.81–6.76 (m, 3H), 5.75–5.65 (m, 1H), 5.07–4.99 (m, 2H), 3.87 (s, 3H), 3.85 (s, 3H), 3.64–3.65 (m, 1H), 3.52–3.45 (m, 1H), 3.13 (dd, $J$ = 12.3, 5.0 Hz, 1H), 2.88–2.82 (m, 3H), 2.47 (dd, $J$ = 17.5, 5.6 Hz, 1H), 2.22–2.16 (m, 1H), 2.10 (dd, $J$ = 17.5, 8.8 Hz, 1H), 1.99–1.92 (m, 1H), 1.60–1.50 (m, 1H), 1.48–1.40 (m, 1H), 1.25–1.17 (m, 1H), 0.83 (t, $J$ = 7.4 Hz, 3H). Spectral and physical data agreed with those reported previously [1].

**(2*S*,3*R*,11b*S*)-2-Allyl-3-ethyl-9,10-dimethoxy-1,3,4,6,7,11b-hexahydro-2*H*-pyrido[2,1-*a*]isoquinoline (S10)**

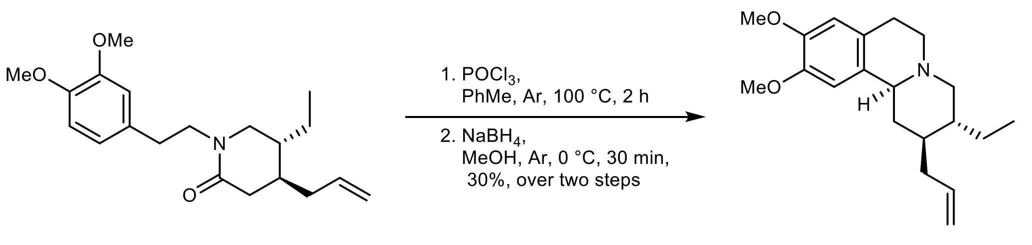


**S9** **S10**

Briefly following a previously reported procedure [1], to a dry 50 mL round bottom flask under an Ar atmosphere, containing lactam **S9** (293 mg, 0.88 mmol) in anhydrous PhMe (10 mL), was added $POCl_3$ (0.33 mL, 3.52 mmol). The mixture was heated to 100 °C for 2 h and then concentrated *in vacuo* to afford a brown residue that was subsequently dissolved in anhydrous MeOH (10 mL). Upon cooling to 0 °C, the solution was charged with $NaBH_4$ (67 mg, 1.76 mmol) and then allowed to stir

for a further 30 min. The resulting bright yellow solution was concentrated under reduced pressure to afford a residue that was partitioned between EtOAc (10 mL) and $H_2O$ (30 mL). The aqueous layer was extracted with EtOAc (10 mL x 2) and the resulting organic layers were combined, dried over anhydrous $MgSO_4$, and concentrated *in vacuo* to give a residue that was purified by gradient flash column chromatography (10–50% EtOAc in petroleum ether [b.p. 40–60 °C]) to afford title compound **S10** as a yellow oil (88 mg, 30% [over two steps from lactam **S9**]): $^1$H NMR ($CDCl_3$, 400 MHz) $\delta$ 6.68 (s, 1H), 6.56 (s, 1H), 5.89–5.78 (m, 1H), 5.06–5.02 (m, 2H), 3.84 (s, 3H), 3.83 (s, 3H), 3.14–3.03 (m, 3H), 2.97 (ddd, $J$ = 11.2, 5.9, 1.6 Hz, 1H), 2.61 (d, $J$ = 16.0 Hz, 1H), 2.46 (td, $J$ = 11.5, 4.1 Hz, 1H), 2.42–2.37 (m, 1H), 2.26 (dt, $J$ = 12.9, 3.1 Hz, 1H), 2.04–1.95 (m, 2H), 1.72–1.64 (m, 1H), 1.54–1.30 (m, 3H), 1.27–1.20 (m, 1H), 1.18–1.09 (m, 1H), 0.91 (t, $J$ = 7.5 Hz, 3H). Spectral and physical data agreed with those reported previously [1].

### 2-[(2*R*,3*R*,11b*S*)-3-Ethyl-9,10-dimethoxy-1,3,4,6,7,11b-hexahydro-2*H*-pyrido[2,1-*a*]isoquinolin-2-yl]acetaldehyde (8)

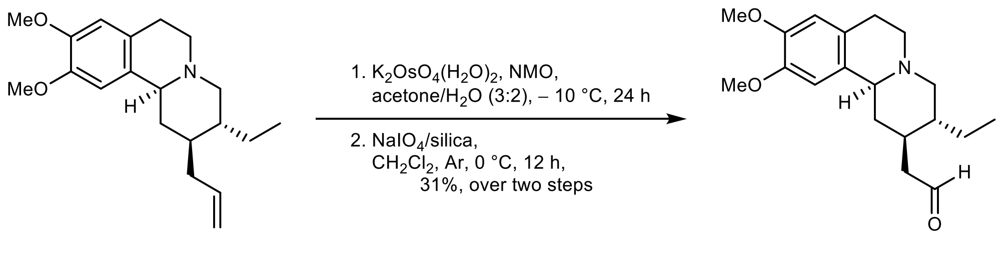


**S10** **8**

Following a previously reported procedure [1], to a 50 mL round bottom flask, containing alkene **S10** (81 mg, 0.26 mmol) and *N*-methylmorpoline *N*-oxide (137 mg, 1.17 mmol) in acetone/$H_2O$ (3:2, 13 mL) at – 10 °C, was added $K_2OsO_4(H_2O)_2$ (5.75 mg, 0.016 mmol). After stirring for 12 h, a second portion of $K_2OsO_4(H_2O)_2$ was added to the reaction mass and the mixture was further stirred at – 10 °C. After 12 h, the resulting solution was diluted with *n*-BuOH (10 mL) and charged with 0.4 M aq. $Na_2SO_3$ (10 mL). Repeated extraction of the aforementioned mixture with *n*-BuOH (10 mL x 3) gave a combined organic phase that was subsequently washed with sat. aq. $NaHCO_3$ (10 mL) and brine (10 mL) and then dried over anhydrous $Na_2SO_4$. Concentration under reduced pressure afforded a residue that was dissolved in anhydrous $CH_2Cl_2$ (6.5 mL) and cooled to 0 °C under Ar. The solution was charged with freshly prepared silica-supported $NaIO_4$ [6] (819 mg, 0.26 mmol) and stirred at 0 °C for 12 h. Filtration of the reaction mixture through a short pad of Celite® gave a filtrate that was concentrated *in vacuo* to afford a residue that was further purified by gradient flash column chromatography (0–6.25% MeOH in EtOAc) to furnish (−)-protoemetine (**8**) as a light yellow oil (25 mg, 31% [over two steps from alkene **S10**]): NMR data is shown in Supplementary Fig. 27. HRMS (ESI/Q-TOF) *m/z*: $[M+H]^+$ Calcd. for $C_{19}H_{28}NO_3$ 318.2064; Found 318.2059 (− 1.6 ppm). Spectral and physical data agreed with those reported previously [7].

## Supplementary Figures

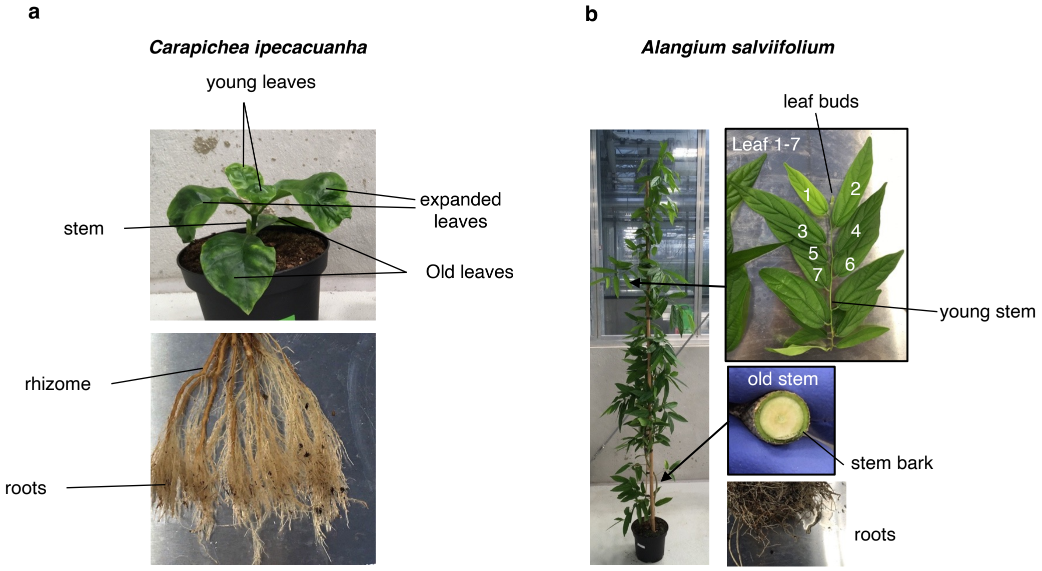


**Supplementary Fig. 1. Photos of plants sampled for metabolite analysis and RNA-seq. a**, *C. ipecacuanha* plants were regenerated from *in vitro* culture and grown on soil for four months. **b**, *A. salviifolium* plants were grown from cuttings harvested after 14 months. Plants were grown under controlled conditions in a greenhouse (12/12 hours light/dark 28-30/ 24-26°C°). Indicated tissues were used for RNA-seq and metabolomics analysis. Metabolomics was performed using three individual plants of the same age, RNA-seq was performed using material from one individual plant (shown on the picture).

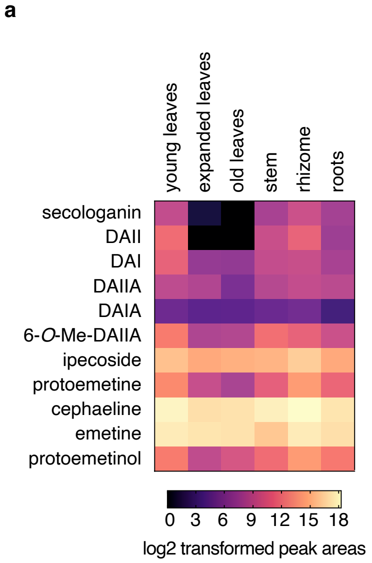


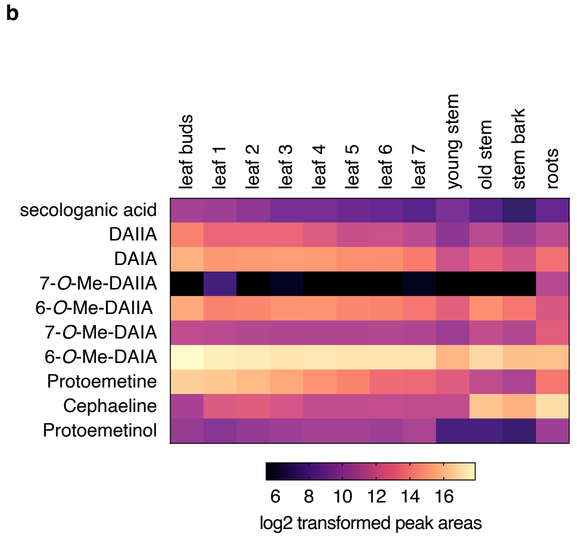


**Supplementary Fig. 2. Extended metabolite content of *C. ipecacuanha* and *A. salviifolium* tissues.** Heatmap depicts log2 transformed mean of LC-MS peak areas from three biological replicates of *C. ipecacuanha* (**a**) and *A. salviifolium* (**b**). Data is from the same experiment shown in main text Fig. 2 e-f.

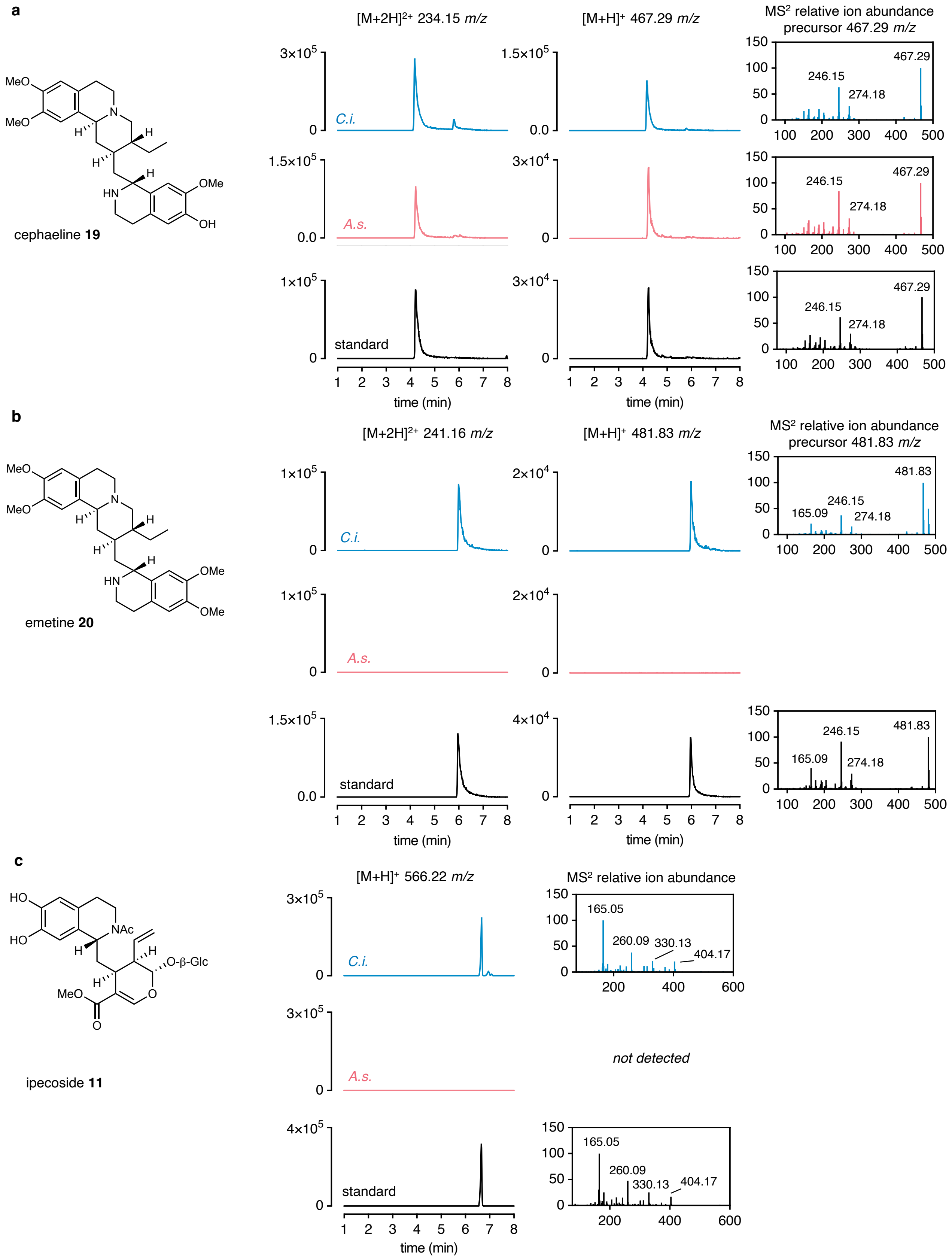
a
cephaeline 19
MeO
MeO
OMe
OH
HN
N
H
[M+2H]2+ 234.15 m/z
[M+H]+ 467.29 m/z
MS2 relative ion abundance
precursor 467.29 m/z
C.i.
A.s.
standard
3×10^5
1.5×10^5
1×10^5
3×10^4
467.29
246.15
274.18
time (min)
b
emetine 20
[M+2H]2+ 241.16 m/z
[M+H]+ 481.83 m/z
MS2 relative ion abundance
precursor 481.83 m/z
2×10^4
4×10^4
481.83
246.15
165.09
274.18
c
ipecoside 11
HO
HO
NAc
O-β-Glc
MeO
O
[M+H]+ 566.22 m/z
MS2 relative ion abundance
4×10^5
165.05
260.09
330.13
404.17
not detected

**Supplementary Fig. 3. Extracted ion chromatograms (EIC) and MS$^2$ data of cephaeline, emetine and ipecoside of *A. salviifolium* roots and *C. ipecacuanha* rhizome compared to purchased authentic standards. a,** cephaeline, **b,** emetine, **c**, ipecoside. All chromatograms are extracted from the same LC-MS experiment, which is the same as shown in main text Fig. 2a. EIC for *C. ipecacuanha* extract is shown in blue and labelled with *C.i.* as abbreviation; for *A. salviifolium* in magenta, abbreviated with *A.s.*; and for the respective standards in black. For cephaeline and emetine $[M+2H]^{2+}$ and $[M+H]^{+}$ ions are detected. MS$^2$ data is shown as relative abundance of ions with *m/z* values of the most abundant fragment ions indicated. Ipecoside and emetine were not detected in *A. salviifolium* extracts.

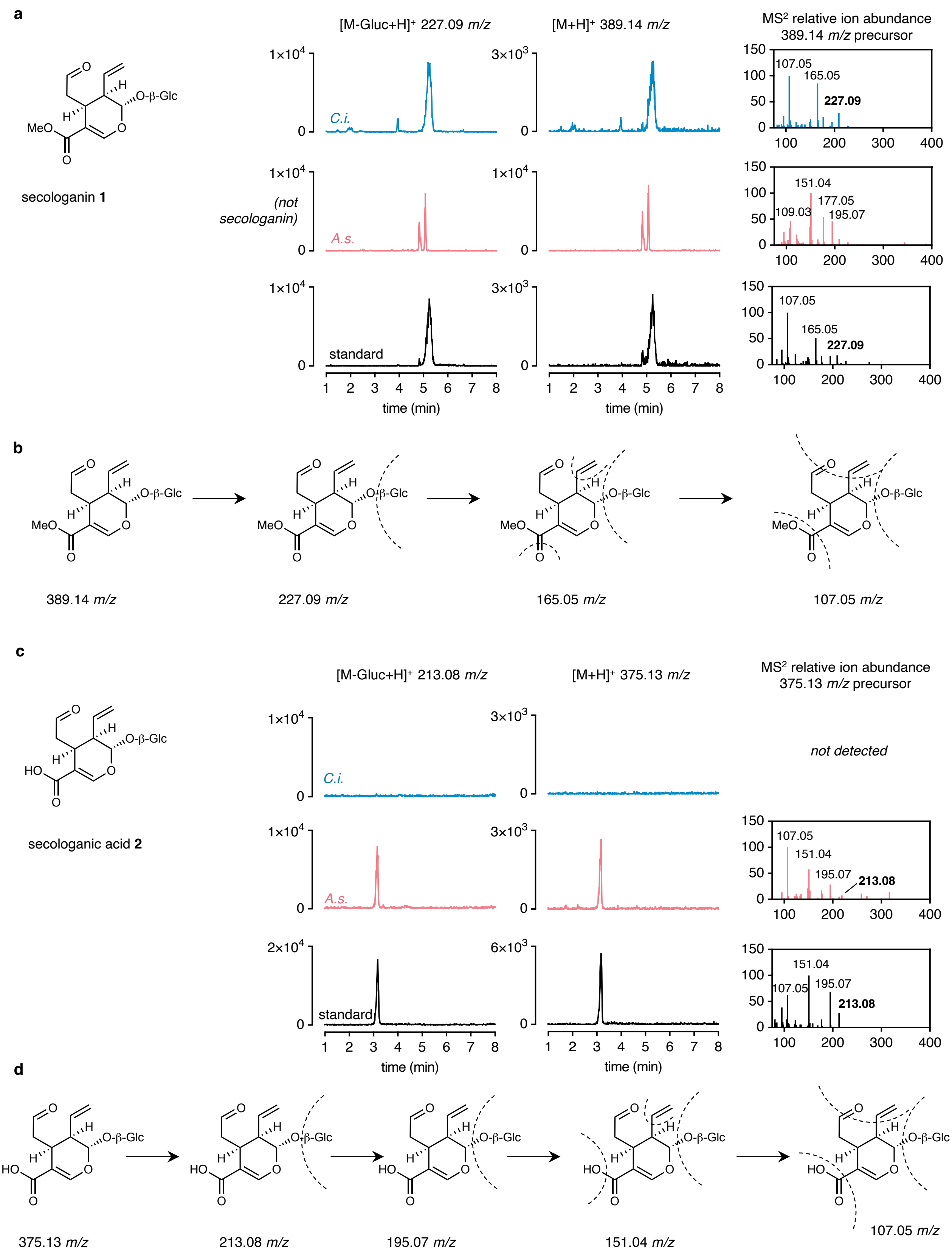
a
[M-Gluc+H]+ 227.09 m/z
[M+H]+ 389.14 m/z
MS2 relative ion abundance 389.14 m/z precursor
secologanin 1
C.i.
(not secologanin)
A.s.
standard
time (min)
107.05
165.05
227.09
151.04
177.05
109.03
195.07
b
389.14 m/z
227.09 m/z
165.05 m/z
107.05 m/z
c
[M-Gluc+H]+ 213.08 m/z
[M+H]+ 375.13 m/z
MS2 relative ion abundance 375.13 m/z precursor
not detected
secologanic acid 2
213.08
d
375.13 m/z
213.08 m/z
195.07 m/z
151.04 m/z
107.05 m/z

**Supplementary Fig. 4. Extracted ion chromatograms and MS$^2$ data of secologanin and secologanic acid of *A. salviifolium* roots and *C. ipecacuanha* rhizome compared to standards. a**, data for secologanin. Authentic standard is commercially available. An in-source fragment ion (chromatogram on the left), likely secologanin aglycone, is detetected in addition to the $[M+H]^+$ ion (chromatogram on the right). An *m/z* identical to the MS$^1$ of secologanin is detected in *A. salviifolium* extracts but peak shape, retention time and MS$^2$ fragmentation pattern indicate that this compound is not secologanin. These compounds are likely vogeloside and epivogeloside that have previously been reported to form from secologanic acid [8]. We have also observed these peaks when secologanic acid standard is diluted in methanol prior to LC-MS analysis. **b,** proposed fragmentation of secologanin. **c**, data for secologanic acid. An in source fragment ion (chromatogram on the left), presumably secologanic acid aglycone, is detected in addition to the $[M+H]^+$ ion (chromatogram on the right). **d**, Proposed MS$^2$ fragmentation pattern for secologanic acid. Secologanic acid standard was produced from purchased secologanin through alkaline hydrolysis (see methods). LC-MS and MS$^2$ data of the confirmed the correct identity of secologanic acid as the resulting product. *C. ipecacuanha* data is shown in blue and labelled with *C.i.* as abbreviation; *A. salviifolium* data in magenta, abbreviated with *A.s.*; and data for the respective standards in black. MS$^2$ data is shown as relative abundance of ions with *m/z* values of the most abundant fragment ions indicated.

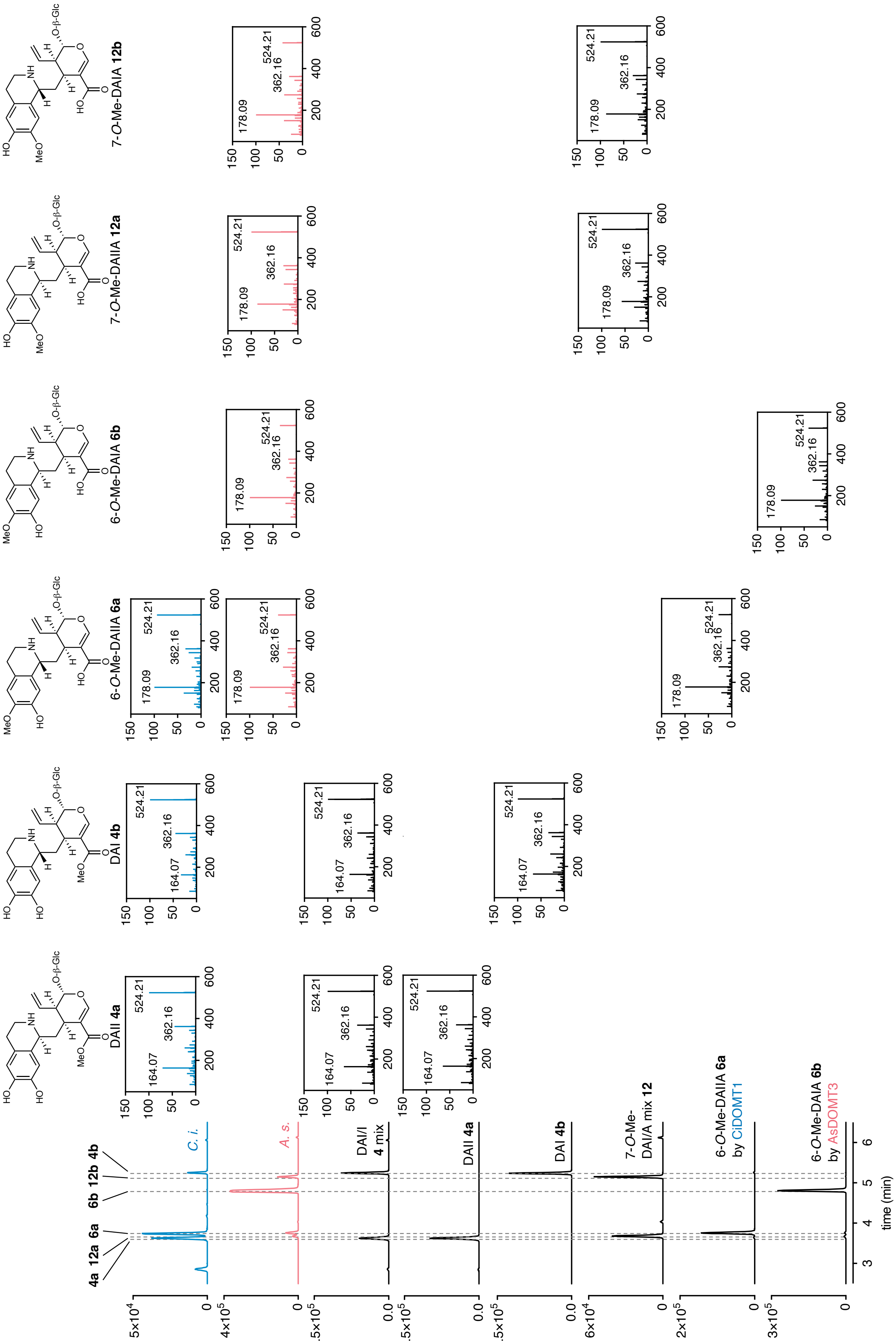

DAII 4a
DAI 4b
6-O-Me-DAIIA 6a
6-O-Me-DAIA 6b
7-O-Me-DAIIA 12a
7-O-Me-DAIA 12b
O-β-Glc
C. i.
A. s.
DAI/I 4 mix
7-O-Me-DAI/A mix 12
6-O-Me-DAIIA 6a by CiDOMT1
6-O-Me-DAIA 6b by AsDOMT3
4a 12a 6a
6b 12b 4b
time (min)
164.07
178.09
362.16
524.21

**Supplementary Fig. 5. Extracted ion chromatograms and MS$^2$ data of compounds with same m/z DA(I)I epimers and 6-, or 7-*O*-methylated DA(I)A epimers of *A. salviifolium* roots and *C. ipecacuanha* rhizome.** Left: EICs for 524.22 *m/z*. From top to bottom: *C. ipecacuanha* data (*C.i.*, blue), *A. salviifolium* (*A.s.*, magenta), DAII/DAI **4a/4b** epimer mix, purified DAII **4a** (stereochemistry confirmed by NMR), purified DAI **4b** (stereochemistry confirmed by NMR), 7-*O*-Me-DAI/IA **12a/b** epimer mixture, tentative 6-*O*-Me-DAIIA **6a** produced by recombinant CiDOMT1, and tentative 6-*O*-Me-DAIA **6b** produced by recombinant AsDOMT3 (see methods). The mixtures of epimers were produced by Pictet-Spengler reaction of dopamine with secologanin in case of DAI/I or 4-*O*-Me-dopamine with secologanic acid for DAIA/DAIIA (see methods). The reactions yield *S* and *R* epimers at a ratio of approximately 40:60. Minor peaks with the same *m/z* values are consistently observed when performing these reactions and could correspond to "neo" isomers which were previously reported to be minor by products of the reaction of the similar DAII and DAI [9]. On top the molecular structure of each detected molecule is shown. MS$^2$ fragmentations are shown wherever each of these molecules were detected. The main distinction criterion between *O*-methylated acid epimers and DAI/I is a fragment derived from the dopamine moiety which is 178.09 *m/z* when this moiety is methylated and 164.07 *m/z* when not methylated. These signals are labelled in each MS$^2$ fragmentation. The difference in *m/z* is indicative of a methyl group (+14). MS$^2$ fragmentation for 7-*O*-Me-DAI(I)A epimers and tentative 6-*O*-Me-DAI(I)A is identical but the retention time is shifted. Therefore, it can be assumed that the indicated peaks are indeed the 6-*O*-methylated compounds. MS$^2$ data is shown as relative abundance of ions with *m/z* values of the most abundant fragment ions indicated.

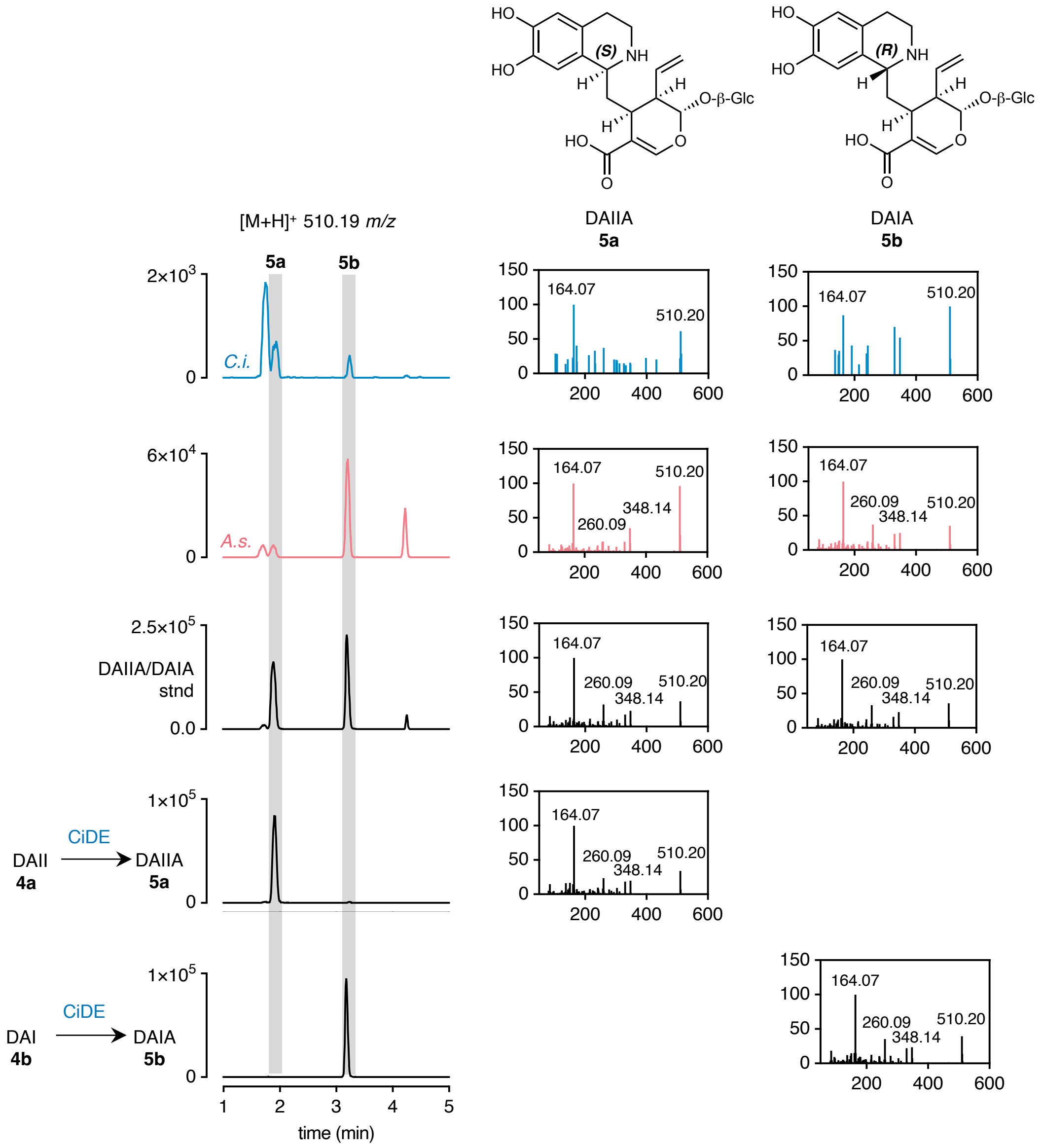


**Supplementary Fig. 6. Extracted ion chromatograms and MS$^2$ data of deacetylisoipecosidic acid (DAIIA, 5a) and deacetylipecosidic acid (DAIA, 5b) of *A. salviifolium* roots and *C. ipecacuanha* rhizome compared to standards.** The mixture of epimers was produced by Pictet-Spengler reaction of dopamine with secologanic acid (see methods). The reactions yield *S* and *R* epimers at a ratio of approximately 40:60. Minor peaks are with the same *m/z* values are consistently observed when performing these reactions and could correspond to "neo" isomers which were previously reported [9]. To confirm epimer specific peak identity epimer pure DAII (**4a**) or DAI (**4b**) (for which stereochemistry was confirmed by NMR, see below), respectively, were deesterified using recombinant CiDE. This confirmed the first peak as *S* epimer and the second as the *R* epimer. DAIIA (**5a**) and DAIA (**5b**) are also observed in *C. ipecacuanha*, albeit in low amounts. *C. ipecacuanha* data is shown in blue and labelled with *C.i.* as abbreviation; *A. salviifolium* data in magenta, abbreviated with *A.s.*; and data for the respective standards in black as indicated. MS$^2$ data is shown as relative abundance of ions with *m/z* values of the most abundant fragment ions indicated.

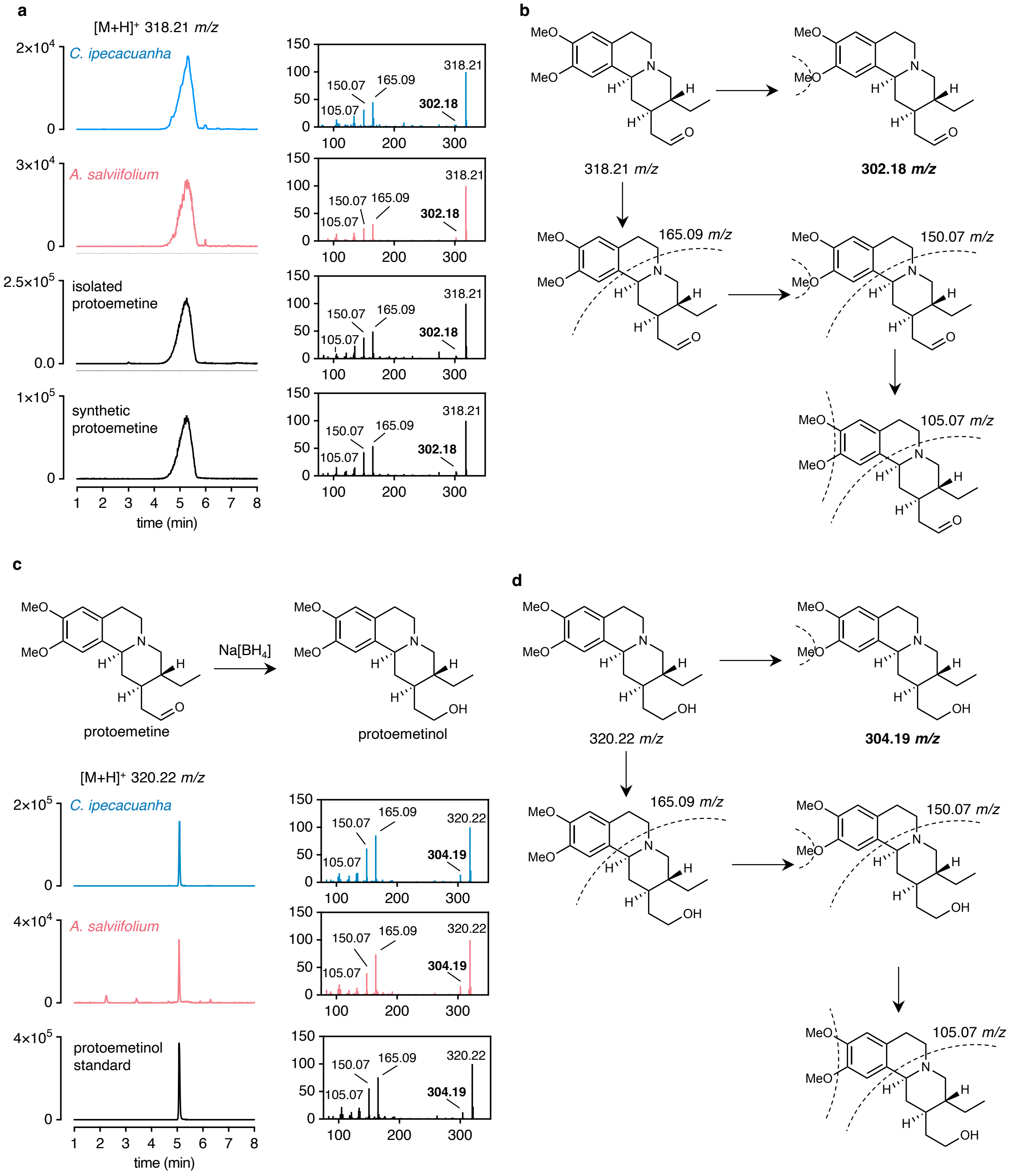


**Supplementary Fig. 7. Extracted ion chromatograms and MS$^2$ data of protoemetine and protoemetinol of *A. salviifolium* roots and *C. ipecacuanha* rhizome compared to standards. a**, data for protoemetine. Two chemically identical protoemetine standards were used throughout this study. A limited amount of protoemetine was isolated from *A. salviifolium* leaf buds and NMR data was in agreement with published data. An additional standard was obtained through chemical synthesis (see methods, full NMR spectra see Supplementary Fig. 24). **b**, proposed fragmentation of protoemetine. **c**, data for protoemetinol. Protoemetinol was produced through $NaBH_4$ reduction of protoemetine. MS data was consistent with values expected for protoemetinol. **d**, proposed MS$^2$ fragmentation of protoemetinol. *C. ipecacuanha* data is shown in blue and labelled with *C.i.* as abbreviation; *A. salviifolium* data in magenta, abbreviated with *A.s.*; and data for the respective standards in black as indicated. MS$^2$ data is shown as relative abundance of ions with *m/z* values of

the most abundant fragment ions indicated and additionally a fragment that shows expected difference between protoemetine and protoemetinol.

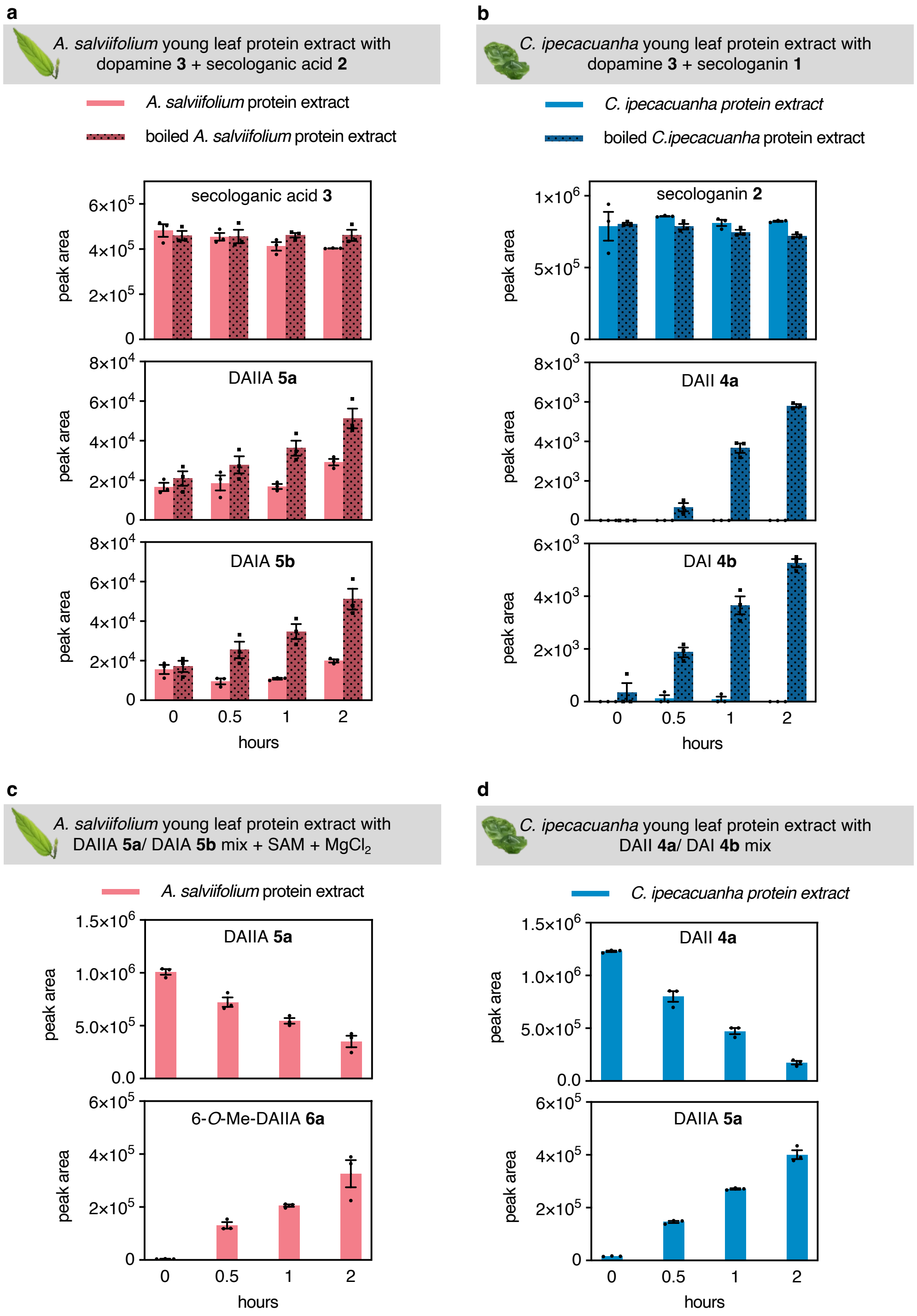


**Supplementary Fig. 8. Enzymatic coupling of dopamine with secologanin or secologanic acid in endogenous plant protein extracts could not be detected. a**, **b**, assays were performed according to previously published methods [10,11]. Dopamine and secologanic acid (**a**) or secologanin (**b**) were added to desalted protein extracts from *A. salviifolium* (**a**) or *C. ipecacuanha* (**b**) young leaves. As controls, identical reactions were performed using boiled extracts. Samples were taken at indidicated time points and analyzed by UPLC-MS. Within two hours almost no secologanic acid or secologanin were consumed. Small amounts of the reaction products DAIIA/DAIA (**a**) or DAII/DAI (**b**) were observed but were higher in the boiled extract control than in the active extracts and thus be attributed to a non-enzymatic background reaction. **c**, **d**, positive controls for the detection of enzymatic activity in crude extracts confirms the presence of some active enzymes in the prepared protein extracts. **c**, detection of *O*-methyltransferase activity in desalted *A. salviifolium* extract. The same protein extract as in **a** was incubated with DAIIA **5a**/ DAIA **5b** mix and cofactors. Concomitant with consumption of DAIIA **5a** the reaction product 6-*O*-Me-DAIIA **6a** accumulated indicating enzymatic conversion.

**d**, consumption of DAII **4a** was concumitant with accumulation of DAIIA **5a** in the same *C. ipecacuanha* protein extract shown in **b** indicating enzymatic conversion. LC-MS peak areas are shown as bars of the mean of three replicates, error bars are standard error of the mean, dots are single data points.

**a**

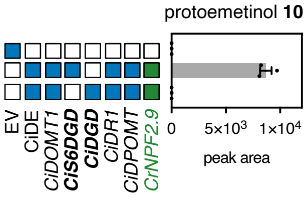


**b**

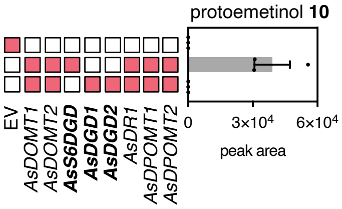


**Supplementary Fig. 9. Pathway reconstitution with CiDGD and AsDGD1,2 does not lead to protoemetine formation. a,** when *CiDGD*[12] is expressed with the indicated pathway genes, no protoemetinol is formed, confirming that this glucosidase is not on protoemetine pathway. **b**, similarly, when *AsDGD1*,*2* are expressed with the indicated pathway genes no protoemetinol is formed. LC-MS peak areas are shown as bars of the mean of three biological replicates, error bars are standard error of the mean, dots are single data points.

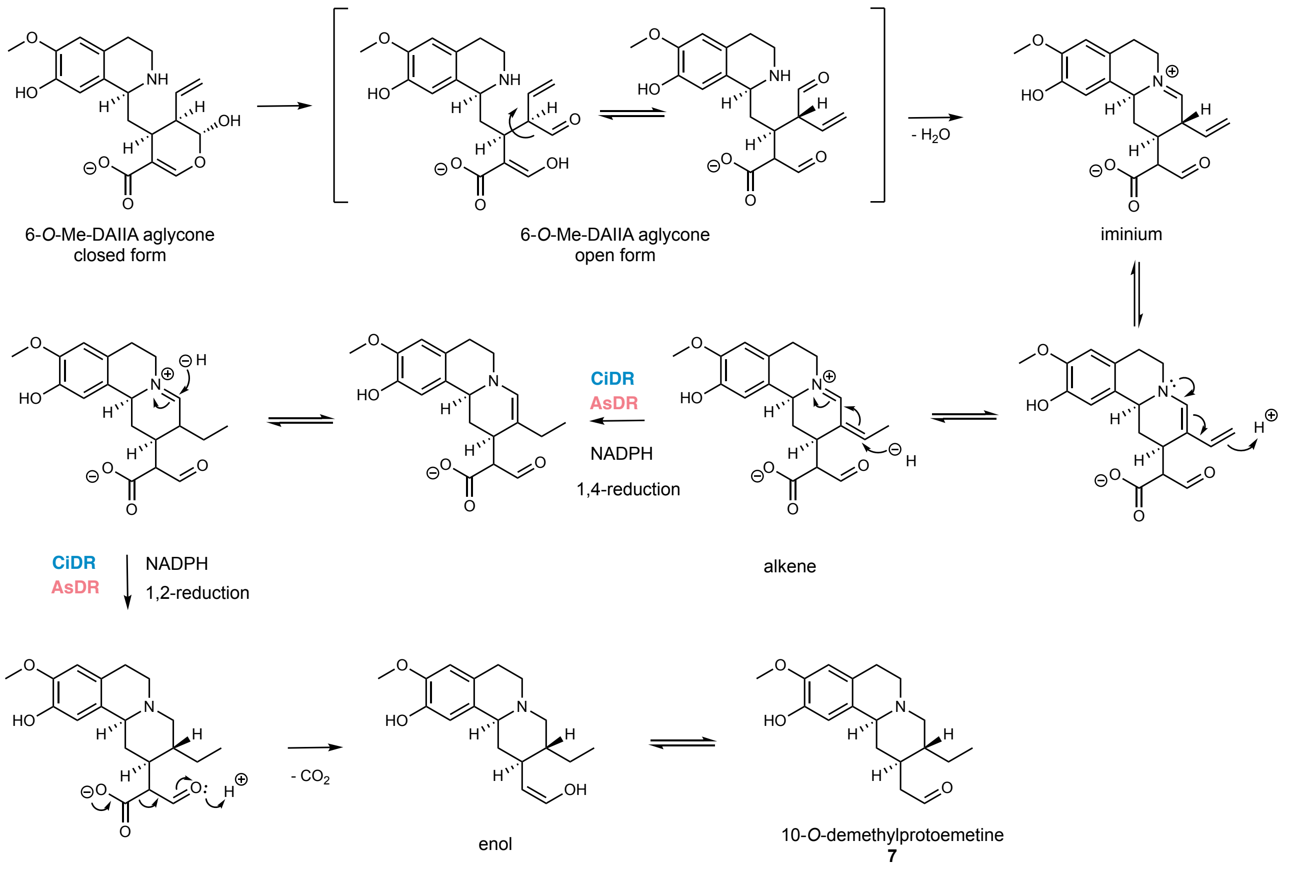


**Supplementary Fig. 10. Putative reduction mechanism of DRs and spontaneous decarboxylation.** 6-*O*-Me-DAIIA aglycone forms an iminium ion. After tautomerization, DR would first perform a 1,4 reduction followed by a 1,2 reduction. Spontaneous decarboxylation would then occur and lead to the enol which is in equilibrium with 10-*O*-demethylprotoemetine.

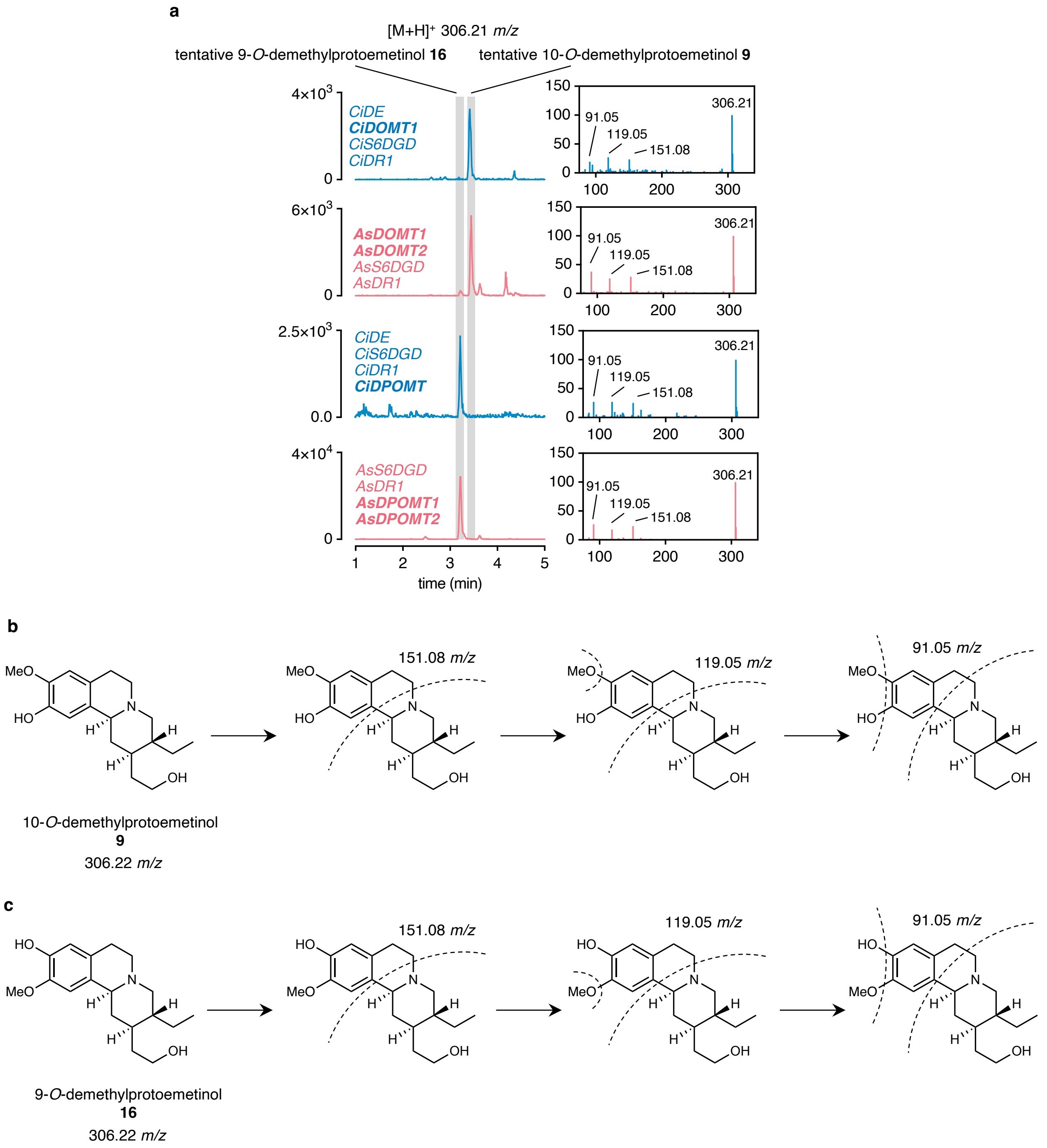


**Supplementary Fig. 11. Assignment of 10-*O*-demethylprotoemetinol and 9-*O*-demethylprotoemetinol.** **a**, LC-MS traces and MS2 data extracted from pathway reconstitution experiments shown in Fig. 3 and Extended Data Fig. 4 (*C. ipecacuanha*) and 6 (*A. salviifolium*). Two peaks with different retention times but identical $MS^1$ *m/z* and $MS^2$ fragments are detected when indicated pathway genes are expressed in combination. **b**, proposed fragmentation of 10-*O*-demethylprotoemetinol. **c**, proposed fragmentation of 9-*O*-demethylprotoemetinol. The peaks are proposed to correspond to 9-*O*-demethylprotoemetinol and 10-*O*-demethylprotoemetinol, respectively, based on the following observations: (a) $MS^1$ data are consistent with the theoretical *m/z*, (b) the observed $MS^2$ fragments are in agreement with predicted fragments, (c) $MS^2$ fragments show expected difference in *m/z* (-14) compared to protoemetinol fragments (*m/z* 165.09 in protoemetinol versus *m/z* 151.08 observed here), (d) different retention times depending on the specific combination of combinatorically expressed pathway genes are consistently observed and

identical for both species. Data for *C. ipecacuanha* pathway gene expression is shown in blue and labelled with *C.i.* as abbreviation; data for *A. salviifolium* pathway gene expression in magenta, abbreviated with *A.s.*; and data for the respective standards in black as indicated. $MS^2$ data is shown as relative abundance of ions with *m/z* values of the most abundant fragment ions indicated. The specific pathway genes expressed were as indicated. In bold the pathway genes that differed in the combinations leading to peaks with different retention times.

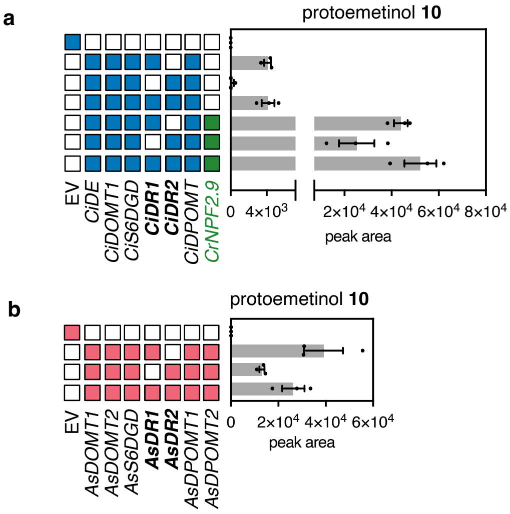


**Supplementary Fig. 12. Additional CiDR and AsDR paralogs have partial redundancy with DR1s. a,** *CiDR2* is a coexpressed *MDR* transcript with high sequence homology to *CiDR1* (see Extended Data Fig. 2a) and reconstitutes the pathway to protoemetinol, if the heterologous vacuolar exporter *CrNPF2.9* is co-overexpressed. However, in direct comparison to expression of *CiDR1*, less protoemetinol accumulates. **b**, *AsDR2* is a coexpressed *MDR* transcript with high sequence homology to *AsDR1* (see Extended Data Fig. 2b) and reconstitutes the pathway to protoemetinol, although less protoemetinol accumulates in direct comparison to expression of *AsDR1*. LC-MS peak areas are shown as bars of the mean of three biological replicates, error bars are standard error of the mean, dots are single data points.

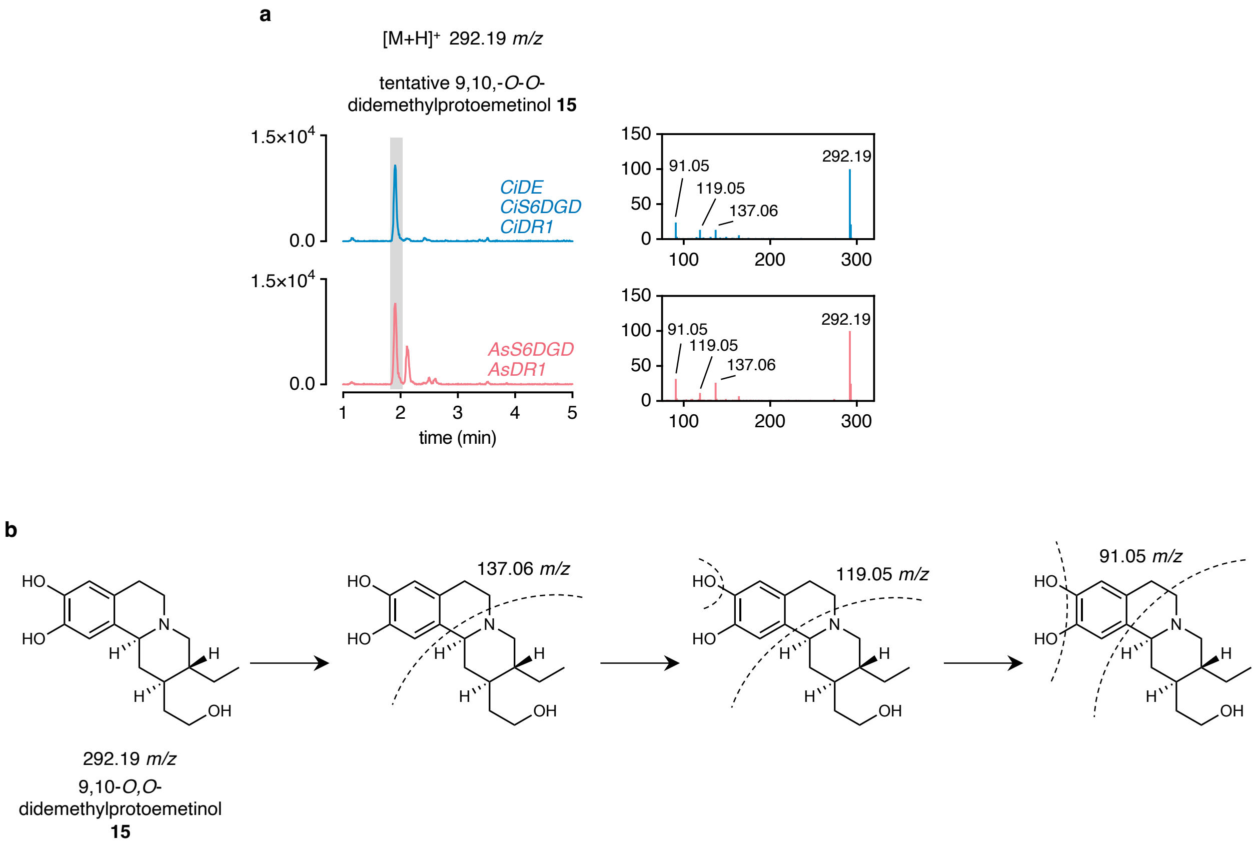


**Supplementary Fig. 13. Assignment of 9,10-*O*,*O*-didemethylprotoemetinol. a**, LC-MS traces and MS$^2$ data extracted from pathway reconstitution experiments shown in Fig. 3 and Extended Data Fig. 4 (*C. ipecacuanha*) and 6 (*A. salviifolium*). Upon expression of the indicated pathway genes, one peak with the same retention time and identical MS$^1$ *m/z* and MS$^2$ fragments was detected. Upon expression of *A. salviifolium* pathway genes only, an additional peak with different retention time but same *m/z* appeared which may be a different stereoisomer. **b**, fragmentation of 9,10-*O,O*-didemethylprotoemetinol. This peak is proposed to be 9,10-*O,O*-didemethylprotoemetinol based on the following observations: (a) MS$^1$ data are consistent with the theoretical *m/z*, (b) the observed MS$^2$ fragments are in agreement with predicted fragments, (c) MS$^2$ fragments show expected difference (-28 and -14, respectively) in *m/z* compared to protoemetinol and proposed 9- and 10-*O*-demethylprotoemetinol fragments (*m/z* 165.09 in protoemetinol versus *m/z* 137.06 observed here), (d) appearance of peaks in specific combinations of expressed pathway genes is as expected. *C. ipecacuanha* data is shown in blue and labelled with *C.i.* as abbreviation; *A. salviifolium* data in magenta, abbreviated with *A.s.*; and data for the respective standards in black as indicated. MS$^2$ data is shown as relative abundance of ions with *m/z* values of the most abundant fragment ions indicated.

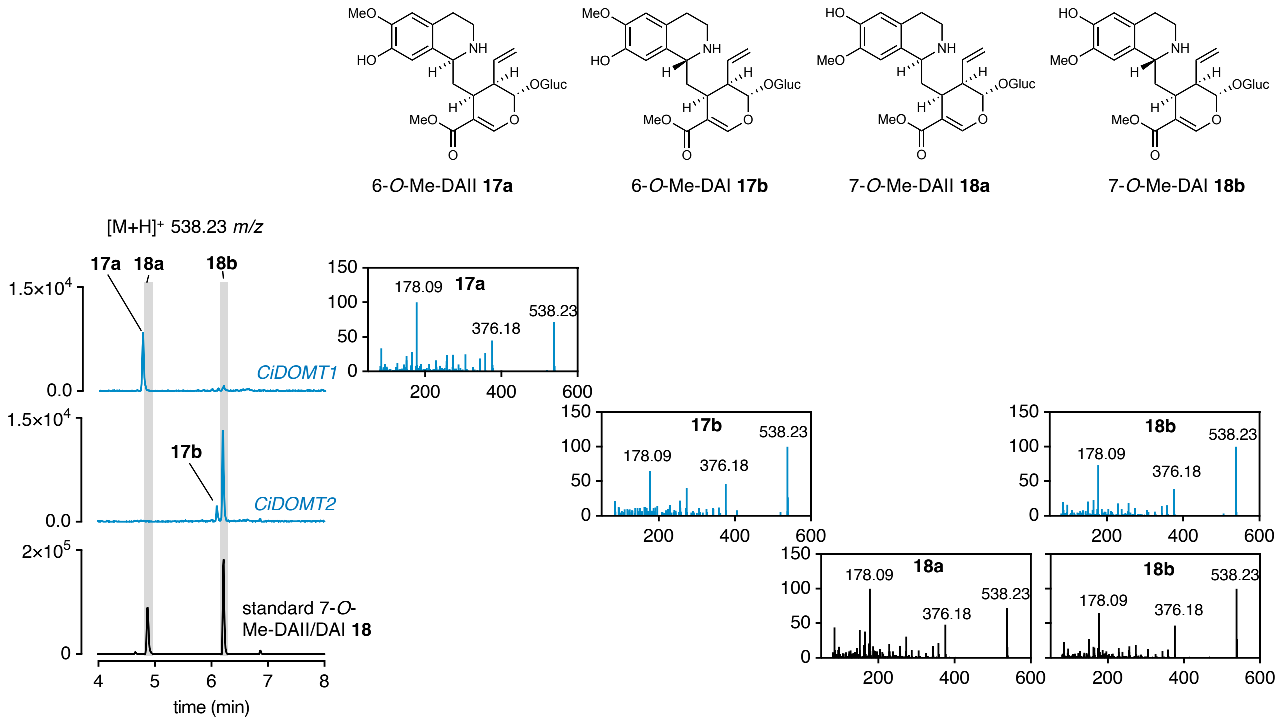


**Supplementary Fig. 14. Extracted ion chromatograms and MS$^2$ data of 7-*O*-Me-DAI/I and putative 6-*O*-Me-DAI/I from *N. benthamiana* agroinfiltrations and standards.** On the left EICs for 538.23 *m/z* of *N. benthamiana* expressing *CiDOMT1* (top), *CiDOMT2* (center) compared to 7-*O*-Me-DAI/I standard (bottom, black, produced through chemical Pictet-Spengler reaction of 4-*O*-Me-dopamine with secologanin). Based on results from derivatives and the observed ratio of peak intensity in the standard, it can be assumed that the first peak is the *S* epimer and the second one the *R* epimer. 6-*O*-Me-DAII/I are tentatively identified based on MS$^2$ fragmentation and peak shifts. Minor peaks in the standard with the same *m/z* values are consistently observed when performing these reactions and could correspond to "neo" isomers which were previously reported [9].

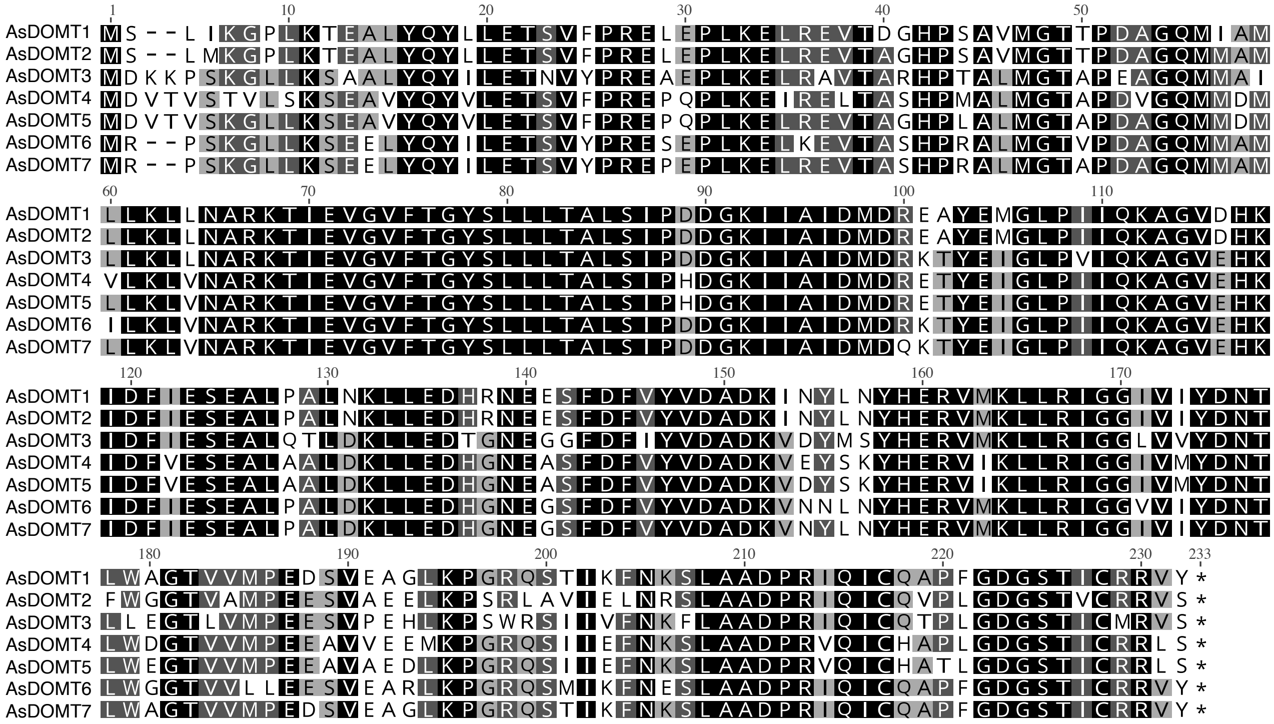

**Supplementary Fig. 15. AsDOMT amino acid alignment.** MUSCLE alignment. Colour code depicts identical amino acid residues in 100 % of the sequences (black), 80-100% (dark grey), 60-80% (light grey), and less than 60 % (white).

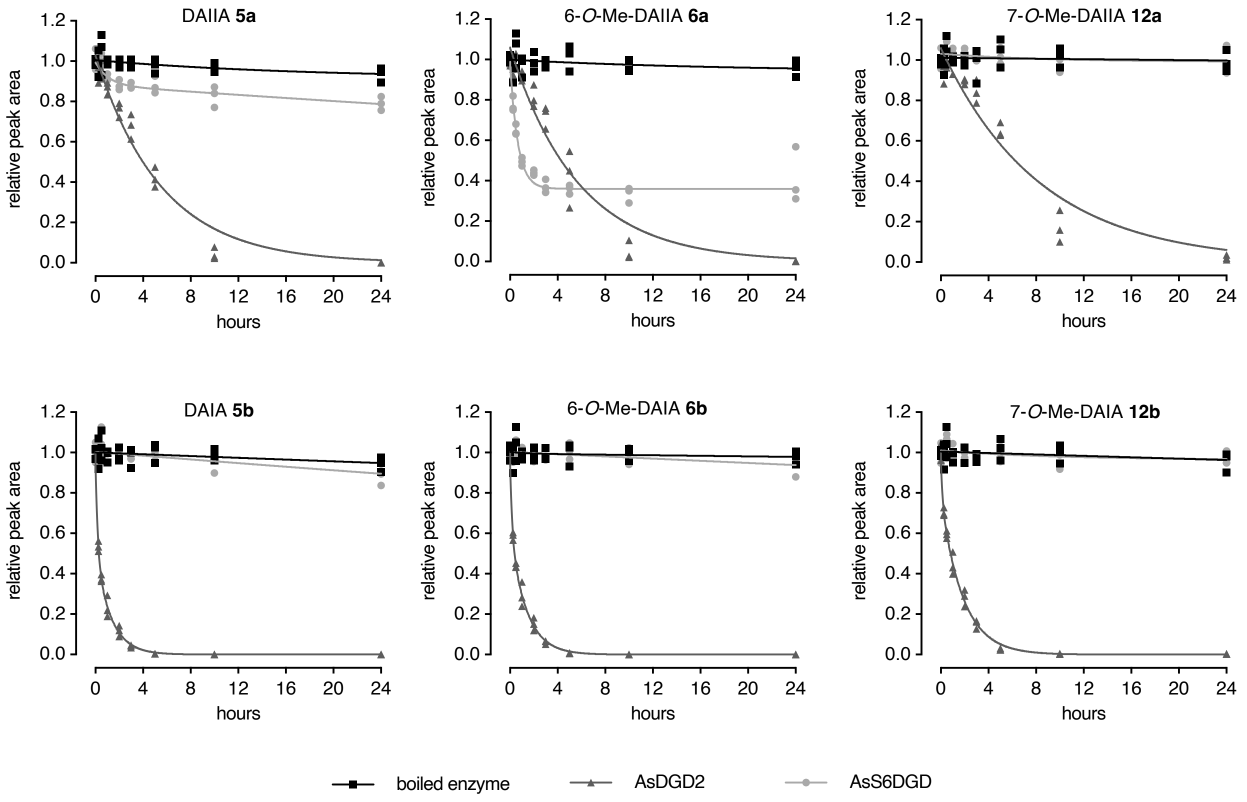


**Supplementary Fig. 16. *In vitro* substrate competition assays of recombinant *A. salviifolium* glucosidases.** Recombinant AsDGD2 and AsS6DGD (0.5 µM) or, as control, a boiled AsDGD2/AsS6DGD mix, were each assayed in triplicate with a substrate mix containing six *A. salviifolium*-specific ipecac alkaloid glucosides at saturating concentrations (100 µM each). The first time point sample was taken at the assay start immediately after pipetting on ice. Subsequent time point samples were taken upon incubation at 30°C for 15 min, 30 min, 1 h, 2h, 3h, 5h, 10h and 24 h. The highly reactive aglycone reaction products were not detected, so instead consumption of each substrate was monitored. Peak areas are normalized to peak areas at start of assay. AsDGD2 consumes all substrates within 24 hours whereas AsS6DGD specifically consumes 6-*O*-Me-DAIIA and a minor fraction of DAIIA. Substrates were stable in boiled enzyme controls over 24 hours.

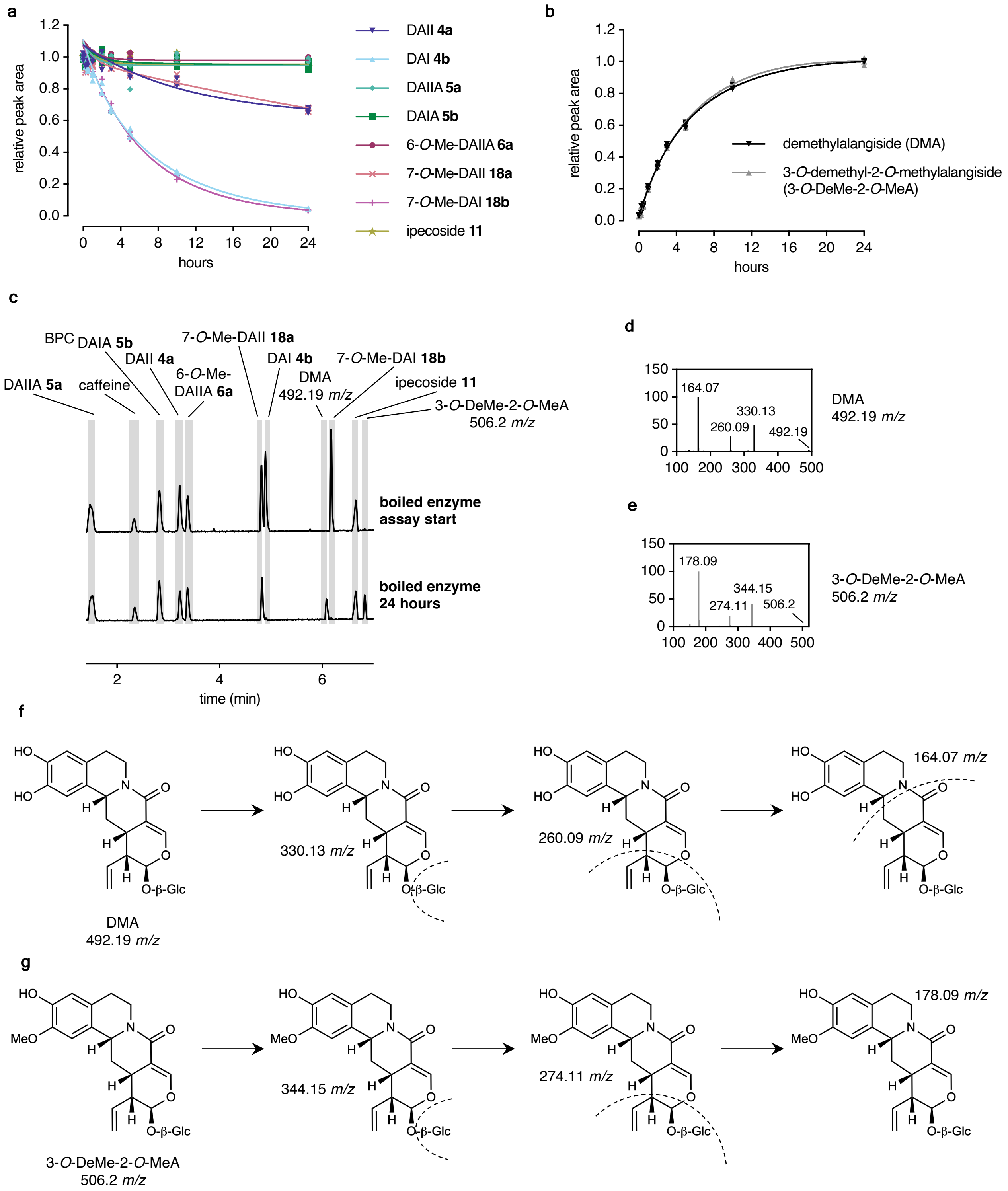


**Supplementary Fig. 17. Non-enzymatic background reactions of *C. ipecacuanha* specific ipecac alkaloid glucosides.** Since many of the methyl ester substrates used by *C. ipecacuanha* undergo rapid lactamization (in contrast to the acid substrates used for *A.* ***salviifolium***, which do not lactamize) we measured the background rate of disappearance of the substrates for normalization of apparent enzymatic rates. The figure depicts non-enzymatic background reactions taken into account when assaying the enzymatic activity (Supplementary Fig. 26). Boiled CiDGD/CiS6DGD enzyme mix was incubated in triplicates with mixed *C. ipecacuanha*-specific ipecac alkaloid glucosides over the course of 24 hours. All glucosides were detected in the *C. ipecacuanha* metabolite tissue analysis in this study except 7-*O*-Me-DAII **18a** and 7-*O*-Me-DAI **18b.** However, the latter was shown to be

produced by CiDOMT2 in this study and a previous study [13] and the epimeric mixture was therefore included in the substrate mix. The first time point sample was taken at the assay start immediately after pipetting on ice. Subsequent time point samples were taken upon incubation at 30°C for 15 min, 30 min, 1 h, 2h, 3h, 5h, 10h and 24 h. **a**, Analysis of the boiled enzyme controls reveal that acids are stable but methyl ester ipecac alkaloids (except ipecoside) decrease over the course of 24 hours. Around 80% of the *S*-epimers DAII **4a** and 7-*O*-Me-DAII **18a** were still present after 24 hours, however the *R*-epimers DAI **4b** and 7-*O*-Me-DAI **18b** were completely depleted within 24 hours. Peak areas are normalized to peak areas at start of assay. **b**, Concomitant with reduction of DAI **4b** and 7-*O*-Me-DAI **18b** an increase in their lactamized forms demethylalangiside (DMA) and 3-*O*-demethyl-2-*O*-methylalangiside (3-*O*-DMe-2-*O*-Me-A) was observed (peak areas normalized to peak areas at 24 hours for each compound). Assays were performed at pH 7.5 which is expected to induce spontaneous non-enzymatic lactamization of DAI and 7-*O*-Me-DAI. **c**, Representative base peak chromatograms (BPCs) depicting all compounds at start of reaction compared to 24 hours. **d**, $MS^2$ fragmentation of assigned DMA. **e**, $MS^2$ fragmentation of assigned 3-*O*-DMe-2-*O*-Me-A. **f**, Proposed fragmentation of DMA. **g**, Proposed fragmentation of 3-*O*-DMe-2-*O*-Me-A.

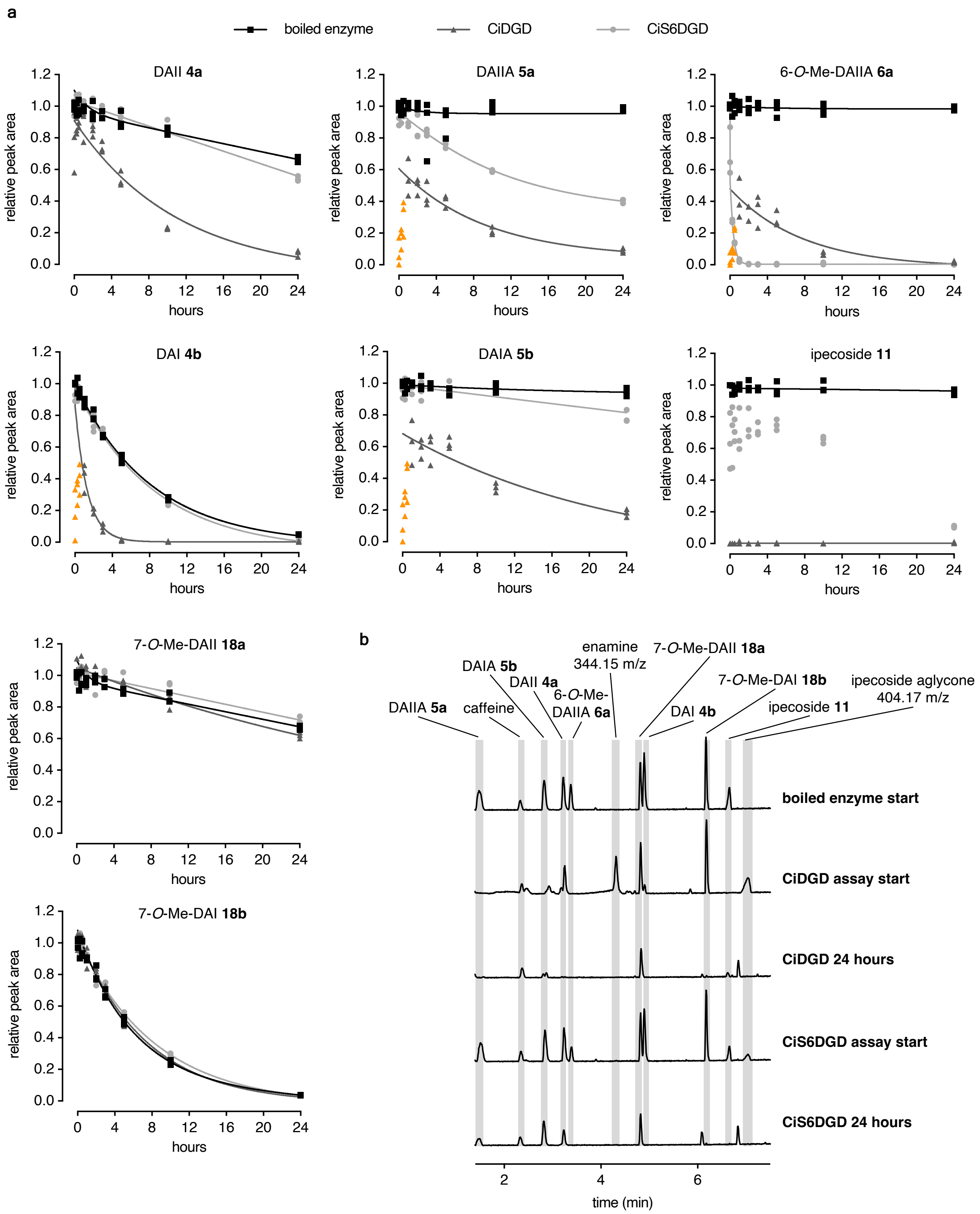


**Supplementary Fig. 18. *In vitro* substrate competition assays of recombinant *C. ipecacuanha* glucosidases.** Recombinant CiDGD and CiS6DGD (0.5 µM) or, as control, a boiled CiDGD/CiS6DGD mix, were each assayed in triplicate with a substrate mix containing eight *C. ipecacuanha*-specific ipecac alkaloid glucosides at saturating concentrations (100 µM each). The first time point sample was taken at the assay start immediately after pipetting on ice. Subsequent time point samples were taken upon incubation at 30°C for 15 min, 30 min, 1 h, 2h, 3h, 5h, 10h and 24 h. **a**, Relative peak areas of indicated ipecac glucosides over 24 hours normalized to peak areas of boiled enzyme control at start of assay. Note that in the reactions of CiDGD with DAI **4b**, DAIIA **5a**, DAIA **5b** and 6-*O*-Me-DAIIA **6a** we observed high levels of noise in the early time points in all replicates,

presumably due to the reactivity of these substrates. The orange-colored values were not considered for the displayed curve fittings for substrate consumption but are shown for full transparency. Despite this noise in the early time points, it is clear that CiS6DGD prefers substrate **6a**, as indicated in the leaf disk assays. Further, ipecoside **11** was already depleted in CiDGD samples from the assay start on indicating that the 30 s on ice until snap freezing were sufficient for CiDGD to fully deglycosylate ipecoside. In the reaction with CiS6DGD, ipecoside showed inconsistent peak area values over the whole time-course and therefore curve fitting was not possible. **b**, Selected base peak chromatograms (BPCs) reveal the presence of peaks consistent with ipecoside aglycone (404.17 *m/z*) and enamine (derived from DAII/DAI aglycone, 344.15 *m/z*) at the assay start. Both have been previously reported to occur in *in vitro* assays with recombinant CiDGD [12]. These peaks were not detected in samples from later time points, indicating that these aglycones are unstable.

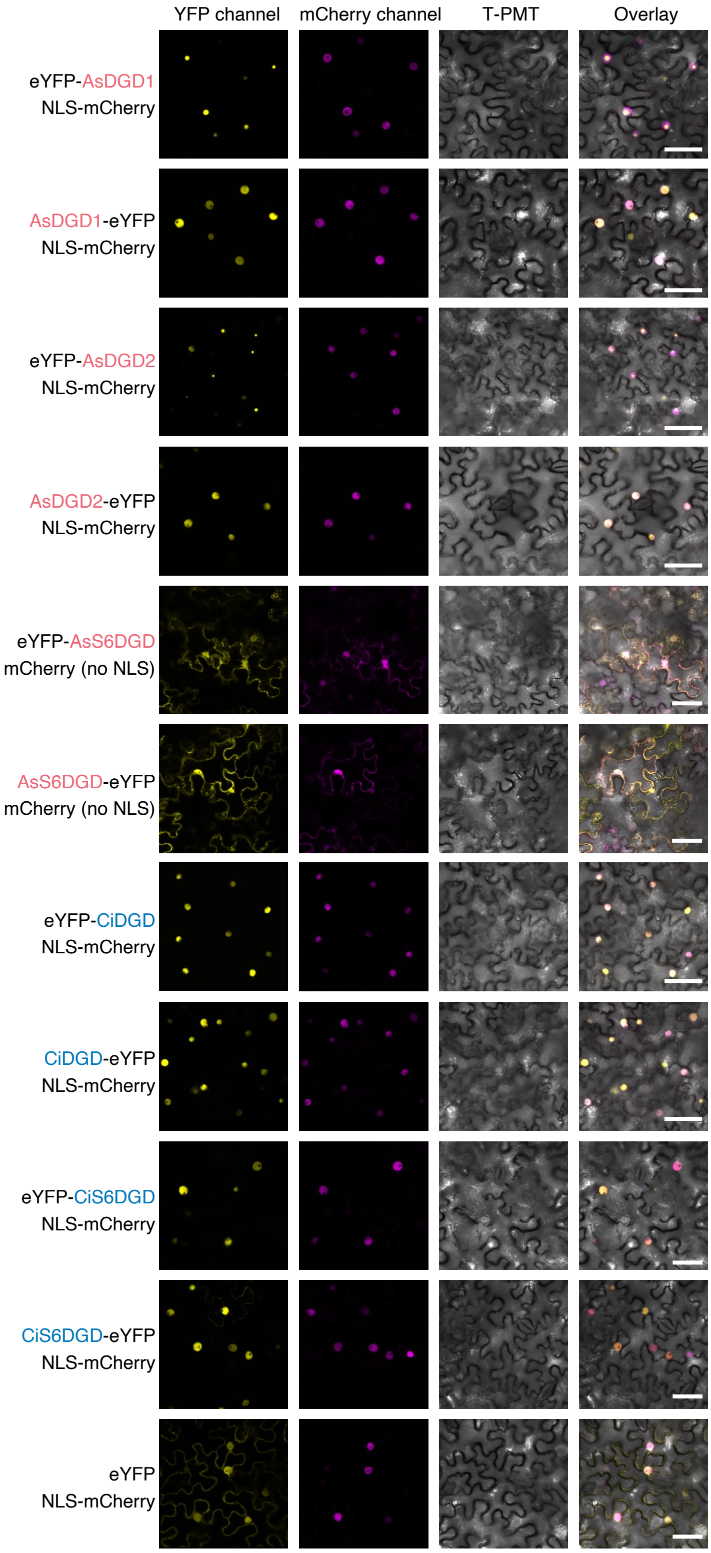


**Supplementary Fig. 19. Confocal laser scanning microscopy of glucosidases fused to eYFP.** AsDGD1,2 localize to the nucleus as *N*-terminal or *C*-terminal fusion proteins (row 1-4). When eYFP is fused to the *N*-terminus of AsDGDs the signal appears as a small particle within the nucleus (row 1 and 3 compared to 2 and 4). AsS6DGD shows localization signals in both nucleus and cytosol. Signals of CiDGD and CiS6DGD appear in the nucleus and appear similar when eYFP is fused to either the *N*- or *C*-terminus (row 7-10). mCherry is used either fused to NLS as nucleus marker or

without tag as a marker for both cytosol and nucleus (only in combination with AsS6DGD). Additional replicates are shown in Supplementary Fig. 29. Scale bars indicate 50 µm.

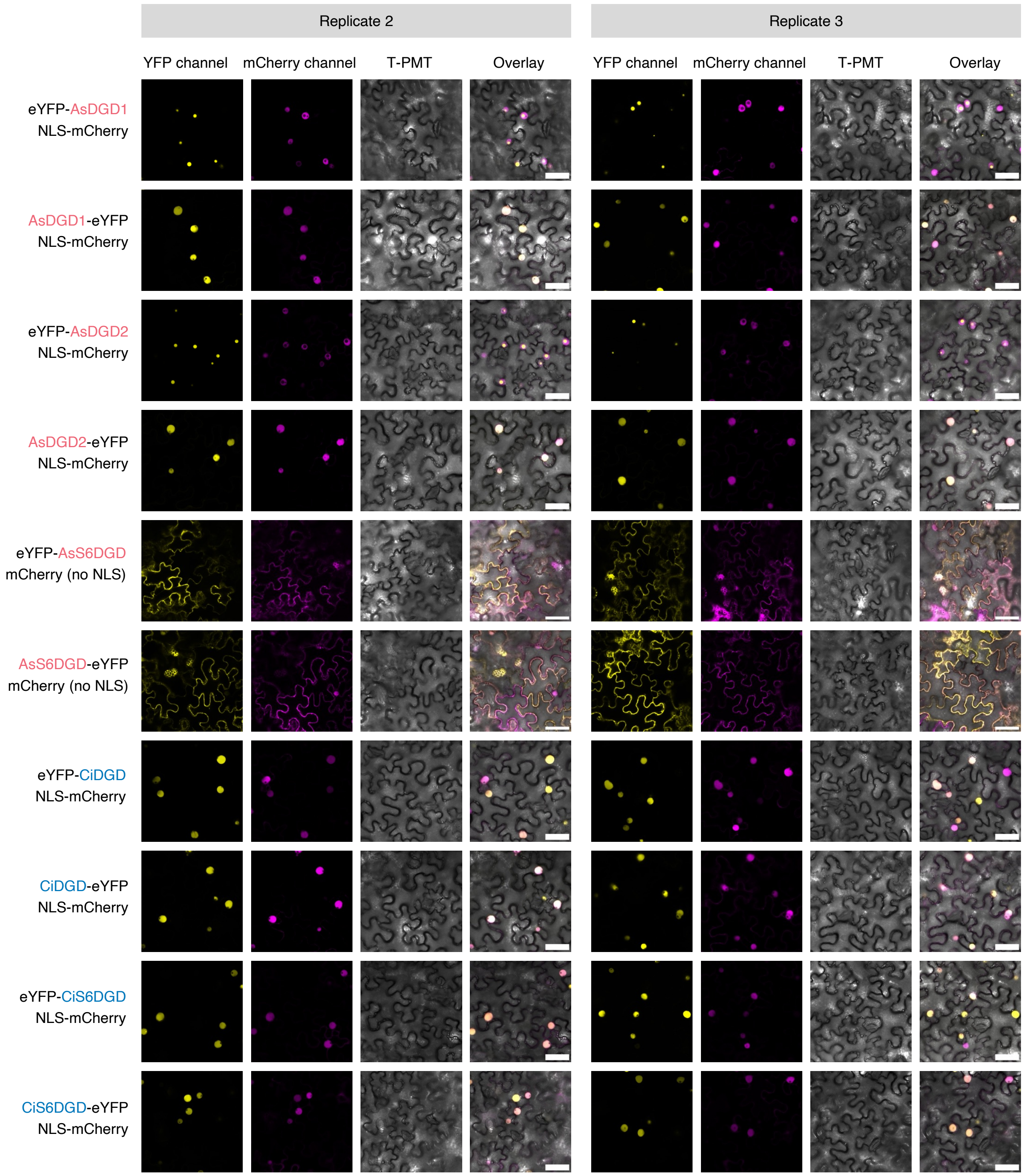


**Supplementary Fig. 20. Confocal laser scanning microscopy showing additional replicates of glucosidases fused to eYFP.** Data from two additional replicates shows that localization of glucosidases is highly consistent. Scale bars indicate 50 µm.

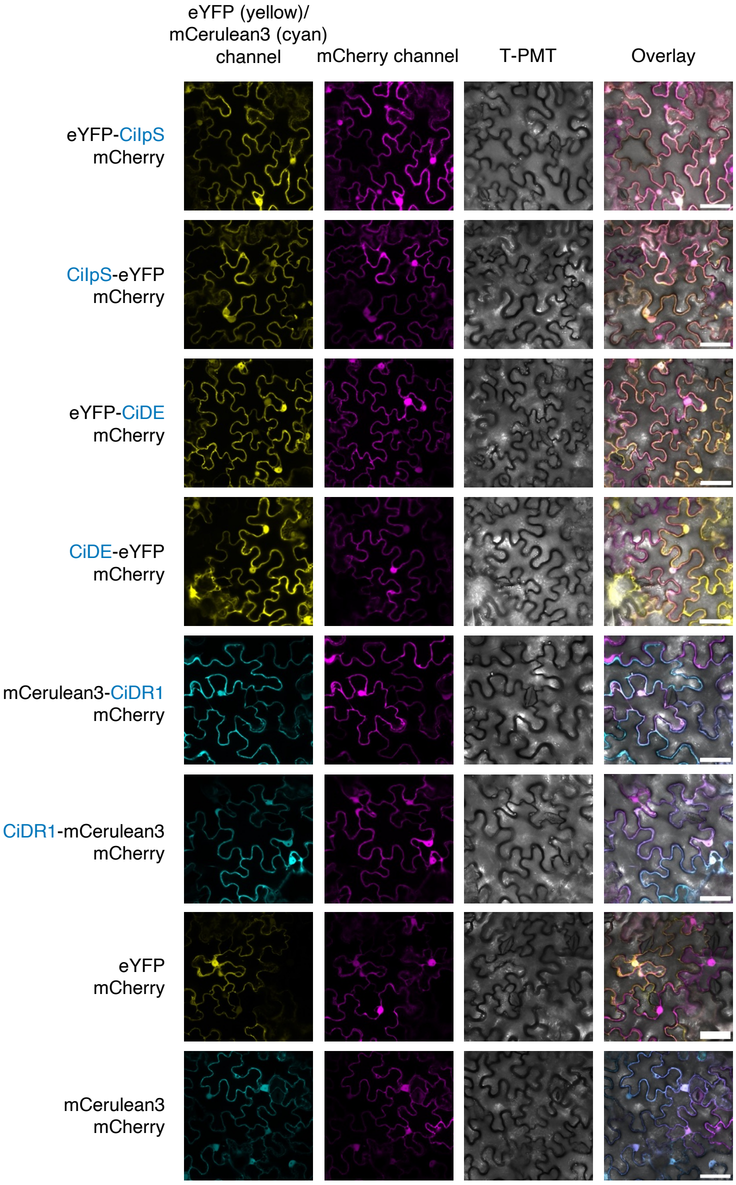


**Supplementary Fig. 21. Confocal laser scanning microscopy of CiIpS, CiDE and CiDR1 fused to eYFP or mCerulean3.** All three enzymes localize to the cytosol (and partially to the nucleus) when N-or C-terminally fused to eYFP or mCerulean3 (CiDR1 only). mCherry was used here without tag as a marker for both cytosol and nucleus. Scale bars indicate 50 µm. Experiments were repeated twice showing the same localization pattern.

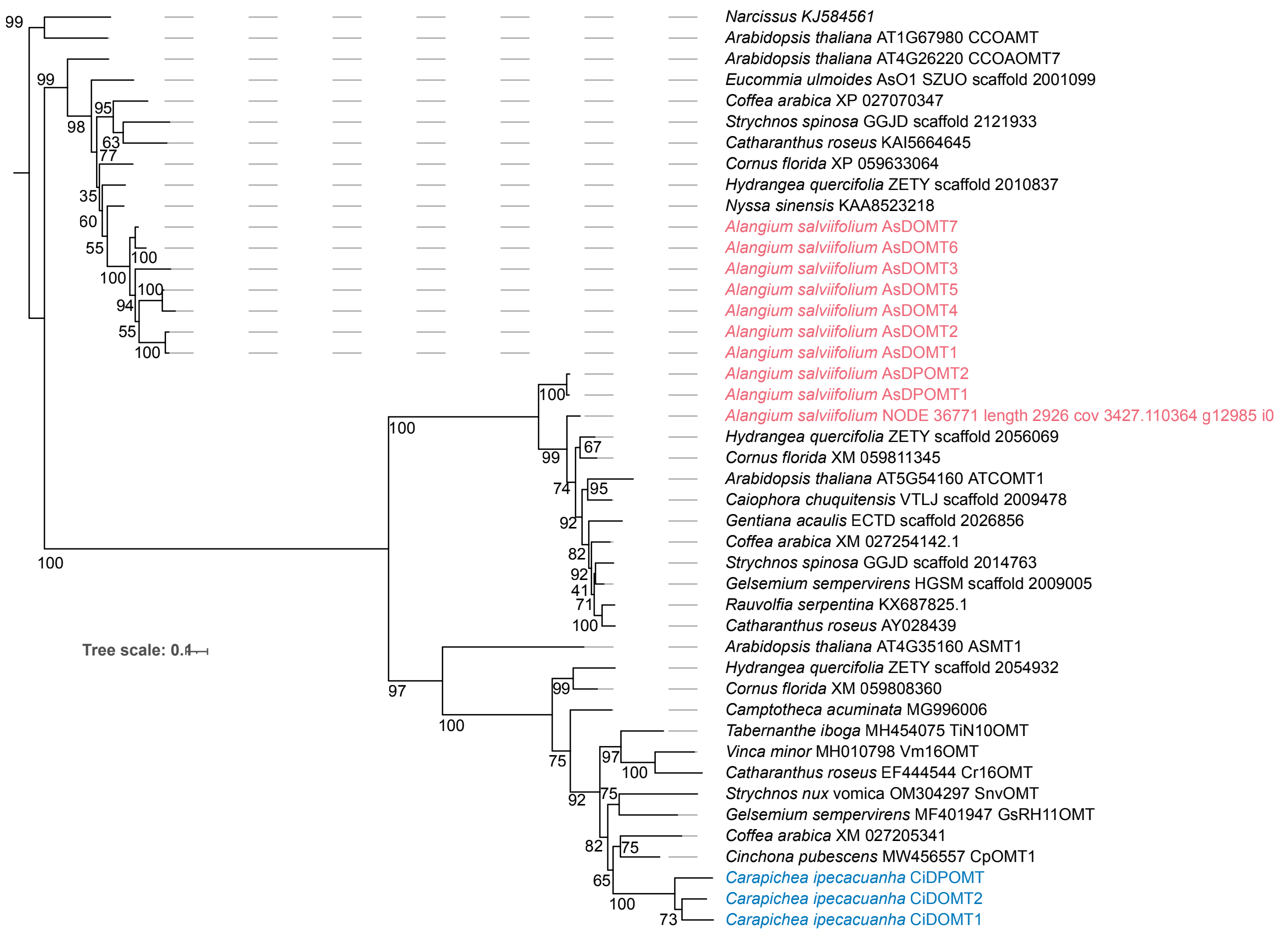


**Supplementary Fig. 22. Maximum likelihood tree of *A. salviifolium* versus *C. ipecacuanha* OMTs.** Tree from Fig. 6a shown here with all bootstrap values.

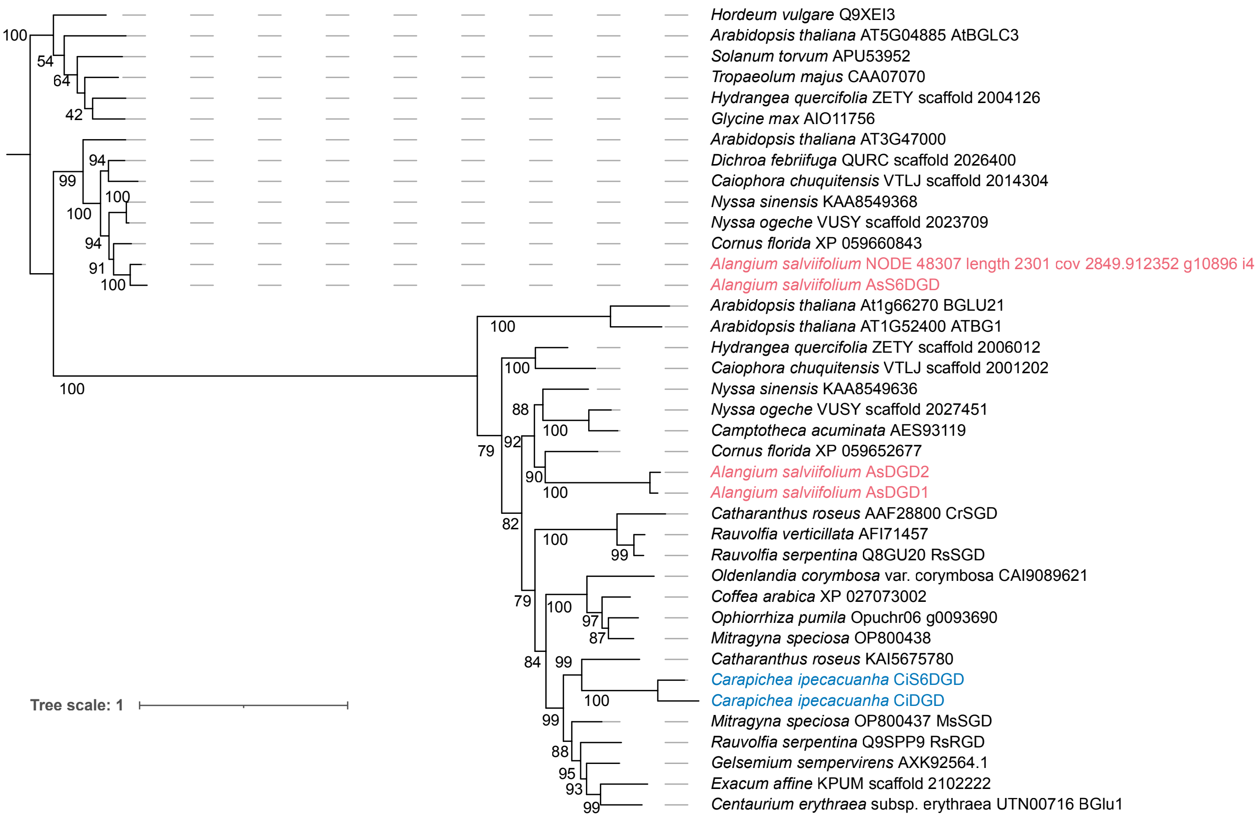


**Supplementary Fig. 23. Maximum likelihood tree of *A. salviifolium* versus *C. ipecacuanha* glucosidases.** Tree from Fig. 6b shown here with all bootstrap values

**Supplementary Fig. 24. NMR data for deacetylisoipecoside.**

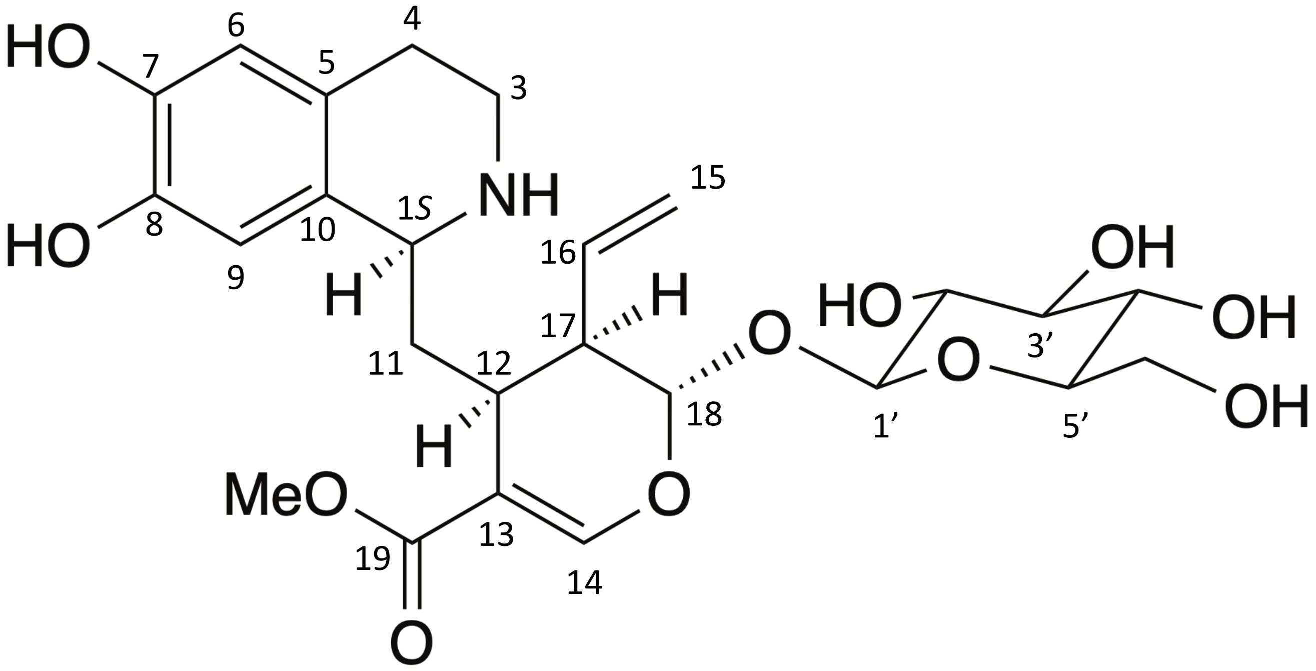


The numbering system is based on a previously published paper [9].

**NMR spectra for deacetylisoipecoside**

presaturated $^{1}$H NMR full range in MeOH-$d_3$ + 0.1% formic acid (FA)

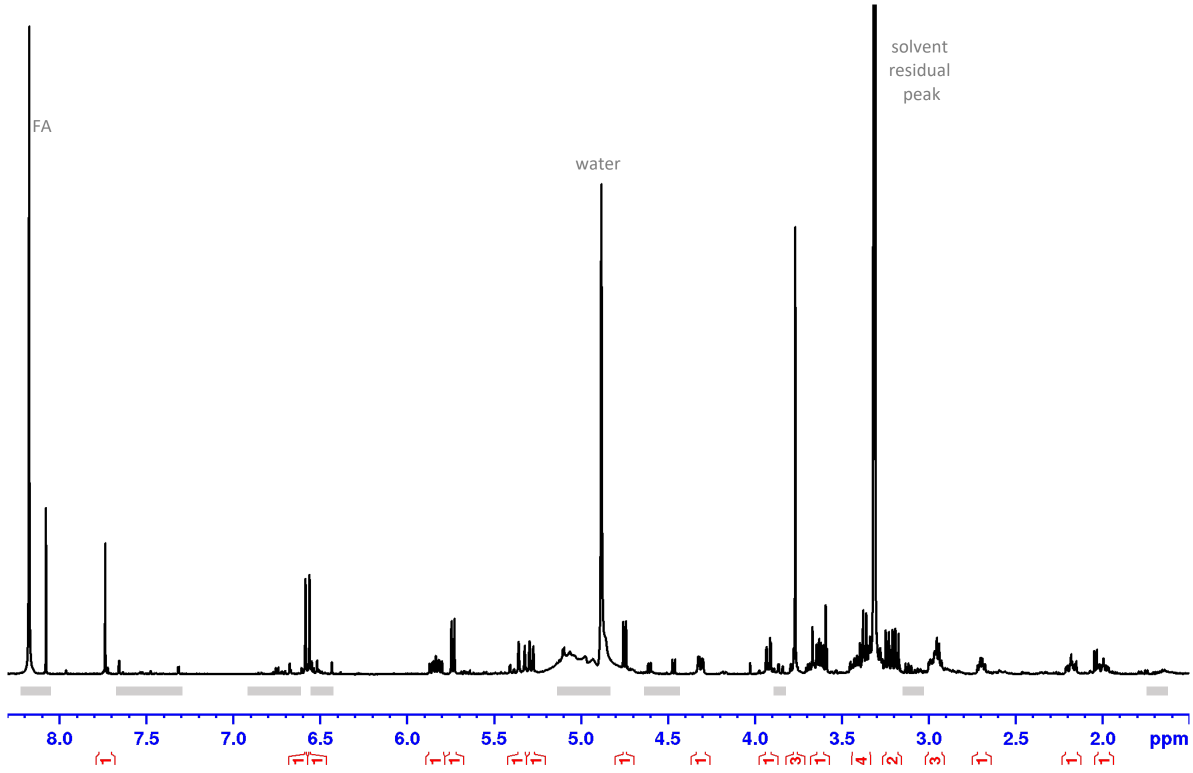


Shaded areas mark impurity and solvent

DEPTQ full range in MeOH-$d_3$ + 0.1% FA

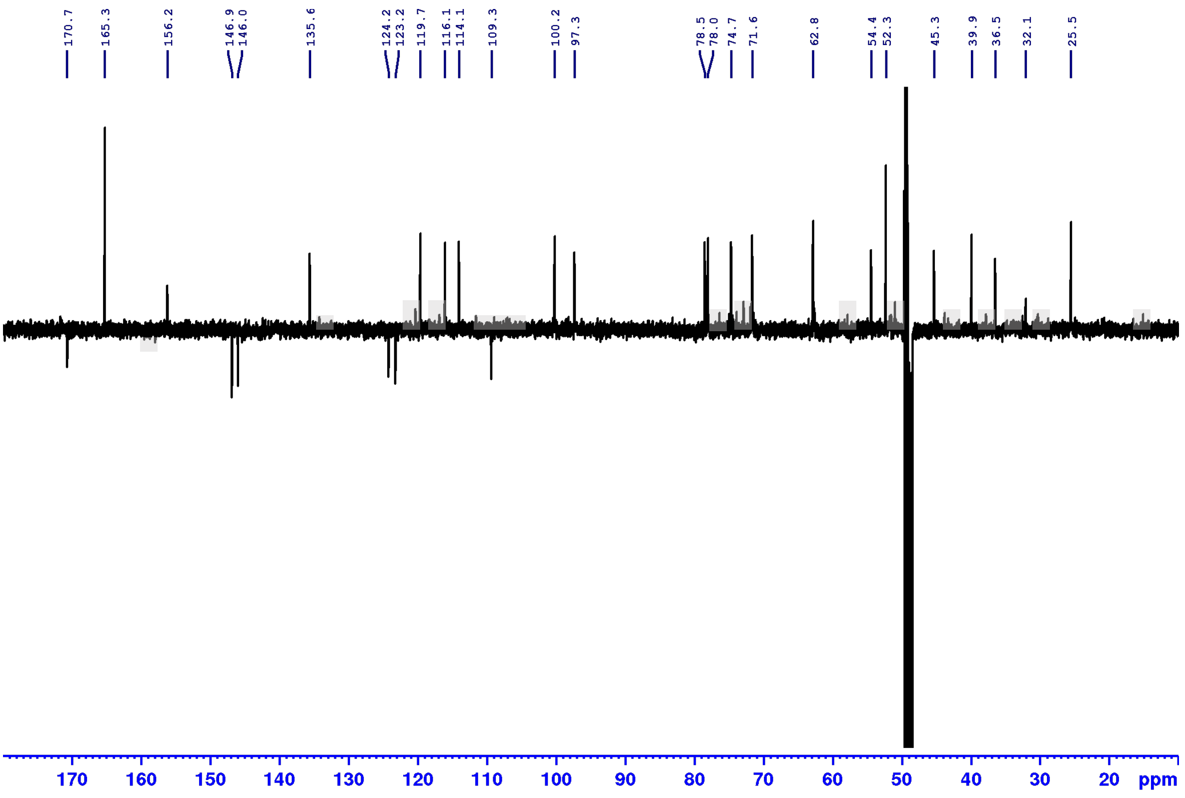


Shaded areas mark impurity and solvent

**NMR spectra for deacetylisoipecoside**

Phase sensitive HSQC, full range in MeOH-$d_3$ + 0.1% FA

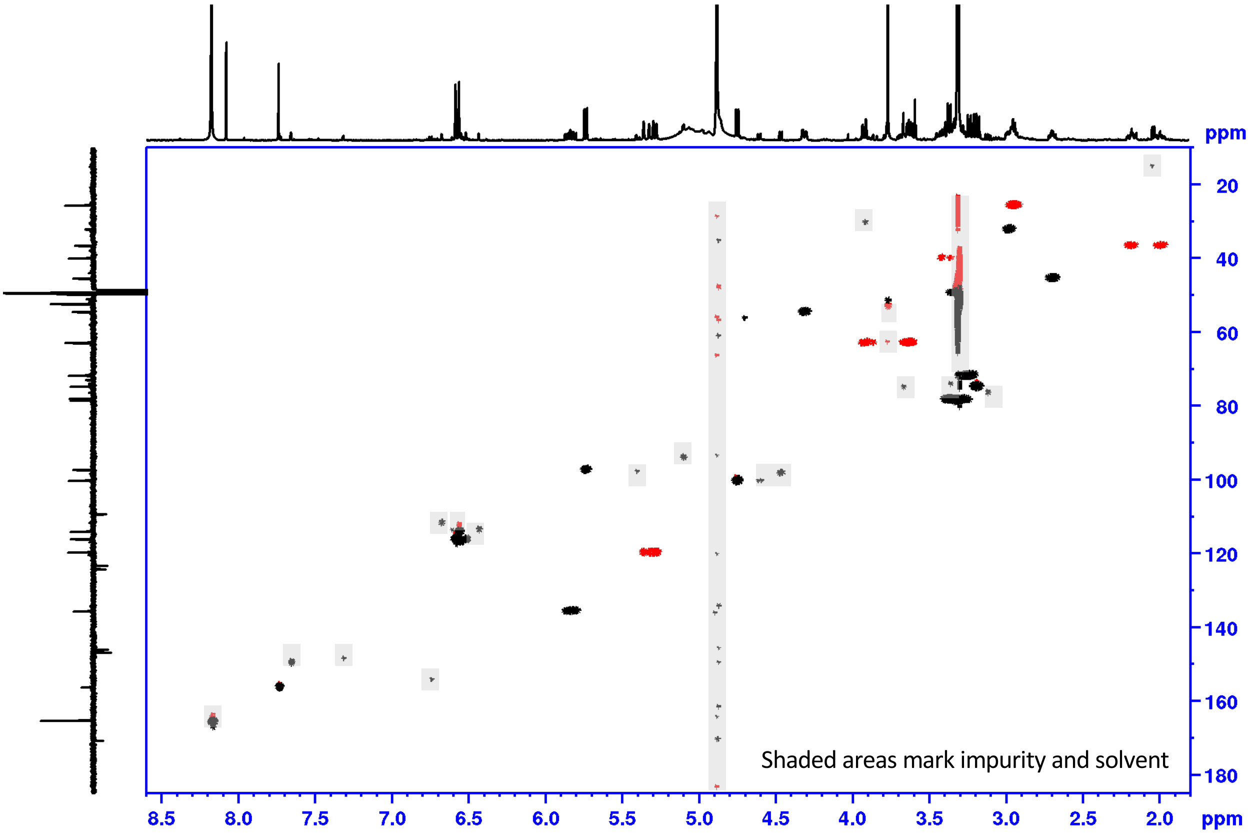


HMBC full range in MeOH-$d_3$ + 0.1% FA

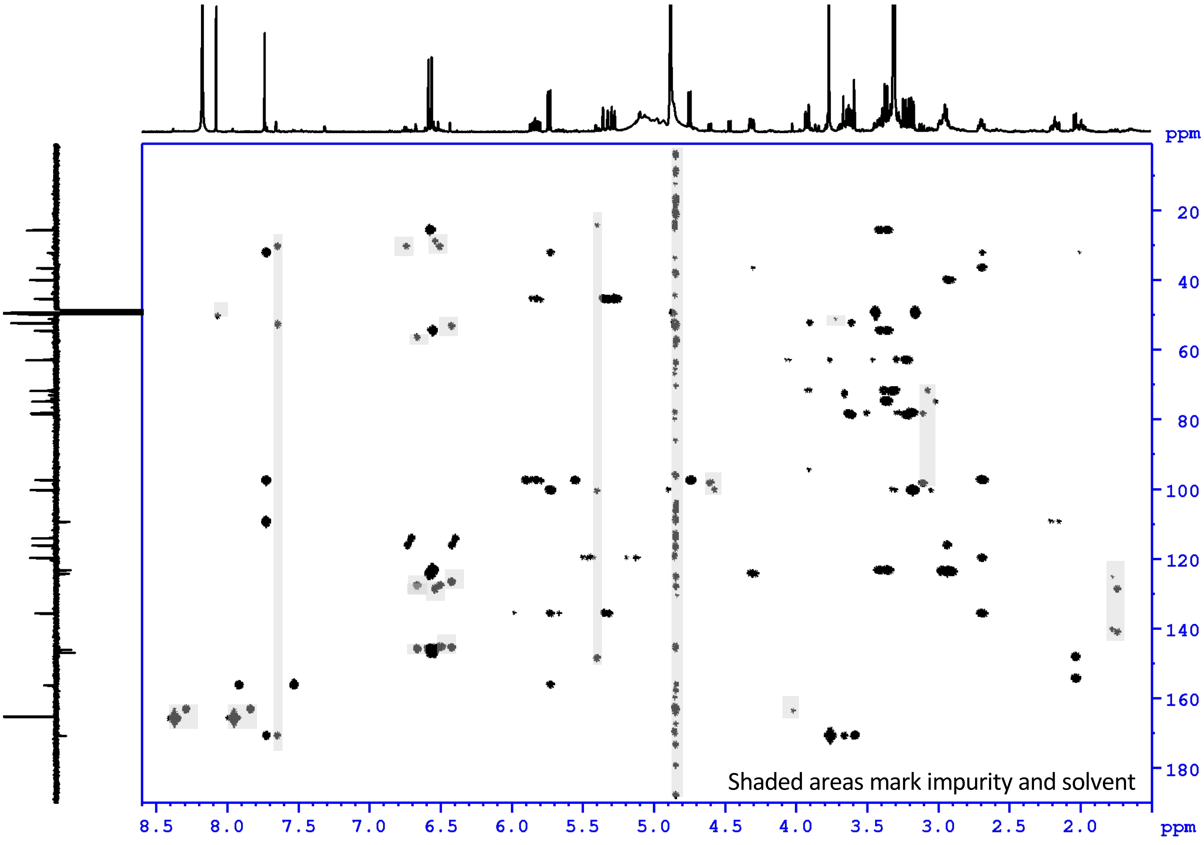

**NMR spectra for deacetylisoipecoside**

COSY, full range in MeOH-$d_3$ + 0.1% FA

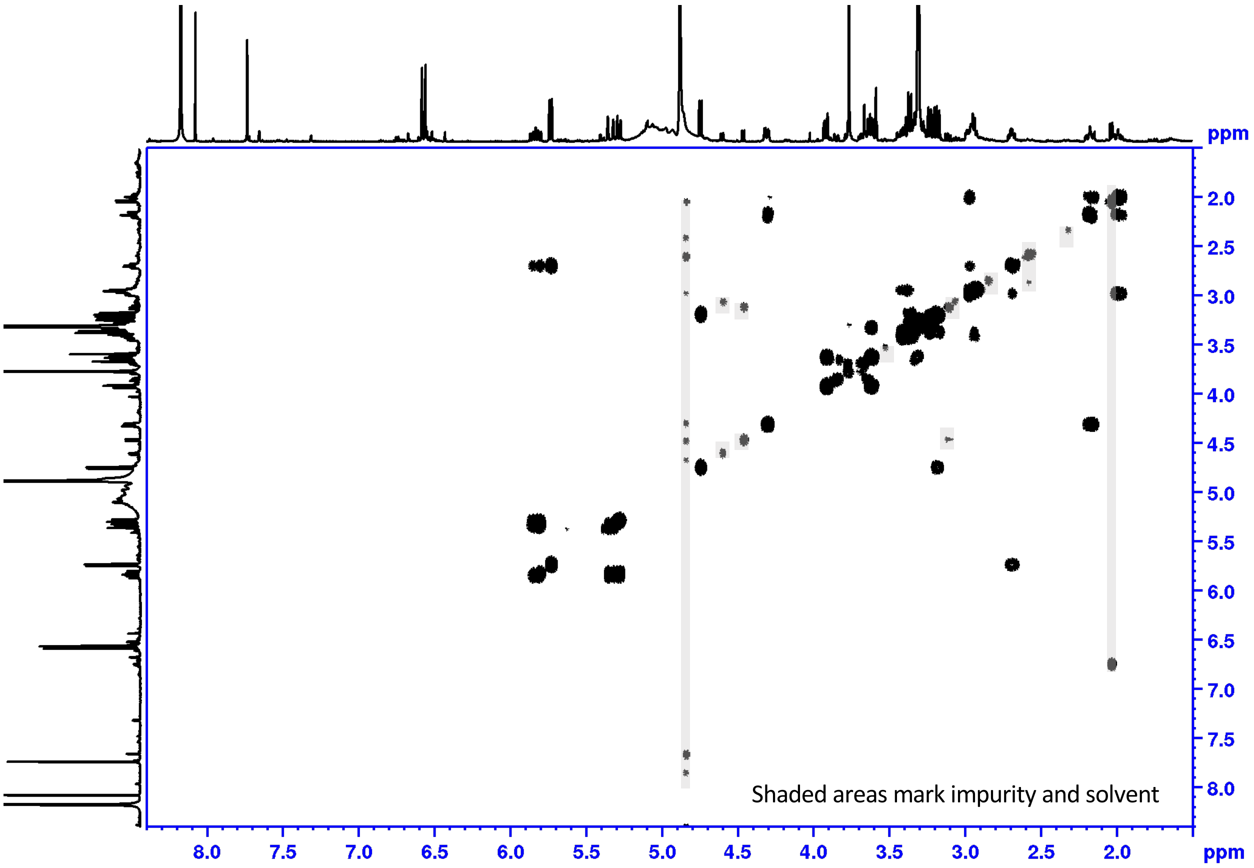


ROESY full range in MeOH-$d_3$ + 0.1% FA

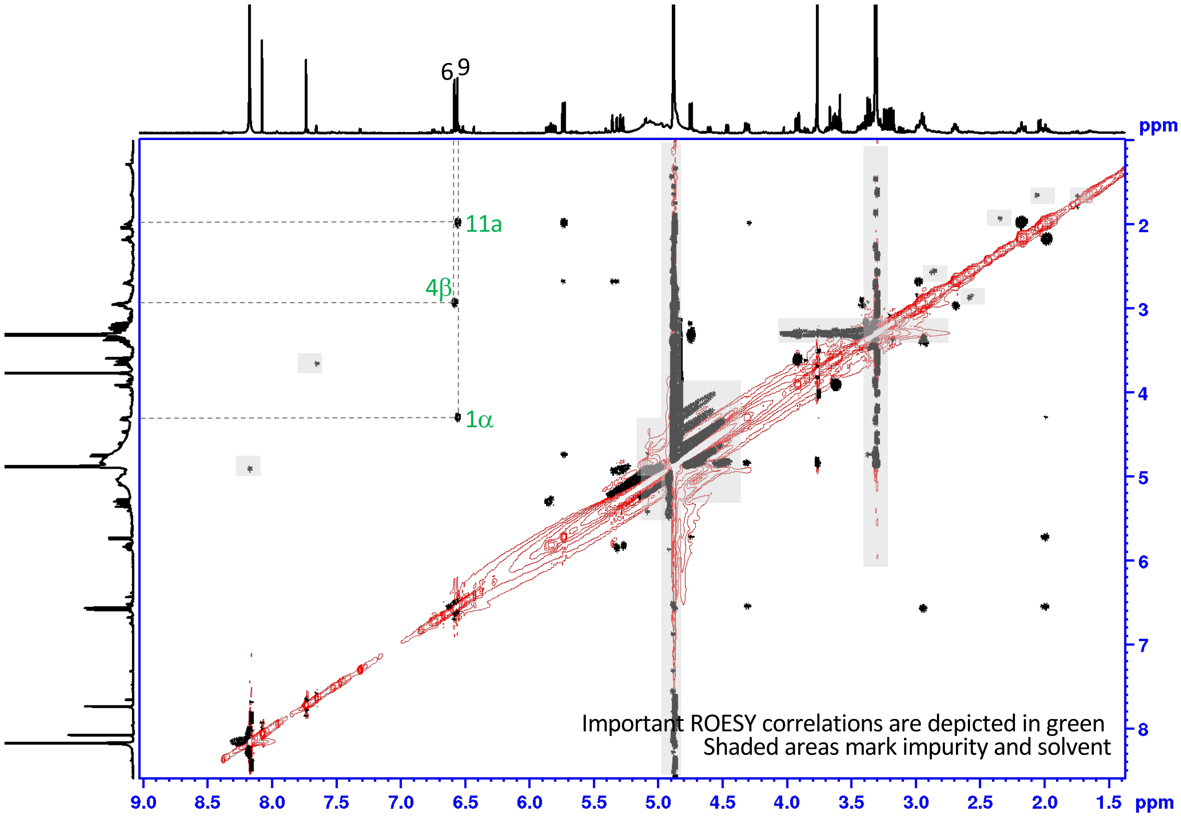

## NMR spectra for deacetylisoipecoside

ROESY aliphatic range in MeOH-$d_3$ + 0.1% FA

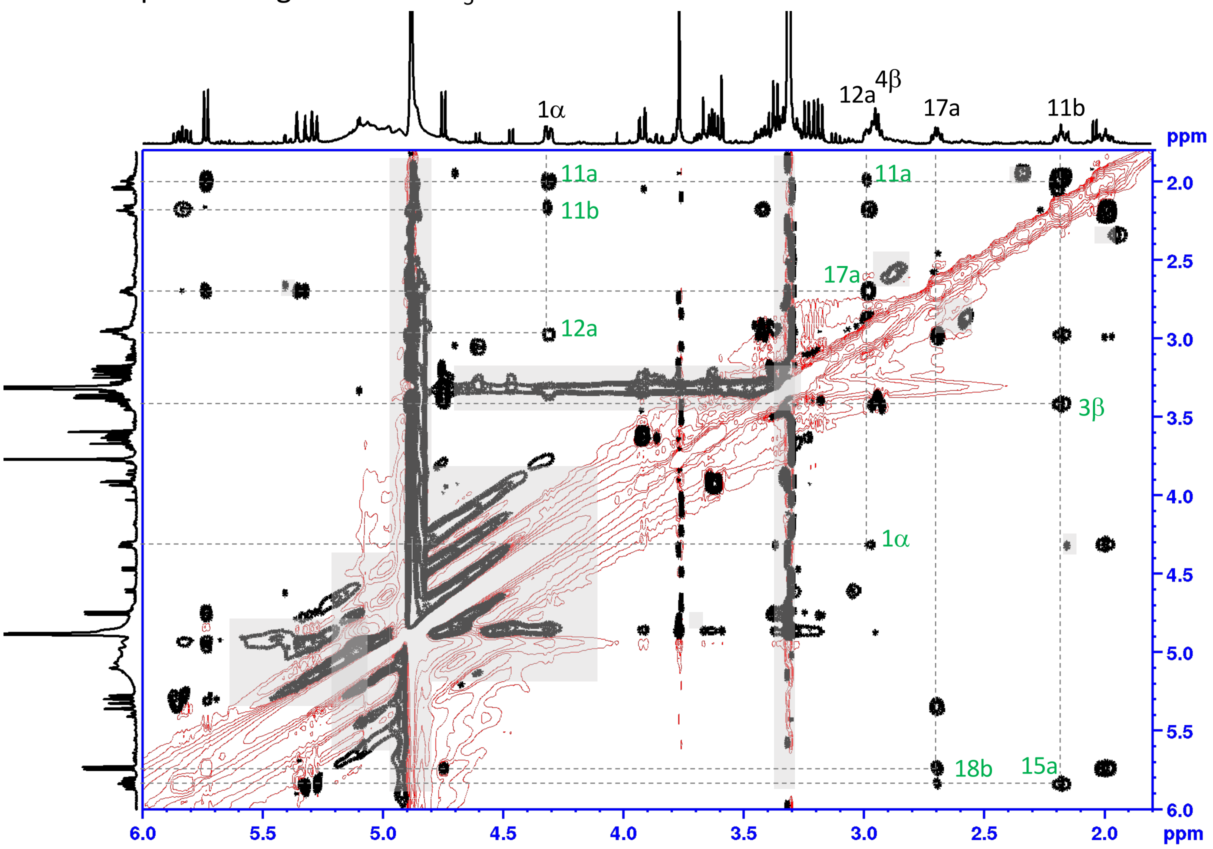


Important ROESY correlations are depicted in green.
Shaded areas mark impurity and solvent

## ROESY correlations of deacetylisoipecoside (aglycon)

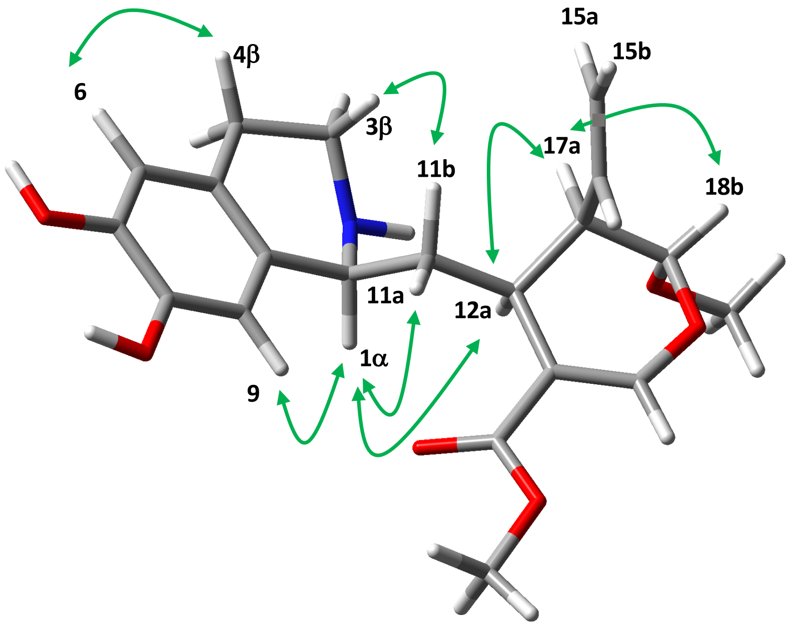


Glucose was replaced with OMe for the modeling.
Optimized using Gaussian 16W (B3LYP/6-31G(d), gas phase). Important ROESY correlations are depicted in green.

**Supplementary Fig. 25. NMR data for demethylalangiside, formed upon spontaneous lactamization of deacetylipecoside.**

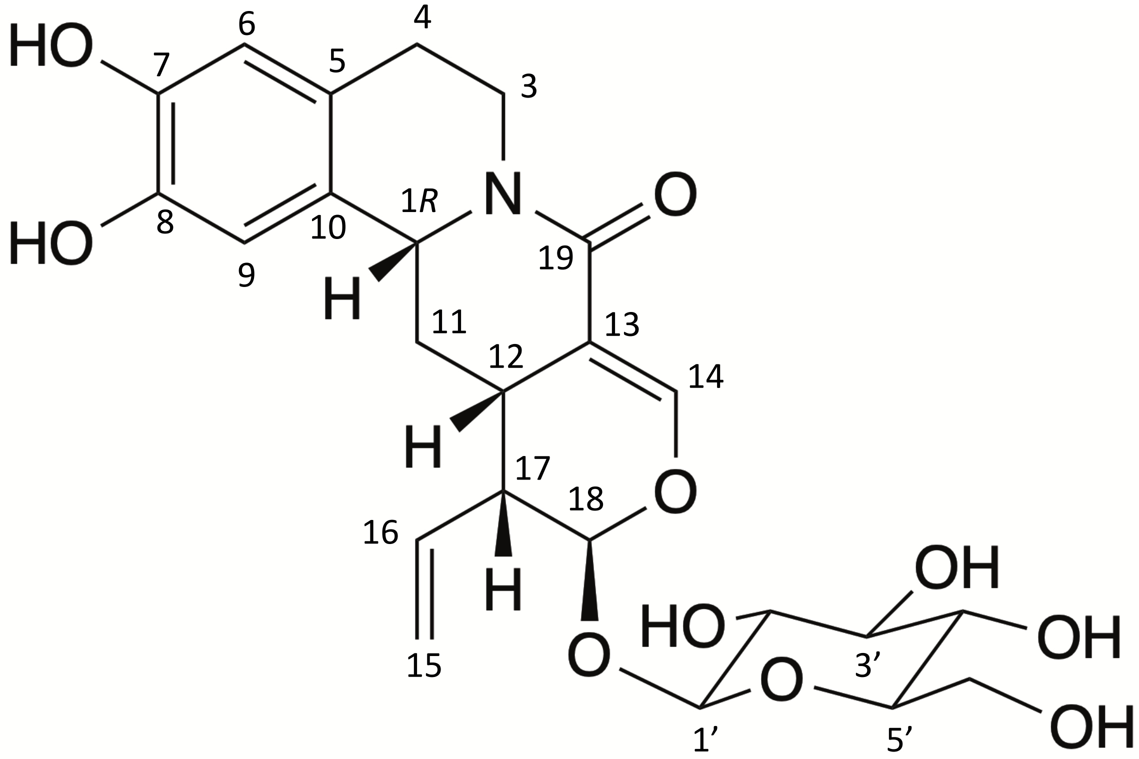


The chemical shifts agreed with the published data [9].
The numbering system is based on a previously published paper [9].

**NMR spectra for demethylalangiside**

presaturated $^1$H NMR full range in MeOH-$d_3$

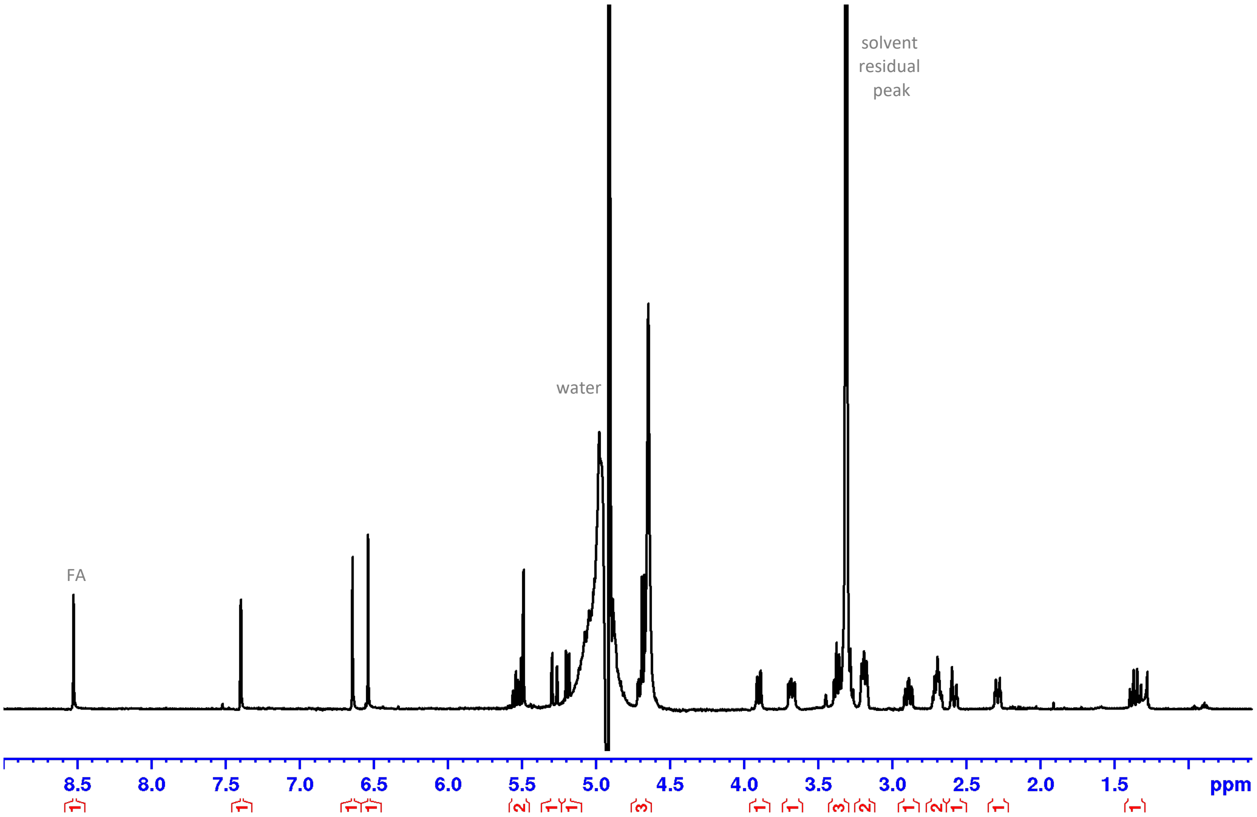


DEPTQ full range in MeOH-$d_3$

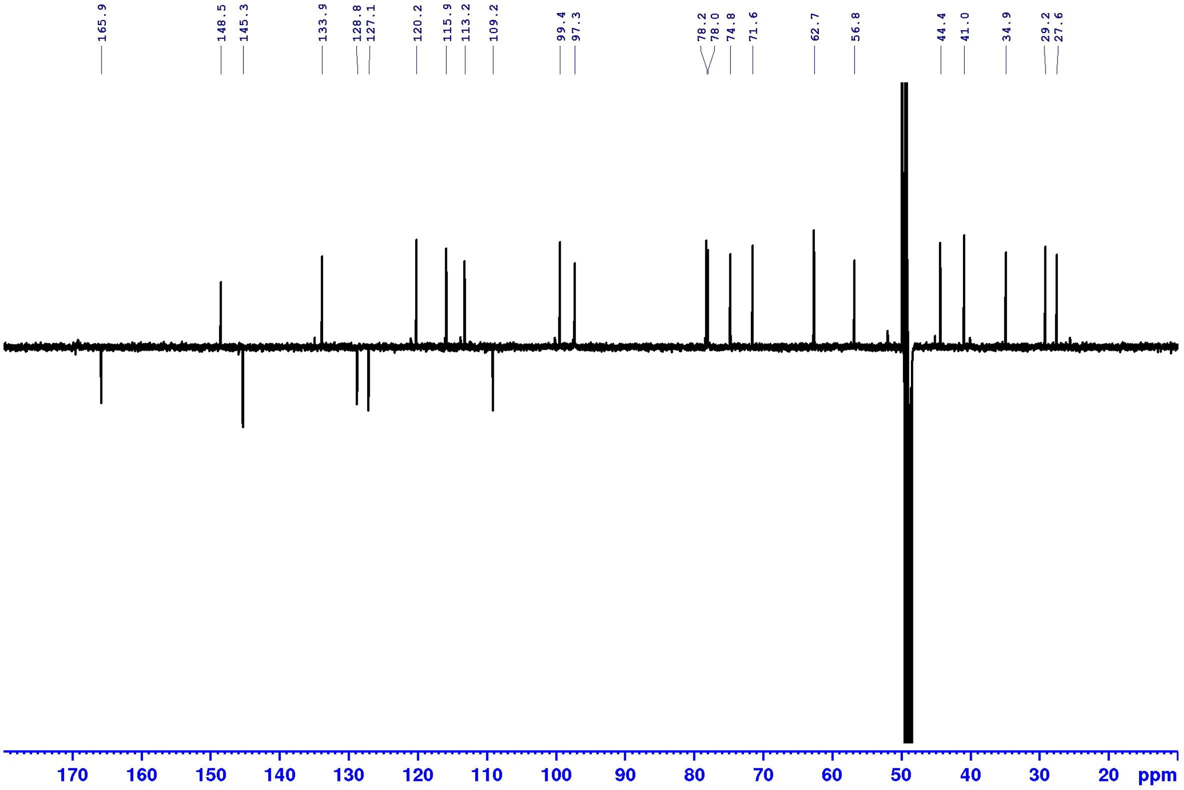

**NMR spectra for demethylalangiside**

Phase sensitive HSQC, full range in MeOH-$d_3$

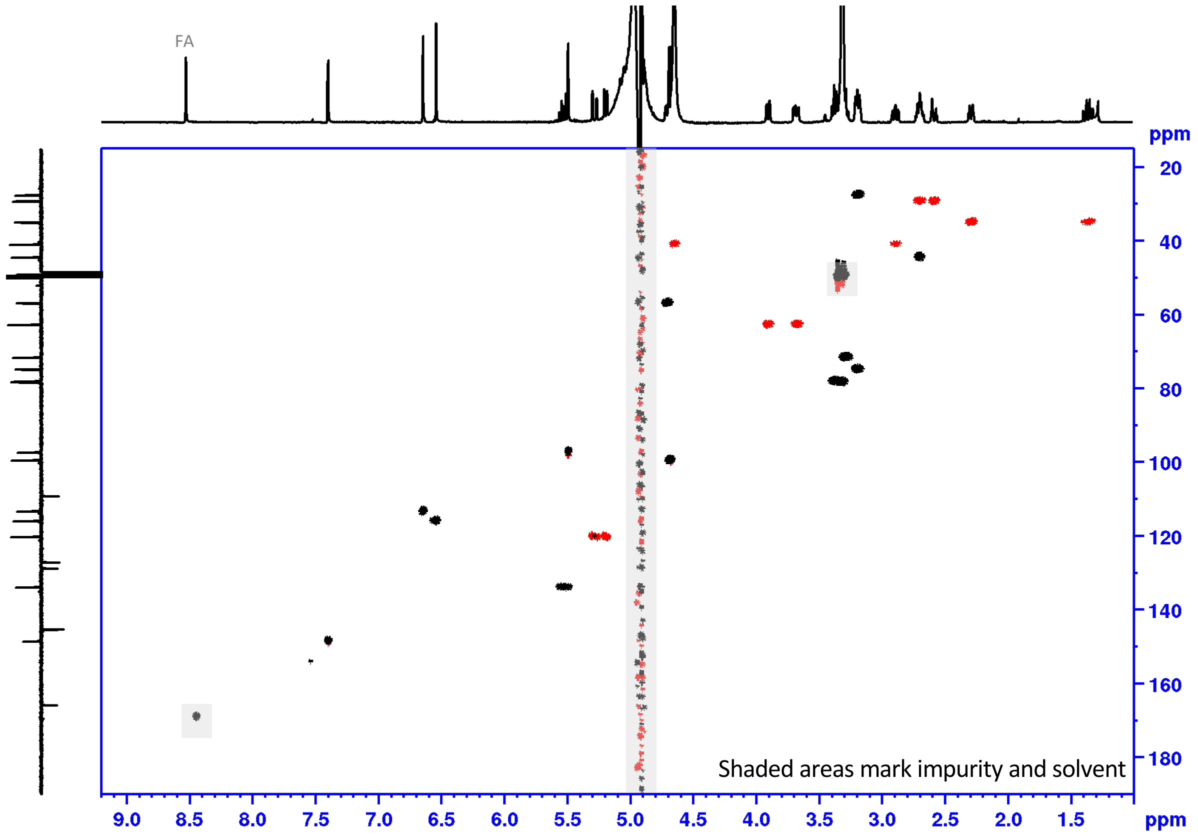


HMBC full range in MeOH-$d_3$

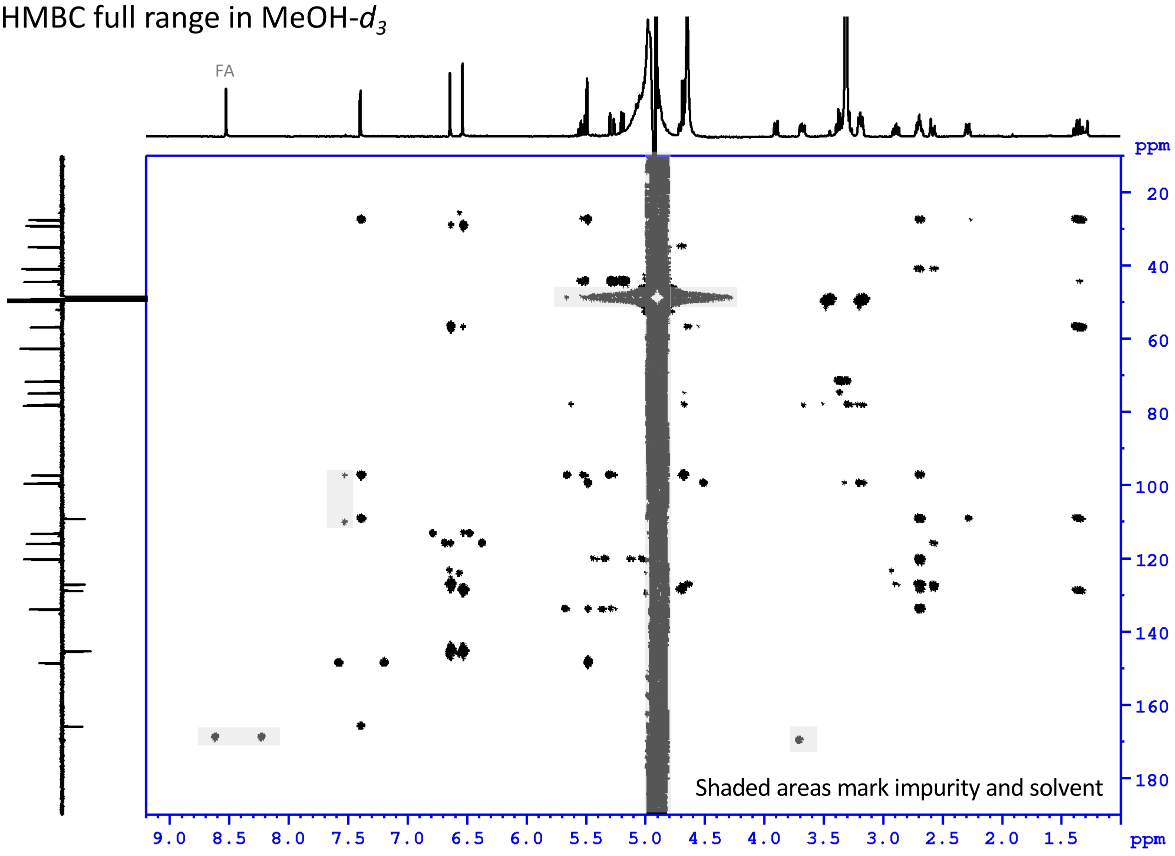

**NMR spectra for demethylalangiside**

COSY, full range in MeOH-$d_3$

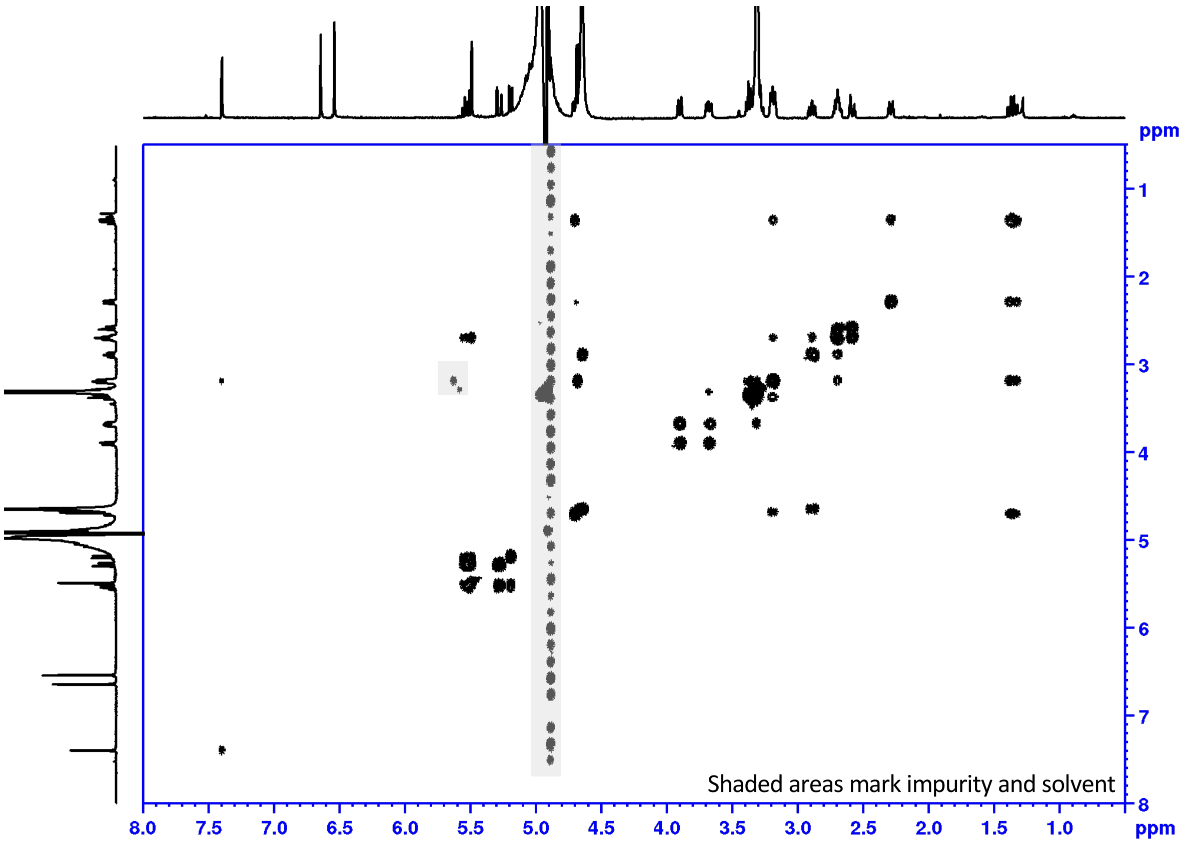


ROESY full range in MeOH-$d_3$

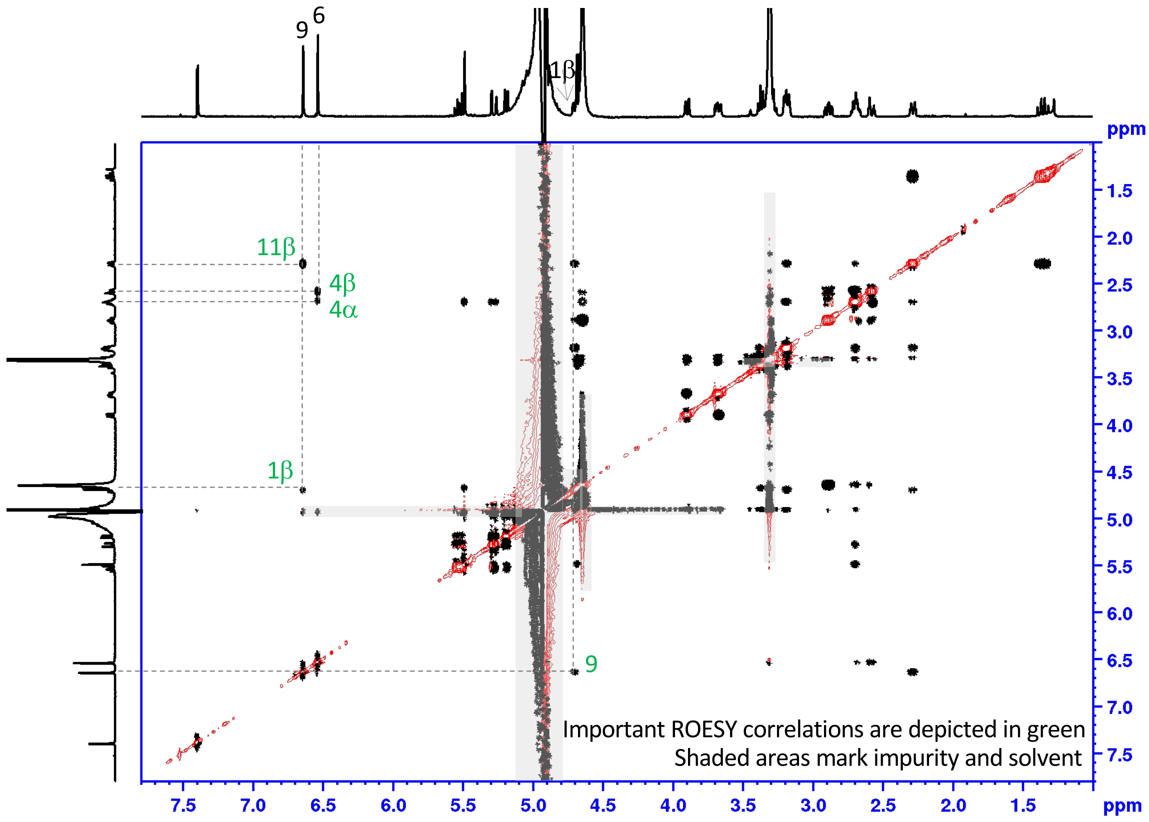

## NMR spectra for demethylalangiside

ROESY aliphatic range in MeOH-$d_3$

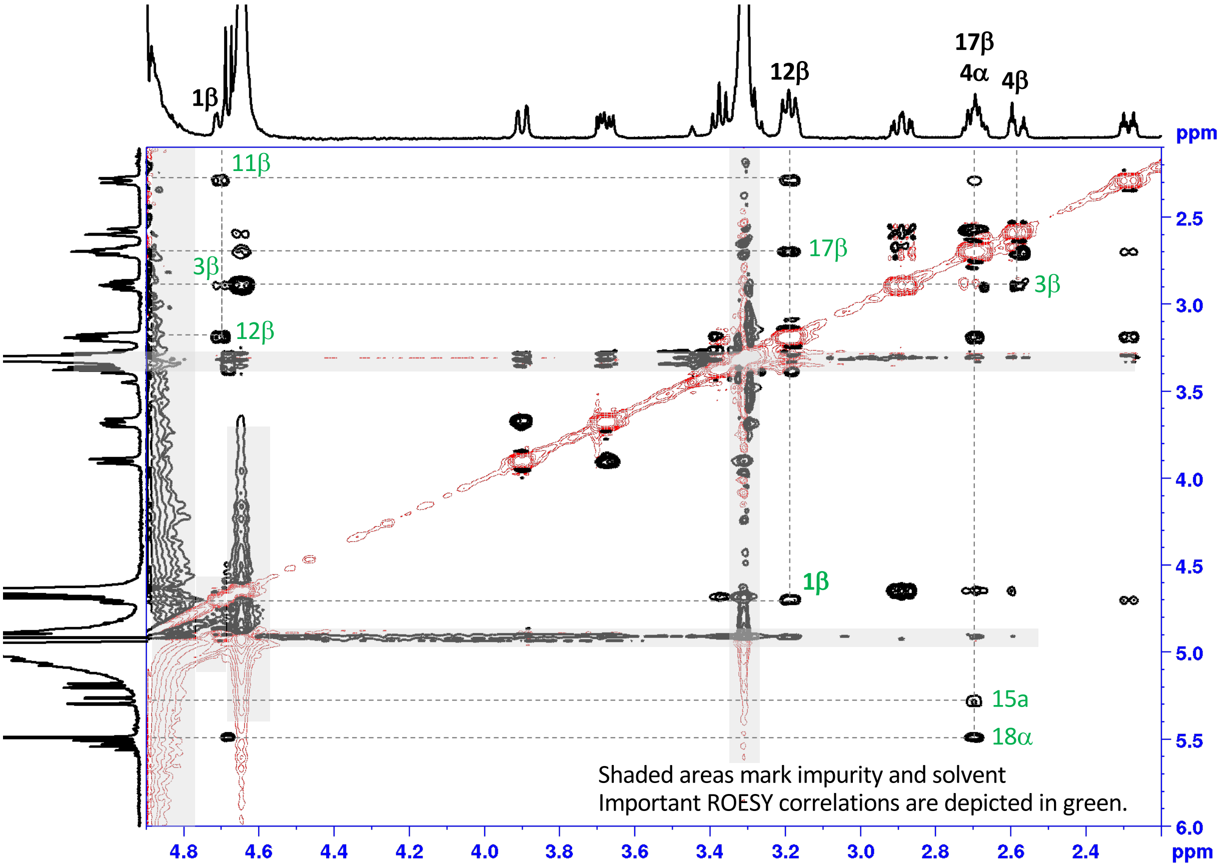


## ROESY correlations of demethylalangiside (aglycon)

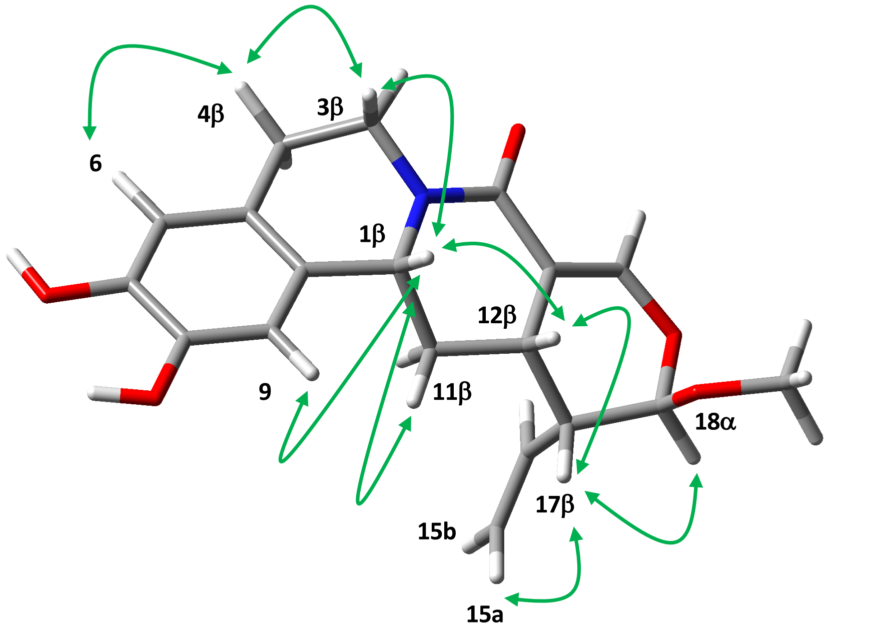


Glucose was replaced with OMe for the modeling.

Optimized using Gaussian 16W (B3LYP/6-31G(d), gas phase). Important ROESY correlations are depicted in green.

**Supplementary Fig. 26. NMR data for synthetic protoemetine.**

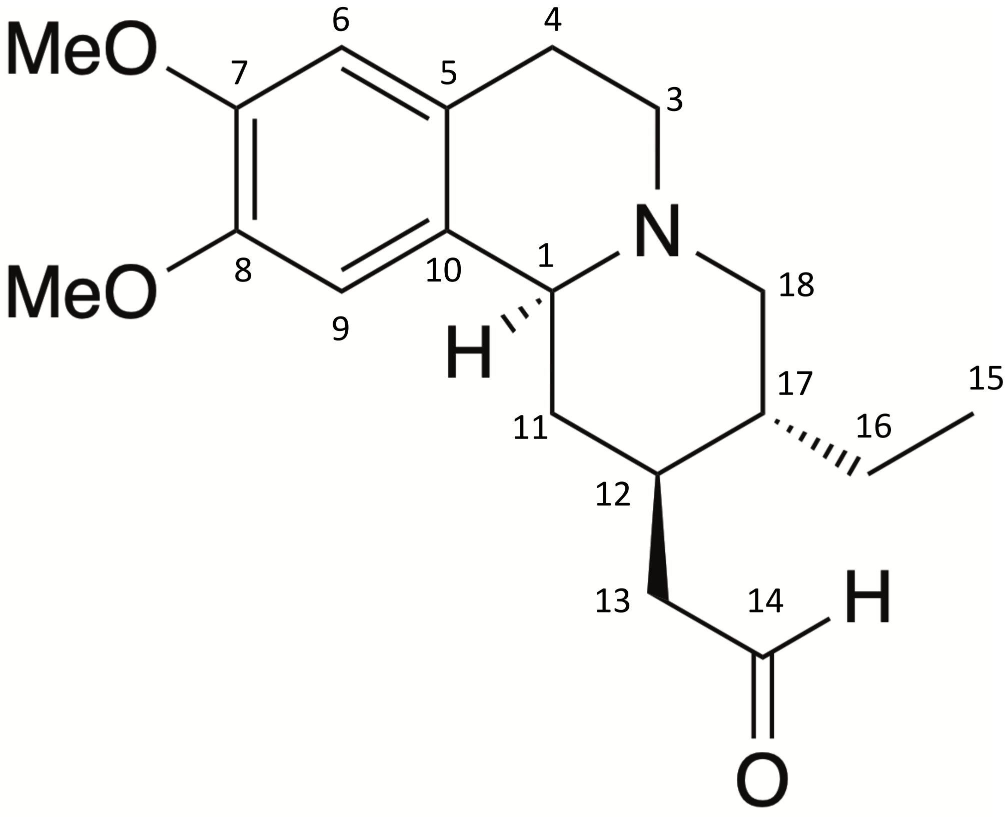


The chemical shifts agreed with the published data [7].
The numbering system is based on a previously published paper [9].

**NMR spectra for protoemetine**

$^1H$ NMR full range in $CDCl_3$

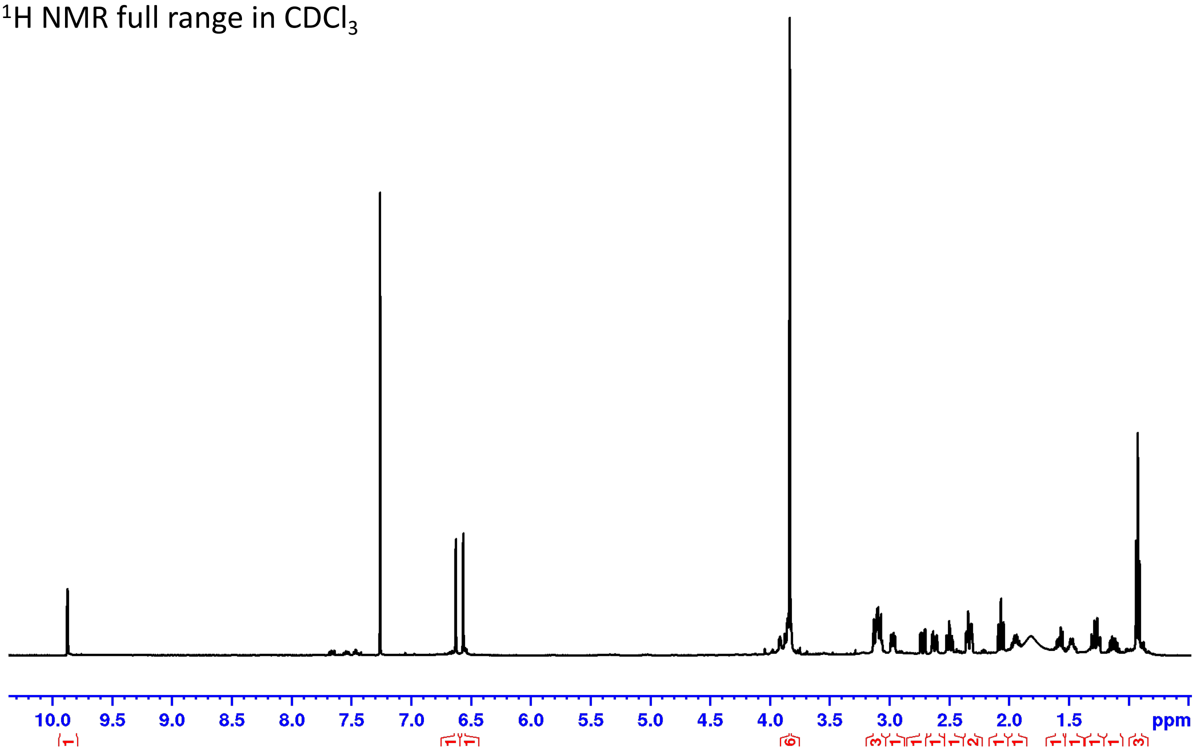


DEPTQ full range in $CDCl_3$

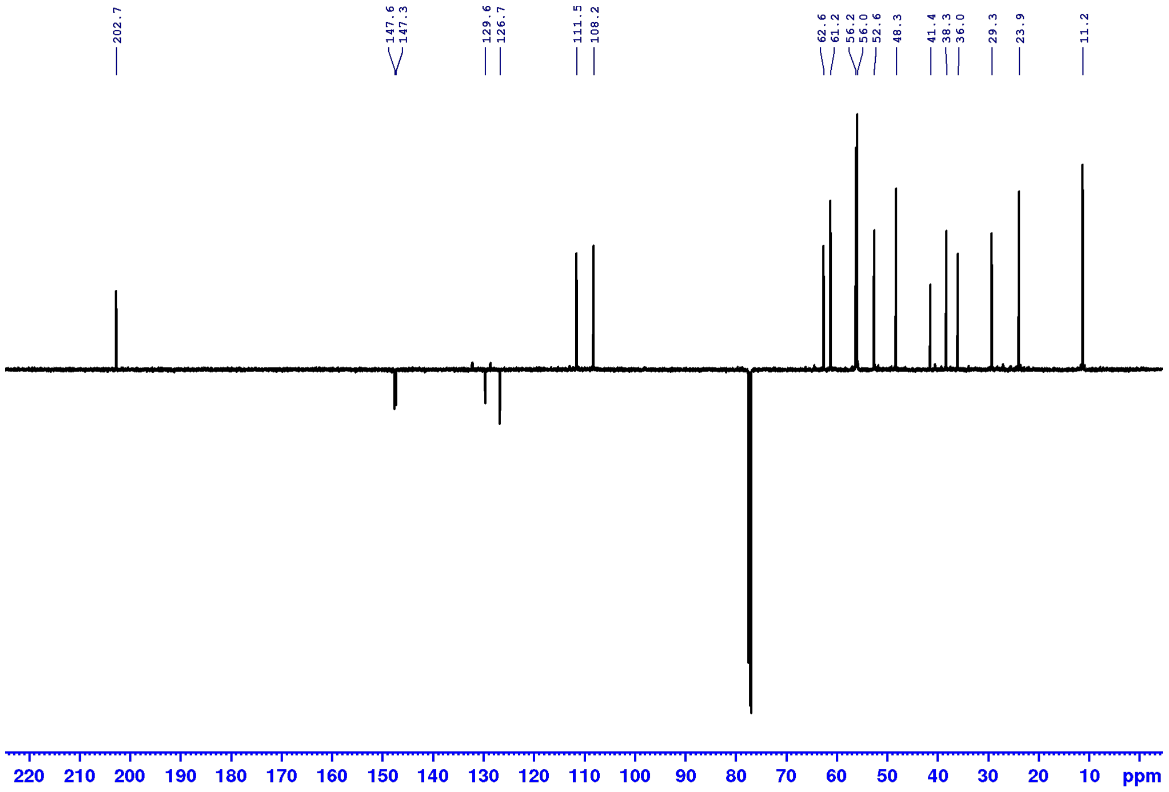

**NMR spectra for protoemetine**

Phase sensitive HSQC, full range in $CDCl_3$

red: $CH_2$, black: CH, $CH_3$

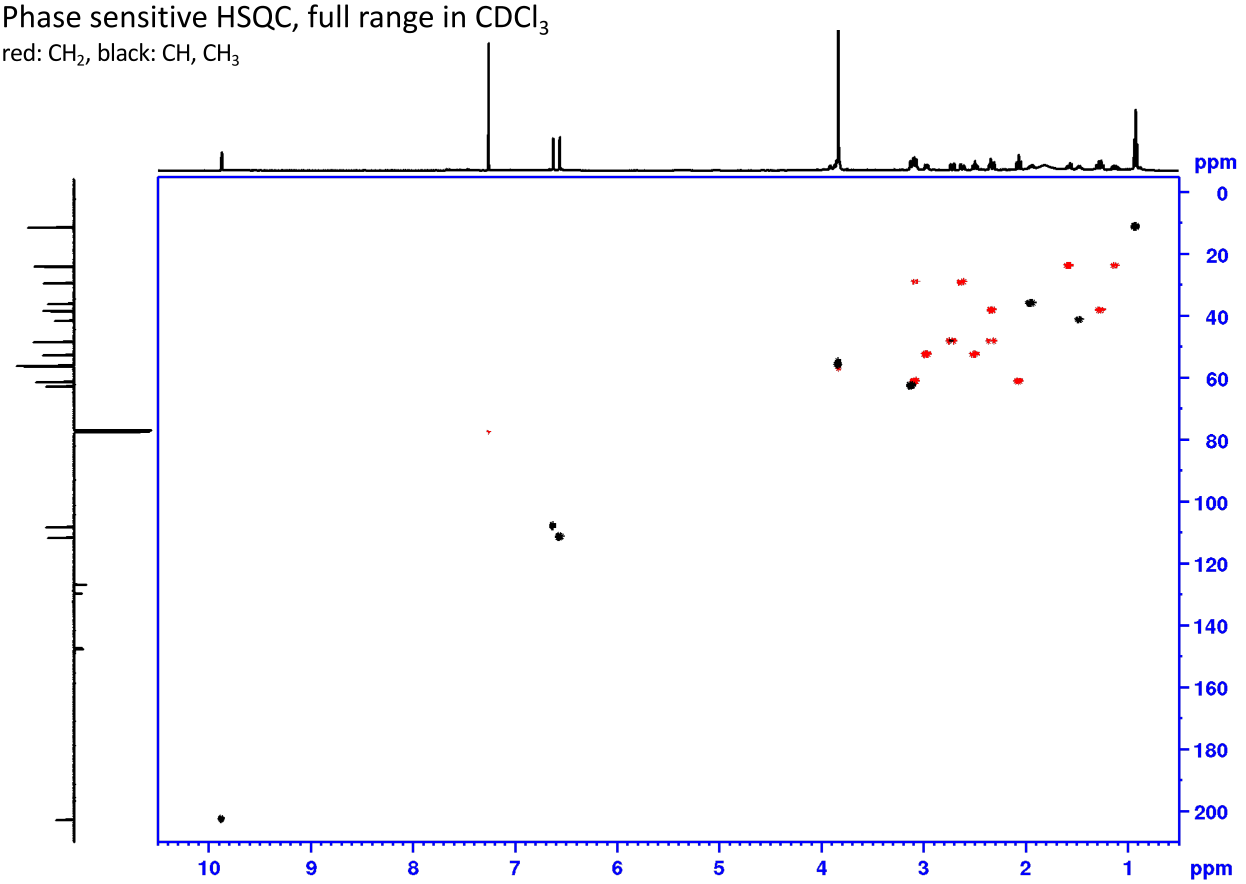


HMBC, full range in $CDCl_3$

Shaded areas mark impurity and solvent

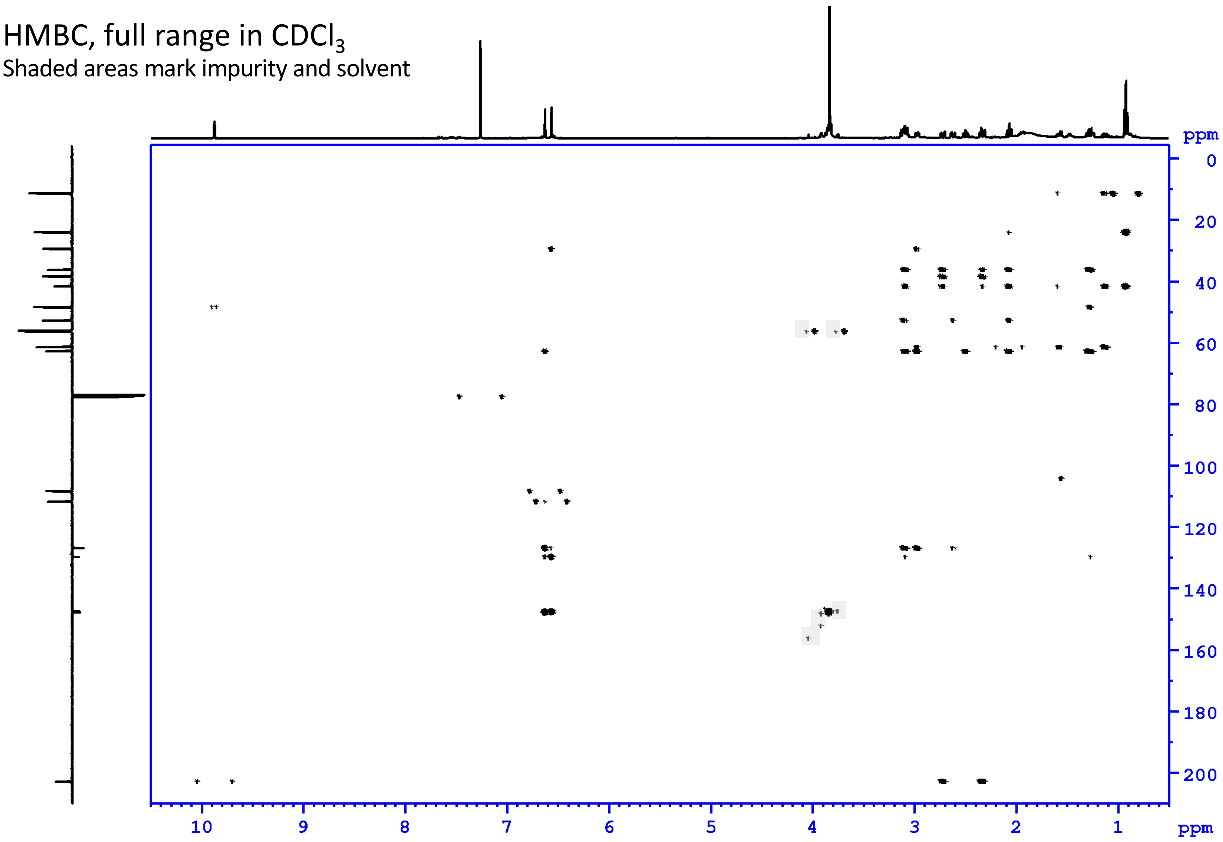

## NMR spectra for protoemetine

ROESY aliphatic range in $CDCl_3$

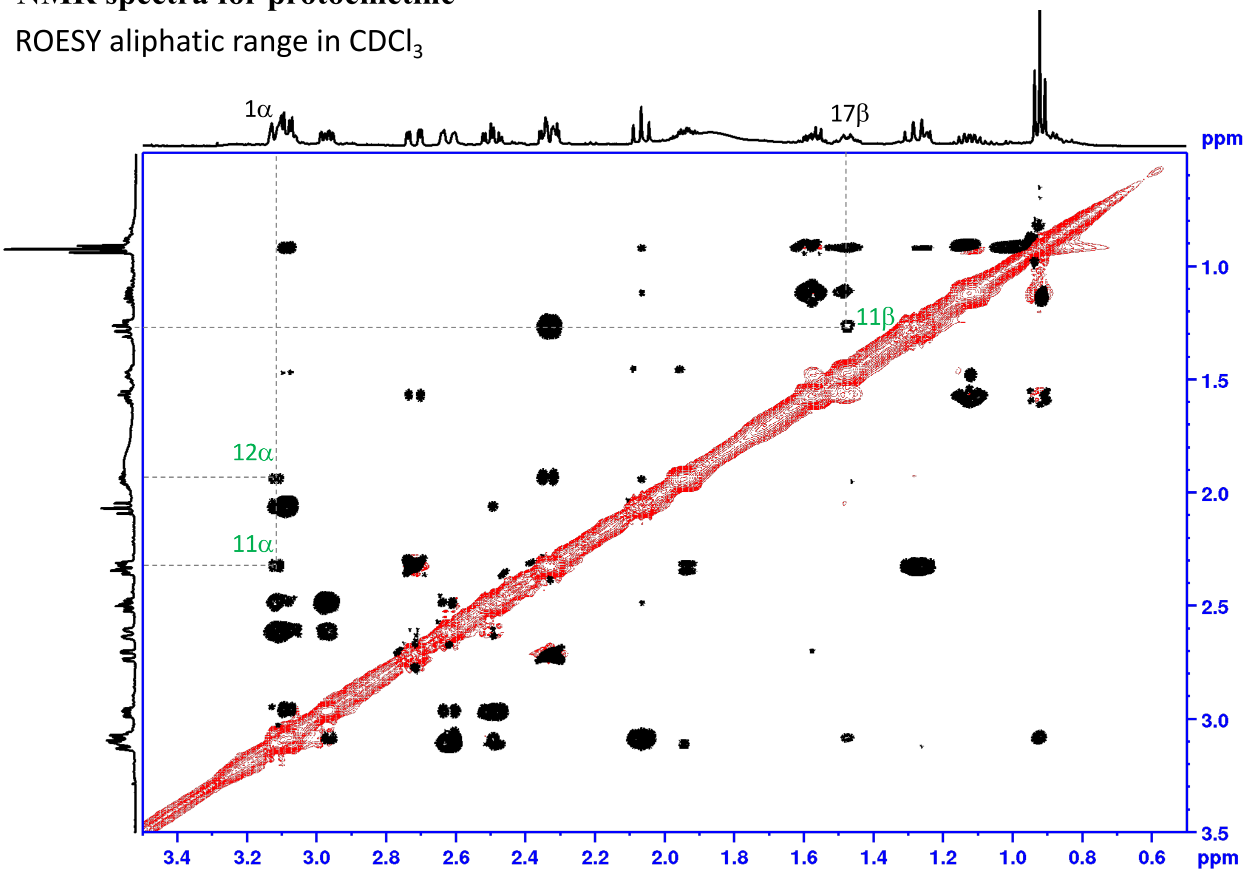


Important ROESY correlations are depicted in green.

## ROESY correlations of protoemetine

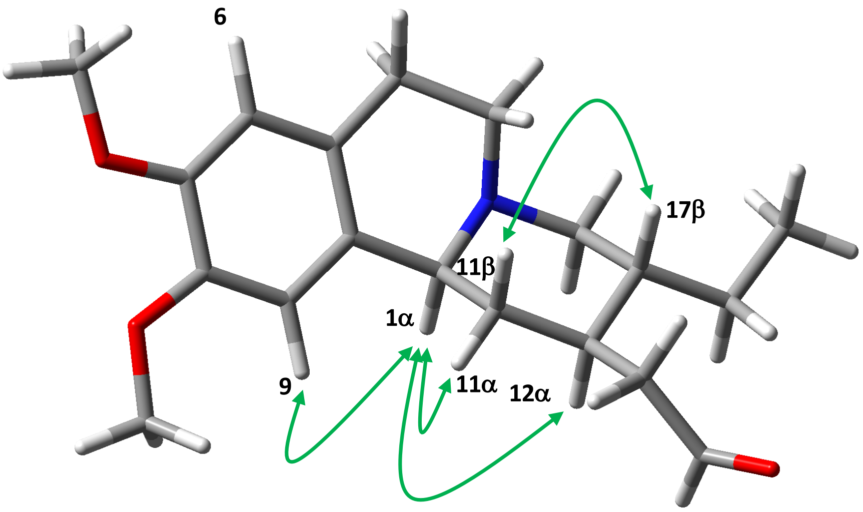


Optimized using Gaussian 16W (PM6, solvent $CDCl_3$).
Important ROESY correlations are depicted in green.

**NMR spectra for protoemetine**

COSY, full range in $CDCl_3$

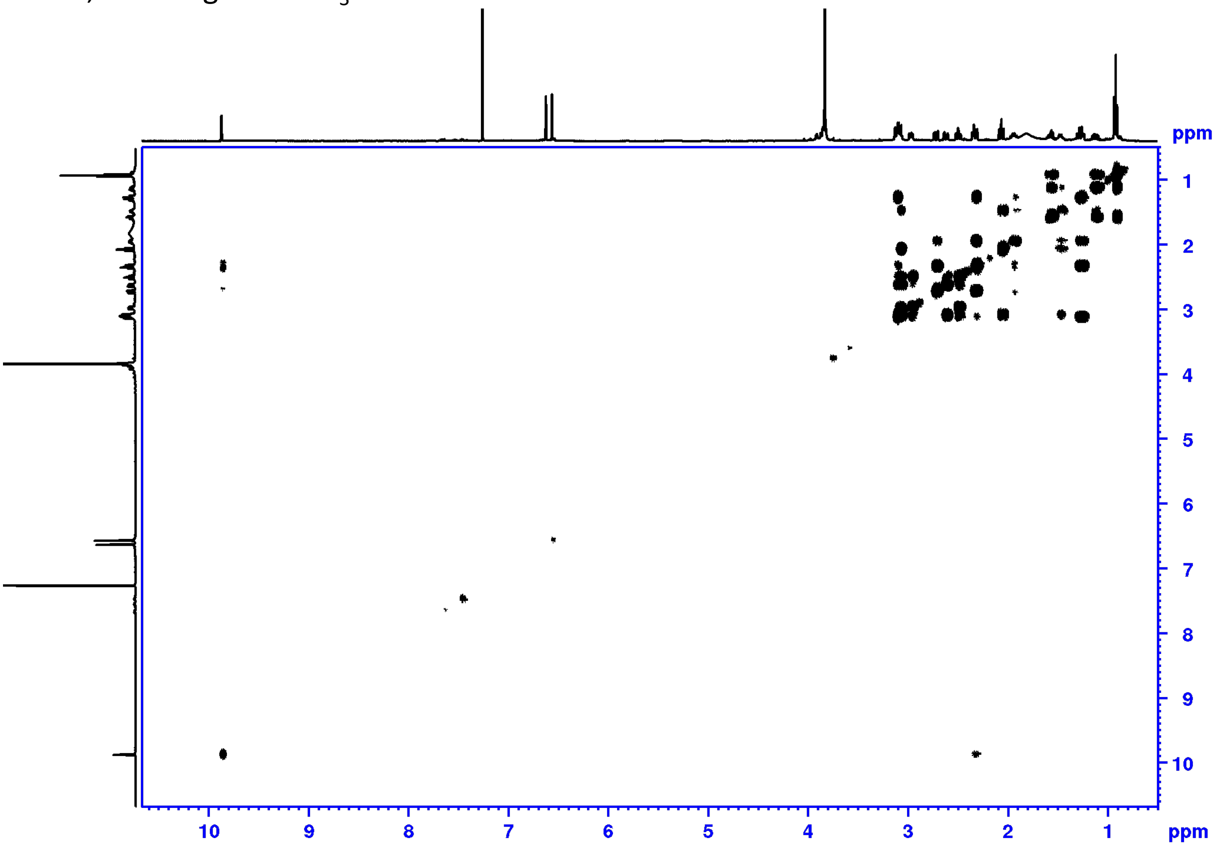


ROESY full range in $CDCl_3$

Shaded areas mark impurity and solvent

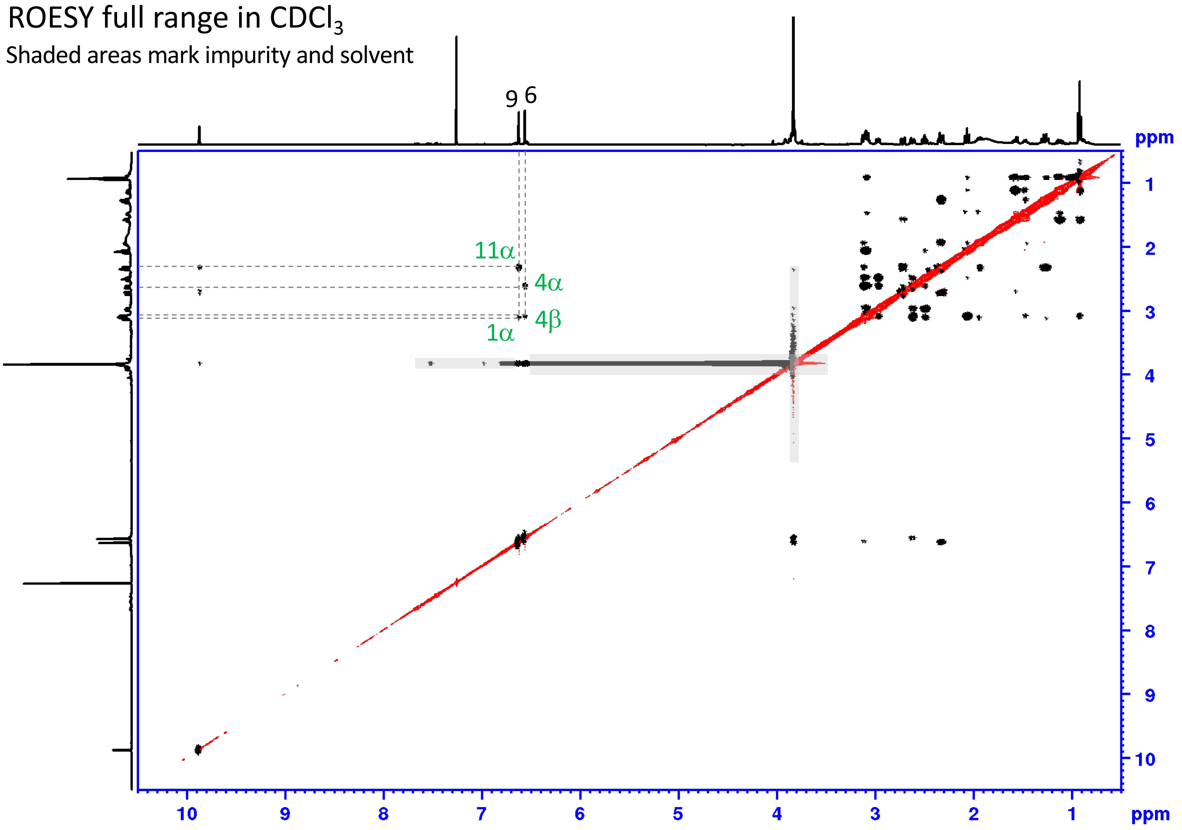


Important ROESY correlations are depicted in green.

# Supplementary Tables

## Supplementary Table 1. Prediction of nuclear localization signal peptide by DeepLOC2.

| Protein | Localizations | Cytoplasm | Nucleus | Extra cellular | Cell membrane | Mitochondrion | Plastid | Endoplasmic reticulum | Lysosome/ Vacuole | Golgi apparatus | Peroxisome | Peripheral | Trans membrane | Lipid anchor |
|---|---|---|---|---|---|---|---|---|---|---|---|---|---|---|
| **CiDGD** | **Nucleus** | 0.277 | **0.804** | 0.338 | 0.063 | 0.102 | 0.009 | 0.110 | 0.160 | 0.097 | 0.302 | 0.199 | 0.130 | 0.202 |
| **CiS6DGD** | **Nucleus** | 0.253 | **0.795** | 0.227 | 0.049 | 0.124 | 0.055 | 0.102 | 0.070 | 0.074 | 0.595 | 0.206 | 0.149 | 0.209 |
| **AsDGD1** | **Nucleus** | 0.308 | **0.730** | 0.277 | 0.191 | 0.092 | 0.083 | 0.148 | 0.229 | 0.092 | 0.245 | 0.230 | 0.119 | 0.176 |
| **AsDGD2** | **Nucleus** | 0.333 | **0.779** | 0.248 | 0.192 | 0.075 | 0.061 | 0.112 | 0.236 | 0.081 | 0.171 | 0.209 | 0.132 | 0.169 |
| **AsS6DGD** | **Cytoplasm** | **0.764** | 0.314 | 0.195 | 0.213 | 0.048 | 0.193 | 0.284 | 0.325 | 0.102 | 0.449 | 0.251 | 0.087 | 0.180 |

**Supplementary Table 2.** NMR chemical shifts for deacetylisoipecoside (500 MHz NMR in MeOH-$d_3$ + 0.1% formic acid). * Overlapped signals *J* unresolved. About position 11a to 18b, please see the ROESY correlation figure.

| pos. | $\delta_H$ | mult. | $J_{HH}$ | $\delta_C$ |
|---|---|---|---|---|
| 1α | 4.31 | *dd* | 11.1/3.2 | 54.4 |
| 3α | 3.35 | *m** | - | 39.9 |
| 3β | 3.41 | *m** | - | 39.9 |
| 4a | 2.95 | *m* | - | 25.5 |
| 4b | 2.95 | *m* | - | 25.5 |
| 5 | - | - | - | 123.2 |
| 6 | 6.58 | *s* | - | 116.1 |
| 7 | - | - | - | 146.9 |
| 8 | - | - | - | 146.0 |
| 9 | 6.56 | *s* | - | 114.1 |
| 10 | - | - | - | 124.2 |
| 11a | 1.99 | *ddd* | 14.8/11.7/3.2 | 36.5 |
| 11b | 2.18 | *ddd* | 14.8/11.1/3.5 | 36.5 |
| 12a | 2.98 | *m* | - | 32.1 |
| 13 | - | - | - | 109.3 |
| 14 | 7.74 | *s* | - | 156.2 |
| 15a | 5.34 | *ddd* | 17.4/1.2/1.2 | 119.7 |
| 15b | 5.28 | *ddd* | 10.5/1.2/1.1 | 119.7 |
| 16 | 5.84 | *ddd* | 17.4/10.5/7.7 | 135.6 |
| 17a | 2.69 | *m* | - | 45.3 |
| 18b | 5.74 | *d* | 8.5 | 97.3 |
| 19 | - | - | - | 170.7 |
| 19-OMe | 3.77 | *s* | - | 52.3 |
| 1' | 4.75 | *d* | 7.9 | 100.2 |
| 2' | 3.19 | *dd* | 9.3/7.9 | 74.7 |
| 3' | 3.38 | *dd* | 9.3/9.1 | 78.0 |
| 4' | 3.23 | *dd* | 9.7/9.1 | 71.6 |
| 5' | 3.32 | *m* | - | 78.5 |
| 6a' | 3.92 | *dd* | 11.8/2.1 | 62.8 |
| 6b' | 3.63 | *dd* | 11.8/6.7 | 62.8 |

The numbering system is based on a previously published paper [9].

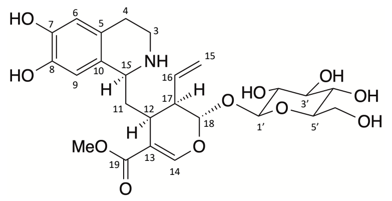

**Supplementary Table 3.** NMR chemical shifts for demethylalangiside (500 MHz NMR in MeOH-*d*3). * Overlapped signals *J* unresolved. About position 15a and 15 b, please see the ROESY correlation figure.

| pos. | $\delta_H$ | mult. | $J_{HH}$ | $\delta_C$ |
|---|---|---|---|---|
| 1β | 4.70 | *brdd* | 11.1/2.9 | 56.8 |
| 3α | 4.65 | *ddd* | 12.4/3.6/3.6 | 41.0 |
| 3β | 2.89 | *ddd* | 12.4/11.3/3.3 | 41.0 |
| 4α | 2.69 | *ddd* | 15.4/11.3/3.6 | 29.2 |
| 4β | 2.58 | *ddd* | 15.4/3.6/3.3 | 29.2 |
| 5 | - | - | - | 127.1 |
| 6 | 6.54 | *s* | - | 115.9 |
| 7 | - | - | - | 145.3 |
| 8 | - | - | - | 145.3 |
| 9 | 6.64 | *s* | - | 113.2 |
| 10 | - | - | - | 128.8 |
| 11α | 1.36 | *ddd* | 13.0/12.8/11.1 | 34.9 |
| 11β | 2.29 | *ddd* | 13.0/3.7/2.9 | 34.9 |
| 12β | 3.19 | *m* | - | 44.4 |
| 13 | - | - | - | 109.2 |
| 14 | 7.40 | *d* | 2.5 | 148.5 |
| 15a | 5.28 | *brdd* | 17.2/2.0 | 120.2 |
| 15b | 5.19 | *brdd* | 10.3/2.0 | 120.2 |
| 16 | 5.52 | *ddd* | 17.2/10.3/9.9 | 133.9 |
| 17β | 2.70 | *brdd* | 9.9/5.8 | 44.4 |
| 18α | 5.49 | *brd* | 1.9 | 97.3 |
| 19 | - | - | - | 165.9 |
| 1' | 4.68 | *d* | 7.8 | 99.4 |
| 2' | 3.19 | *m** | - | 74.8 |
| 3' | 3.37 | *m** | - | 78.0 |
| 4' | 3.29 | *m** | - | 71.6 |
| 5' | 3.31 | *m** | - | 78.2 |
| 6a' | 3.90 | *brd* | 11.4 | 62.7 |
| 6b' | 3.63 | *m* | - | 62.7 |

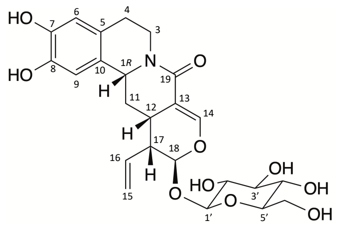

**Supplementary Table 4.** NMR chemical shifts for protoemetine (500 MHz NMR in $CDCl_3$).

| pos. | $\delta_H$ | mult. | $J_{HH}$ | $\delta_C$ |
|---|---|---|---|---|
| 1α | 3.12 | *bd* | 11.8 | 62.6 |
| 3α | 2.97 | *ddd* | 11.2/5.9/1.4 | 52.6 |
| 3β | 2.50 | *ddd* | 11.5/11.2/4.0 | 52.6 |
| 4α | 2.63 | *m* | - | 29.3 |
| 4β | 3.10 | *m* | - | 29.3 |
| 5 | - | - | - | 126.8 |
| 6 | 6.56 | *s* | - | 111.6 |
| 7 | - | - | - | 147.6 |
| 8 | - | - | - | 147.3 |
| 9 | 6.63 | *s* | - | 108.2 |
| 10 | - | - | - | 129.7 |
| 11α | 2.33 | *m* | - | 38.3 |
| 11β | 1.28 | *ddd* | 12.3/11.8/11.8 | 38.3 |
| 12α | 1.95 | *dddd* | 15.4/12.3/8.3/3.8 | 36.0 |
| 13a | 2.72 | *ddd* | 17.1/3.8/1.3 | 48.3 |
| 13b | 2.33 | *ddd* | 17.1/8.3/2.3 | 48.3 |
| 14 | 9.87 | *dd* | 2.3/1.3 | 202.7 |
| 15 | 0.92 | *t* | 7.5 | 11.2 |
| 16a | 1.58 | *m* | - | 23.9 |
| 16b | 1.12 | *m* | - | 23.9 |
| 17 | 1.48 | *m* | - | 41.5 |
| 18α | 2.07 | *dd* | 11.4/11.2 | 61.2 |
| 18β | 3.09 | *dd* | 11.4/4.0 | 61.2 |
| 7-OMe | 3.84 | *s* | - | 56.0 |
| 8-OMe | 3.83 | *s* | - | 56.2 |

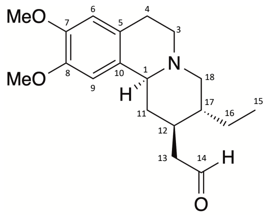

**Supplementary Table 5. List of primers used in this study.**

| Gene name | Sequence | Vector |
|---|---|---|
| *CiDGD* (Nomura et al. 2008: I*peGlu1*) | TTTATGAATTTTGCAGCTCGATGTCTAGTGTTTTGCCTACCCC | Omega |
| | GACAACCACAACAAGCACCGTTAATACTTTCTTAACCTTTTCCTGGG | |
| *CiDOMT1* (Nomura and Kutchan 2010: *IpeOMT1*) | TTTATGAATTTTGCAGCTCGATGGAAACTGTCGAGAGTTCTTCC | |
| | GACAACCACAACAAGCACCGTCAAGGAGAAAGCTCCATGATACA | |
| *CiDOMT2* (Nomura and Kutchan 2010: *IpeOMT2*) | TTTATGAATTTTGCAGCTCGATGGAAACTGTTGAGAGTTCTTC | |
| | GACAACCACAACAAGCACCGTCAAGGAGAAAGCTCGATGATACA | |
| *CiDPOMT* (Nomura and Kutchan 2010: *IpeOMT3*) | TTTATGAATTTTGCAGCTCGATGGAAACTGTTGAGAGTTCTTCC | |
| | GACAACCACAACAAGCACCGTTAGTTATAAAAATTAACCTCAATGAG | |
| *CiS6DGD* | TTTATGAATTTTGCAGCTCGATGGCTACTGTTTTGGCTACCC | |
| | GACAACCACAACAAGCACCGTTACCTTCTTAATCTTTTCCTGGAGG | |
| *CiDE* | TTTATGAATTTTGCAGCTCGATGGTGGACACCACTGCAAA | |
| | GACAACCACAACAAGCACCGTTAATTGGAGTTTAGTGTCCGAGC | |
| *CiDR1* | TTTATGAATTTTGCAGCTCGATGGCACAATCACCAGAGACG | |
| | GACAACCACAACAAGCACCGTTAAGGTGATTTCAATGAGCTCATGT | |
| *CiDR2* | TTTATGAATTTTGCAGCTCGATGGCAAAATCACCGGAGACG | |
| | GACAACCACAACAAGCACCGTTAAGATGACTTCAATGAGCTCATGTC | |
| *CiIpS* | TTTATGAATTTTGCAGCTCGATGGAGATATCATCAAAGGAGTTGATT | |
| | GACAACCACAACAAGCACCGTCAGAAATAGGCAGTTGTCTCAAG | |
| *AsDOMT1* | TTTATGAATTTTGCAGCTCGATGAGTTTAATCAAAGGACCATTAAAG | |
| | GACAACCACAACAAGCACCGTCAGTAGACTCGCCTGCAGAT | |
| *AsDOMT2* | TTTATGAATTTTGCAGCTCGATGAGTTTAATGAAAGGACC | |
| | GACAACCACAACAAGCACCGTCAGGAGACTCGTCTGCAGAC | |
| *AsDOMT3* | TTTATGAATTTTGCAGCTCGATGGATAAGAAGCCAAGCAAAGGGT | |
| | GACAACCACAACAAGCACCGTCAGGAGACACGCATGCAGATA | |
| *AsDOMT4* | TTTATGAATTTTGCAGCTCGATGGACGTGACTGTCAGCA | |
| | GACAACCACAACAAGCACCGTCAGGAGAGTCGCCTGCAGAT | |
| *AsDOMT5* | TTTATGAATTTTGCAGCTCGATGGACGTGACTGTTAGCAAGG | |
| | GACAACCACAACAAGCACCGTCAGGAGAGTCGCCTGCAGAT | |
| *AsDOMT6* | TTTATGAATTTTGCAGCTCGATGAGGCCTAGCAAAGGATTGT | |
| | GACAACCACAACAAGCACCGTCAGTAGACTCGCCTGCAGA | |
| *AsDOMT7* | TTTATGAATTTTGCAGCTCGATGAGGCCTAGCAAAGGATTGT | |
| | GACAACCACAACAAGCACCGTCAGTAGACTCGCCTGCAGA | |
| *AsS6DGD* | TTTATGAATTTTGCAGCTCGATGAGCATGGACTGCGTTTAC | |
| | GACAACCACAACAAGCACCGTCAATTGGGAAGCCCCTCTT | |
| *AsDGD1* | TTTATGAATTTTGCAGCTCGATGGCGAAGACCCCATCG | |
| | GACAACCACAACAAGCACCGTTAAATACCTACTCTAGAGGCTCTTCT | |
| *AsDGD2* | TTTATGAATTTTGCAGCTCGATGGCGAAGACCCCATCG | |
| | GACAACCACAACAAGCACCGTTAAACACCCACTCTAGAGGCTC | |
| *AsDR1* | TTTATGAATTTTGCAGCTCGATGGCGAAAGCACCGGAGA | |
| | GACAACCACAACAAGCACCGCTAGTAGGGGTTTTTCAAGGTGCT | |

| | | |
|---|---|---|
| *AsDR2* | TTTATGAATTTTGCAGCTCGATGGCGAAGTCGCCAGAGAC | |
| | GACAACCACAACAAGCACCGTTAGGGGGTCTTCAAGGTGT | |
| *AsDPOMT1* | TTTATGAATTTTGCAGCTCGATGAGTTGGGCCGGCGAGAT | |
| | GACAACCACAACAAGCACCGTCAATTCTTCTTCAGATATTCCATAAT | |
| *AsDPOMT2* | TTTATGAATTTTGCAGCTCGATGAGTTGGGCCGGCGAGATAA | |
| | GACAACCACAACAAGCACCGTCAATTCTTCTTCAAATATTCCA | |
| *CiDOMT1* | AAGTTCTGTTTCAGGGCCCGGAAACTGTCGAGAGTTCTTCCTC | pOPINF |
| | ATGGTCTAGAAAGCTTTATCAAGGAGAAAGCACCATGA | |
| *CiDE* | AAGTTCTGTTTCAGGGCCCGGTGGACACCACTGCAAAACA | |
| | ATGGTCTAGAAAGCTTTATTAATTGGAGTTTAGTGTCCGAGC | |
| *AsDOMT3* | AAGTTCTGTTTCAGGGCCCGGATAAGAAGCCAAGCAAAGGGT | |
| | ATGGTCTAGAAAGCTTTATCAGGAGACACGCATGCAG | |
| *CiDGD* | AAGTTCTGTTTCAGGGCCCGTCTAGTGTTTTGCCTACCCCTG | |
| | ATGGTCTAGAAAGCTTTATTAATACTTTCTTAACCTTTTCCTGGG | |
| *CiS6DGD* | AAGTTCTGTTTCAGGGCCCGGCTACTGTTTTGGCTACCCCT | |
| | ATGGTCTAGAAAGCTTTATTACCTTCTTAATCTTTTCCTGGAGG | |
| *AsDGD2* | AAGTTCTGTTTCAGGGCCCGGCGAAGACCCCATCGCTC | |
| | ATGGTCTAGAAAGCTTTATTAAACACCCACTCTAGAGGCTC | |
| *AsS6DGD* | AAGTTCTGTTTCAGGGCCCGAGCATGGACTGCGTTTACAGA | |
| | ATGGTCTAGAAAGCTTTAATTGGGAAGCCCCTCTTTGT | |
| *AsDGD1* | CCTGACCTAGGTCTCCAATGGCGAAGACCCCATCG | alpha |
| | AAGCCTGGTCTCTGCTTAATACCTACTCTAGAGGCTCTTCTCT | |
| | CCTGACCTAGGTCTCTAAGCTGGCGAAGACCCCATCGCTC | |
| | TAGCCTGGTCTCTAAGCTTAAATACCTACTCTAGAGGCTCTTCTCT | |
| *AsDGD2* | CCTGACCTAGGTCTCCAATGGCGAAGACCCCATCG | |
| | AAGCCTGGTCTCTGCTTAACACCCACTCTAGAGGCTC | |
| | CCTGACCTAGGTCTCTAAGCTGGCGAAGACCCCATCGCTC | |
| | TAGCCTGGTCTCTAAGCTTAAACACCCACTCTAGAGGCTC | |
| *CiDGD* | CCTGACCTAGGTCTCCAATGTCTAGTGTTTTGCCTACCCC | |
| | AAGCCTGGTCTCTGCTTATACTTTCTTAACCTTTTCCTGGGG | |
| | CCTGACCTAGGTCTCTAAGCTGTCTAGTGTTTTGCCTACCCCTG | |
| | TAGCCTGGTCTCTAAGCTTAATACTTTCTTAACCTTTTCCTGGG | |
| *CiS6DGD* | CCTGACCTAGGTCTCCAATGGCTACTGTTTTGGCTACCC | |
| | AAGCCTGGTCTCTGCTTCCTTCTTAATCTTTTCCTGGAGG | |
| | CCTGACCTAGGTCTCTAAGCTGGCTACTGTTTTGGCTACCCCT | |
| | TAGCCTGGTCTCTAAGCTTACCTTCTTAATCTTTTCCTGGAGG | |
| *AsS6DGD* | CCTGACCTAGGTCTCTAAGCTGAGCATGGACTGCGTTTACAGA | |
| | TAGCCTGGTCTCTAAGCTTATCAATTGGGAAGCCCCTCTT | |
| | CCTGACCTAGGTCTCCAATGAGCATGGACTGCGTTTAC | |
| | AAGCCTGGTCTCTGCTTATTGGGAAGCCCCTCTTTGT | |
| | TTCGGTCATCAGGGACCTCTC | |
| | GAGAGGTCCCTGATGACCGAA | |
| *CiDE* | CCTGACCTAGGTCTCTAAGCTGGTGGACACCACTGCAAAACA | |
| | TAGCCTGGTCTCTAAGCTTAATTGGAGTTTAGTGTCCGAGCA | |

| | | |
|---|---|---|
| | CCTGACCTAGGTCTCCAATGGTGGACACCACTGCAAA | |
| | AAGCCTGGTCTCTGCTTATTGGAGTTTAGTGTCCGAGCA | |
| *CiIpS* | CCTGACCTAGGTCTCTAAGCTGGAGATATCATCAAAGGAGTTGA | |
| | TAGCCTGGTCTCTAAGCTTATCAGAAATAGGCAGTTGTCTCA | |
| | CCTGACCTAGGTCTCCAATGGAGATATCATCAAAGGAGT | |
| | AAGCCTGGTCTCTGCTTGAAATAGGCAGTTGTCTCAAGTG | |
| | GAATCGGAGATCACTACCAAGA | |
| | TCTTGGTAGTGATCTCCGATTC | |
| *CiDR1* | CCTGACCTAGGTCTCCAATGGCACAATCACCAGAGAC | |
| | AAGCCTGGTCTCTGCTTAGGTGATTTCAATGAGCTCATGT | |
| | CCTGACCTAGGTCTCTAAGCTGGCACAATCACCAGAGACGGA | |
| | TAGCCTGGTCTCTAAGCTTAAGGTGATTTCAATGAGCTCATGT | |
| *EYFP/MCERULEAN3* | CCTGACCTAGGTCTCCAATGGTGAGCAAGGGCGAG | |
| | AAGCCTGGTCTCTGCTTCTTGTACAGCTCGTCCATGC | |
| | CCTGACCTAGGTCTCTAAGCTGGTGAGCAAGGGCGAGGAG | |
| | TAGCCTGGTCTCTAAGCTTACTTGTACAGCTCGTCCATGC | |

**Supplementary Table 6. Accession numbers of genes described in this study.**

| Gene name | Accession number |
|---|---|
| *AsDGD1* | PQ363556 |
| *AsDGD2* | PQ363557 |
| *AsDOMT1* | PQ363558 |
| *AsDOMT2* | PQ363559 |
| *AsDOMT3* | PQ363560 |
| *AsDOMT4* | PQ363561 |
| *AsDOMT5* | PQ363562 |
| *AsDOMT6* | PQ363563 |
| *AsDOMT7* | PQ363564 |
| *AsDPOMT1* | PQ363565 |
| *AsDPOMT2* | PQ363566 |
| *AsDR1* | PQ363567 |
| *AsDR2* | PQ363568 |
| *AsS6DGD* | PQ363569 |
| *CiDE* | PQ363570 |
| *CiDGD* | PQ363571 |
| *CiDOMT1* | PQ363572 |
| *CiDOMT2* | PQ363573 |
| *CiDPOMT* | PQ363574 |
| *CiDR1* | PQ363575 |
| *CiDR2* | PQ363576 |
| *CiIpS* | PQ363577 |
| *CiS6DGD* | PQ363578 |

## Supplementary references


1. Xie, C., Luo, J., Zhang, Y., Zhu, L. & Hong, R. A Chiral Pentenolide-Based Unified Strategy toward Dihydrocorynantheal, Dihydrocorynantheol, Protoemetine, Protoemetinol, and Yohimbane. *Org Lett* **19**, 3592-3595 (2017).
2. Marigo, M., Fielenbach, D., Braunton, A., Kjaersgaard, A. & Jorgensen, K.A. Enantioselective formation of stereogenic carbon-fluorine centers by a simple catalytic method. *Angew Chem Int Ed Engl* **44**, 3703-6 (2005).
3. Hayashi, Y., Gotoh, H., Hayashi, T. & Shoji, M. Diphenylprolinol silyl ethers as efficient organocatalysts for the asymmetric Michael reaction of aldehydes and nitroalkenes. *Angew Chem Int Ed Engl* **44**, 4212-5 (2005).
4. Akwaboah, D.C., Wu, D. & Forsyth, C.J. Stereoselective Synthesis of the C1-C9 and C11-C25 Fragments of Amphidinolides C, C2, C3, and F. *Org Lett* **19**, 1180-1183 (2017).
5. Bodenschatz, K., Stöckl, J., Winterer, M. & Schobert, R. A synthetic approach to 5/5/6-polycyclic tetramate macrolactams of the discodermide type. *Tetrahedron* **104**(2022).
6. Zhong, Y.-L. & Shing, T.K.M. Efficient and Facile Glycol Cleavage Oxidation Using Improved Silica Gel-Supported Sodium Metaperiodate. *J Org Chem* **62**, 2622-2624 (1997).
7. Lin, S., Deiana, L., Tseggai, A. & Córdova, A. Concise Total Synthesis of Dihydrocorynanthenol, Protoemetinol, Protoemetine, 3-epi-Protoemetinol and Emetine. *European J Org Chem* **2012**, 398-408 (2011).
8. Yeon, S.H., Jeon, J.S., Um, K.A., Kim, C.Y. & Ahn, Y.J. Large-scale purification of unstable, water-soluble secologanic acid using centrifugal partition chromatography. *Phytochem Anal* **29**, 487-492 (2018).
9. Beke, G., Szabó, L.F. & Podányi, B. Regio- and Stereoselectivity in the Coupling Reaction of Secologanin with Dopamine Derivatives. *J Nat Prod* **64**, 332-340 (2001).
10. De-Eknamkul, W., Suttipanta, N. & Kutchan, T.M. Purification and characterization of deacetylipecoside synthase from *Alangium lamarckii* Thw. *Phytochemistry* **55**, 177-181 (2000).
11. DeEknamkul, W., Ounaroon, A., Tanahashi, T., Kutchan, T.M. & Zenk, M.H. Enzymatic condensation of dopamine and secologanin by cell-free extracts of Alangium lamarckii. *Phytochemistry* **45**, 477-484 (1997).
12. Nomura, T., Quesada, A.L. & Kutchan, T.M. The new beta-D-glucosidase in terpenoid-isoquinoline alkaloid biosynthesis in *Psychotria ipecacuanha*. *J Biol Chem* **283**, 34650-9 (2008).
13. Nomura, T. & Kutchan, T.M. Three New O-Methyltransferases Are Sufficient for All O-Methylation Reactions of Ipecac Alkaloid Biosynthesis in Root Culture of Psychotria ipecacuanha. *J Biol Chem* **285**, 7722-7738 (2010).
