# Supplementary material for: Ipecac alkaloid biosynthesis in two evolutionarily distant plants
Source: Nat Chem Biol. 2025 Jun 3;21(11):1794–805. doi: 10.1038/s41589-025-01926-z (PMC12568652; doi:10.1038/s41589-025-01926-z)
Supplement: Supplementary file 2 — Reporting Summary [file 41589_2025_1926_MOESM2_ESM.pdf]

nature portfolio

Corresponding author(s): Maite Colinas
Sarah E. O'Connor

Last updated by author(s): Mar 19, 2025

Reporting Summary

Nature Portfolio wishes to improve the reproducibility of the work that we publish. This form provides structure for consistency and transparency in reporting. For further information on Nature Portfolio policies, see our Editorial Policies and the Editorial Policy Checklist.

Statistics

For all statistical analyses, confirm that the following items are present in the figure legend, table legend, main text, or Methods section.

n/a	Confirmed	
☐	☒	The exact sample size (*n*) for each experimental group/condition, given as a discrete number and unit of measurement
☐	☒	A statement on whether measurements were taken from distinct samples or whether the same sample was measured repeatedly
☒	☐	The statistical test(s) used AND whether they are one- or two-sided<br>*Only common tests should be described solely by name; describe more complex techniques in the Methods section.*
☒	☐	A description of all covariates tested
☒	☐	A description of any assumptions or corrections, such as tests of normality and adjustment for multiple comparisons
☐	☒	A full description of the statistical parameters including central tendency (e.g. means) or other basic estimates (e.g. regression coefficient) AND variation (e.g. standard deviation) or associated estimates of uncertainty (e.g. confidence intervals)
☒	☐	For null hypothesis testing, the test statistic (e.g. *F*, *t*, *r*) with confidence intervals, effect sizes, degrees of freedom and *P* value noted<br>*Give P values as exact values whenever suitable.*
☒	☐	For Bayesian analysis, information on the choice of priors and Markov chain Monte Carlo settings
☒	☐	For hierarchical and complex designs, identification of the appropriate level for tests and full reporting of outcomes
☒	☐	Estimates of effect sizes (e.g. Cohen's *d*, Pearson's *r*), indicating how they were calculated

Our web collection on statistics for biologists contains articles on many of the points above.

Software and code

Policy information about availability of computer code

Data collection	All presented data have been acquired using existing and routinely used software. LC-MS data was collected Bruker Compass qtofControl 5.2.109 / Hystar 5.1.5.1 or Compass qtofControl 6.3 / Hystar 6.0.30.0 software. NMR data was collected by Bruker TopSpin 3.6.1. Confocal laser microscopy images were collected using ZEN black 2.1 V.14.0.18.201 (Zeiss). RNA-seq data was sequenced on an Illumina NovaSeq 6000 PE150 platform.
Data analysis	All data analysis was done using routinely used software. Multiple sequence alignments not for phylogeny were made with MUSCLE 5.1 implemented in Geneious Prime 2024.0.3. For phylogenetic trees sequences were aligned with webPRANK (https://www.ebi.ac.uk/goldman-srv/webprank/; version updated 8 Oct 2017). Phylogenetic trees were constructed using IQ-Tree 2.3.6 (http://iqtree.cibiv.univie.ac.at/). Protein structural models were predicted using AlphaFold 3 (https://alphafoldserver.com). LC-MS data was analysed using MZmine 3.6.0 or 4.1.0 and Sirius v5.8.6. NMR data was analyzed with Bruker TopSpin 3.6.1. Chemical structures were generated in ChemDraw Professional 20.1.0.112. RNA-seq data was quality checked with FastQC (Galaxy Version 0.73), quality trimmed with Trimmomatic (Galaxy Version 0.38.1) and assembled with rnaSPAdes (Galaxy Version 3.15.4) all implemented on an in-house Galaxy server. Functional annotation of transcripts was performed on OmicsBox 3.1 (Biobam) using the SwissProt 2021 database and eggNOG-mapper. RNA-seq expression values were generated in CLC Genomics Workbench 21.0.4 (Qiagen). Confocal images were processed with ImageJ 1.54i. All graphs and heatmaps were prepared with GraphPad Prism 10.2.3 or 10.3.0. Main figures were assembled in Adobe Illustrator 27.8. Supplementary figures were assembled in PowerPoint 16.89.1.

For manuscripts utilizing custom algorithms or software that are central to the research but not yet described in published literature, software must be made available to editors and reviewers. We strongly encourage code deposition in a community repository (e.g. GitHub). See the Nature Portfolio guidelines for submitting code & software for further information.

Data

Policy information about availability of data

All manuscripts must include a data availability statement. This statement should provide the following information, where applicable:

- Accession codes, unique identifiers, or web links for publicly available datasets
- A description of any restrictions on data availability
- For clinical datasets or third party data, please ensure that the statement adheres to our policy

There are no restrictions on the availability of the data. Illumina RNA-seq raw reads have been uploaded in fastq format to NCBI Sequence Read Archive under BioProject PRJNA1169657 (https://www.ncbi.nlm.nih.gov/sra/PRJNA1169657). All reported gene sequences were deposited to NCBI Genbank under the accession numbers listed in Supplementary Table 2: AsDGD1 (PQ363556), AsDGD2 (PQ363557), AsDOMT1 (PQ363558), AsDOMT2 (PQ363559), AsDOMT3 (PQ363560), AsDOMT4 (PQ363561), AsDOMT5 (PQ363562), AsDOMT6 (PQ363563), AsDOMT7 (PQ363564), AsDPOMT1 (PQ363565), AsDPOMT2 (PQ363566), AsDR1 (PQ363567), AsDR2 (PQ363568), AsS6DGD (PQ363569), CiDE (PQ363570), CiDGD (PQ363571), CiDOMT1 (PQ363572), CiDOMT2 (PQ363573), CiDPOMT (PQ363574), CiDR1 (PQ363575), CiDR2 (PQ363576), CiIpS (PQ363577), CiS6DGD (PQ363578). Source data are provided with this paper. Sequences to construct phylogenetic trees were retrieved from public databases (https://blast.ncbi.nlm.nih.gov/Blast.cgi; https://db.cngb.org/onekp/); accession numbers and amino acid sequence are listed in Supplementary Dataset 1.

Research involving human participants, their data, or biological material

Policy information about studies with human participants or human data. See also policy information about sex, gender (identity/presentation), and sexual orientation and race, ethnicity and racism.

Reporting on sex and gender	Not applicable
Reporting on race, ethnicity, or other socially relevant groupings	Not applicable
Population characteristics	Not applicable
Recruitment	Not applicable
Ethics oversight	Not applicable

Note that full information on the approval of the study protocol must also be provided in the manuscript.

Field-specific reporting

Please select the one below that is the best fit for your research. If you are not sure, read the appropriate sections before making your selection.

☒ Life sciences ☐ Behavioural & social sciences ☐ Ecological, evolutionary & environmental sciences

For a reference copy of the document with all sections, see nature.com/documents/nr-reporting-summary-flat.pdf

Life sciences study design

All studies must disclose on these points even when the disclosure is negative.

Sample size	Prior determination of sample size is not applicable to this study. Nicotiana benthamiana pathway reconstitution experiments were done on three independent biological replicates which is standard for this type of experiments. Metabolite profiling by LC-MS analysis of Alangium salviifolium and Carapichea ipecacuanha was done on three biological replicates which is standard. RNA-seq was performed on one replicate which is sufficient for gene discovery as no statistics were applied. These standard sample sizes were similar as in other published studies for example https://www.nature.com/articles/s41586-022-04950-4.
Data exclusions	No data was excluded from the analyses.
Replication	Details about biological replicates are provided in the figure legends. Pathway reconstitution experiments were conducted on three biological replicates that correspond to three independent individual plants. All attempts of replication were successful.
Randomization	The order of all LC-MS samples was randomized prior to the runs. For pathway reconstitution Nicotinana benthamiana three biological replicates (=three independent individual plants) were used. Of each individual plant the youngest fully expanded leaf was used. Plants were grown in randomized order. Experiments were successfully repeated on other days. For other types of experiments of this study randomization is not relevant.
Blinding	Blinding was not relevant for this study; characterization of pathway genes and enzymes requires insight into the experimental conditions and characteristics of the samples.

Reporting for specific materials, systems and methods

We require information from authors about some types of materials, experimental systems and methods used in many studies. Here, indicate whether each material, system or method listed is relevant to your study. If you are not sure if a list item applies to your research, read the appropriate section before selecting a response.

Materials & experimental systems

n/a	Involved in the study	
☒	☐	Antibodies
☒	☐	Eukaryotic cell lines
☒	☐	Palaeontology and archaeology
☒	☐	Animals and other organisms
☒	☐	Clinical data
☒	☐	Dual use research of concern
☐	☒	Plants

Methods

n/a	Involved in the study	
☒	☐	ChIP-seq
☒	☐	Flow cytometry
☒	☐	MRI-based neuroimaging

Dual use research of concern

Policy information about dual use research of concern

Hazards

Could the accidental, deliberate or reckless misuse of agents or technologies generated in the work, or the application of information presented in the manuscript, pose a threat to:

No	Yes	
☒	☐	Public health
☒	☐	National security
☒	☐	Crops and/or livestock
☒	☐	Ecosystems
☒	☐	Any other significant area

Experiments of concern

Does the work involve any of these experiments of concern:

No	Yes	
☒	☐	Demonstrate how to render a vaccine ineffective
☒	☐	Confer resistance to therapeutically useful antibiotics or antiviral agents
☒	☐	Enhance the virulence of a pathogen or render a nonpathogen virulent
☒	☐	Increase transmissibility of a pathogen
☒	☐	Alter the host range of a pathogen
☒	☐	Enable evasion of diagnostic/detection modalities
☒	☐	Enable the weaponization of a biological agent or toxin
☒	☐	Any other potentially harmful combination of experiments and agents

Plants

Seed stocks	Nicotiana benthamiana seeds were obtained from seed stocks maintained by the greenhouse team at Max Planck Institute for Chemical Ecology, Jena. All other plants were obtained as plantlets, no seed stocks were obtained.
Novel plant genotypes	No stable transformation was carried out. Heterologous overexpression in N. benthamiana was done transiently through leaf agroinfiltration as described in the methods paragraph "A. tumefaciens mediated transient expression in N. benthamiana".
Authentication	Transient transformation of N. benthamiana through leaf agroinfiltration was done as described in the methods paragraph "A. tumefaciens mediated transient expression in N. benthamiana". No stable transformation was carried out.
